# One-spot synthesis of FeOOH/rGO composites by ferrous-ion-induced self-assembly of graphene oxides with different degrees of oxidation

**DOI:** 10.1371/journal.pone.0246386

**Published:** 2021-02-01

**Authors:** Yang Hu, Qingyang Yang, Yingyu Gu

**Affiliations:** School of Civil and Resources Engineering, University of Science and Technology Beijing, Beijing, China; King Saud University, SAUDI ARABIA

## Abstract

In this study, graphene oxide sheets with different oxidation degrees were reduced by ferrous ion for coating FeOOH nano particles on reduced graphene oxide (rGO) matrix to synthesize FeOOH/rGO composites. The effect of the degree of oxidation on the morphology and chemical structure of FeOOH/rGO was studied using scanning electron microscopy, Raman spectroscopy, thermogravimetric analysis, and Brunauer-Emmett-Teller surface area analysis. The particle size of FeOOH crystallites was approximately 100 nm, and they were distributed uniformly on the surface and in the pores of FeOOH/rGO. FeOOH/rGO prepared with mildly oxidized graphite had fewer defects, higher specific surface area, and higher FeOOH content than FeOOH/rGO prepared with highly oxidized graphite. These features resulted in better electrochemical properties, such as larger specific capacitance and lower charge transfer resistance.

## 1. Introduction

Graphene-based matrix materials are the macroscopic congeries of single or multilayer rGO. Unique properties, such as high specific surface, high porosity, and good electrical conductivity, afford these materials with immense potential for application in the fields of supercapacitors, bio-catalysis, photocatalysis, thermal insulation, and water pollution absorbents [1–5]. Graphene oxide, the precursor of rGO, has an abundance of oxygen-containing functional groups, including carboxyl, hydroxy, and epoxy, which enables good dispersibility in aqueous solutions, and creates strong affinities for metal ions [6–8]. These features allow a facile method for chemical deposition of metallic oxides on graphene, forming a self-assembled, three-dimensional graphene hydrogel. This may enhance the original advantages of graphene-based porous materials and create many new functionalities [9–11].

Numerous types of metal oxide nanoparticles, including ferric oxide, titanic oxide, and manganic oxide nanoparticles, have been successfully loaded on the surface of rGO [12–14]. Compared to other metal oxides, FeOOH is inexpensive, environmentally friendly, and easy to synthesize, and therefore, has considerable potential for clean energy applications [15]. Although graphene-based porous materials are generally selected for supercapacitor electrodes, owing to their good double-layer energy storage capacities, their low specific capacitances limit further development and application as energy storage materials [16]. Considering the high reversible capacity and wide operating potential windows of FeOOH, loading FeOOH nanoparticles on rGO may create synergistic benefits and form FeOOH/rGO composites with integrated functionalities. Qi et al. prepared FeOOH rod/rGO composites that exhibited high energy densities at different current densities [17]. FeOOH nanoparticles can improve the electrochemical properties of rGO, but the improvement in electrochemical properties cannot be controlled because the structure of rGO remained unchanged.

The electrical properties of rGO are mainly affected by defects like vacancies and residual oxygen groups on its surface, and the electrical conductivity is inversely proportional to the defect degree [18]. Therefore, preparing rGO from a graphene oxide (GO) with a low degree of oxidation may help protect its graphitized structure.

In this study, the effect of the degree of oxidation of GO on the structure and electrochemical performance of FeOOH/rGO was investigated. The objective was to understand the synthesis of FeOOH/rGO using GO with different degrees of oxidation, and to improve the electrochemical performance of FeOOH/rGO by controlling defect degree of GO.

## 2. Experimental

### 2.1. Materials

The purified natural flake graphite (particle size < 60 μm) used in this study was purchased from Xianfeng Nanomaterials Technology Co. Ltd. (China). Analytical-grade chemicals used for preparing rGO were supplied by Sinopharm Chemical Reagent Co. Ltd. (China).

### 2.2. Synthesis of FeOOH/rGO

Highly oxidized and mildly oxidized graphite samples were prepared by an improved Hummer’s method with 6 g/g (KMnO_4_: graphite) and 2 g/g KMnO_4_, respectively [19]. 1.5g graphite powders was mixed with 200 ml of concentrated H_2_SO_4_/H_3_PO_4_ mixture (ratio 9:1) in an ice water bath (5–10°C) for 10 min, followed by a slow addition of KMnO4 powder with a given dosage. Then the oxidation process was carried out under 50°C water bath for 2h with 400 rev/min stirring. After that, 3 ml of 30% H_2_O_2_ was added and the reaction product was washed by water until the pH closed to neutral. The solid obtained was dried in a vacuum oven at 60°C to achieve purified graphite oxide powder. Aqueous suspensions of highly oxidized graphite and mildly oxidized graphite (0.05 wt.%) were treated with an Elma P30HSE ultrasonic cleaner in the presence of 1×10^−6^ mol/L sodium laurate at pH 10 for 30 min. The obtained dispersions were centrifuged at 3500 rpm for 30 min to separate any unexfoliated solid. The centrifugal supernatants prepared from highly oxidized graphite and mildly oxidized graphite with surfactant-assisted ultrasonic exfoliation are denoted as GO-1 and GO-2, respectively. To synthesize FeOOH/rGO, 40 mL of 0.5 mg/mL GO suspension was mixed with 0.5 mmol Fe_2_SO_4_ and the pH of the suspension was adjusted to 3. The mixture was then heated in a 90°C water bath for 6 h. The resulting FeOOH/rGO composites were then washed with ultrapure water and dried using freeze drying. The FeOOH/rGO composites prepared from GO-1 and GO-2 are denoted as FeOOH/rGO-1 and FeOOH/rGO-2, respectively.

### 2.3. Characterization

Field-emission scanning electron microscopy (SEM, Zeiss Ultra Plus, Germany) was used to characterize the morphologies of rGO and FeOOH/rGO, and energy-dispersive spectroscopy (EDS) was used to analyze chemical compositions. The specific surface areas of rGO and FeOOH/rGO were analyzed by the Brunauer-Emmett-Teller (BET) method using a Micromeritics ASAP 2460 surface area and porosity analyzer (USA). Raman spectroscopy (DXR2 Raman Microscope, USA) was used to quantify the reduction of GO and measure the defect degree of FeOOH/rGO. The morphologies and thicknesses of GO-1 and GO-2 were measured using atomic force microscopy (AFM, Bruker Dimension Icon, USA). The AFM samples was prepared by depositing the diluted aqueous GO colloidal solution onto the freshly cleaved mica flakes and then dried the mica flakes at 60°C for 2h. The weight ratios of FeOOH to rGO were determined using thermogravimetric analysis (TGA) using a PerkinElmer STA 8000 instrument (Netherlands). Samples were heated from 30 to 1000°C at a heating rate of 10°C in N_2_.

### 2.4. Electrochemical tests

The electrochemical properties of FeOOH/rGO were measured using a Princeton Versa STAT 4 electrochemical workstation. The FeOOH/rGO was mixed with acetylene black and poly(tetrafluoroethene) at a mass ratio of 8:1:1, and the mixture was placed at the center of two nickel foam substrates and compressed into the working electrode. This electrode was immersed in a Na_2_SO_4_ solution (1 M) with a reference electrode and counter electrode. The electrochemical impedance of FeOOH/rGO was tested at 5 mV ac perturbation from 100 kHz to 0.1 Hz at open circuit potential. Cyclic voltammetry (CV) experiments were carried out at a scan rate of 100 mV/s from −1 to 0 V, and the specific capacitances (*Cs*) were calculated using the equation described in our previous paper [20].

## 3. Results and discussion

**Fig 1:**
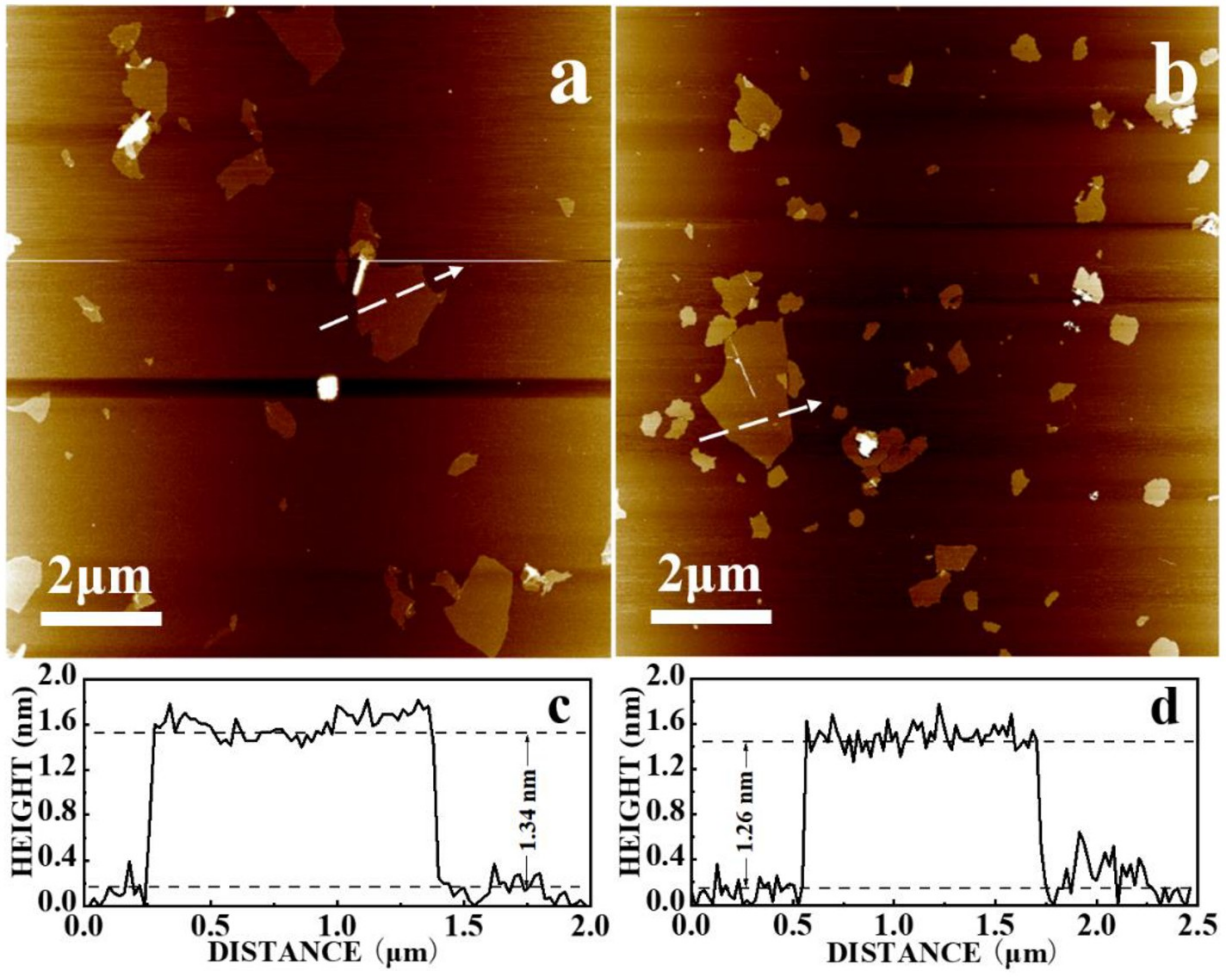
AFM images of (a) GO-1 and (b)GO-2. Height profiles of (c) GO-1 and (d) GO-2 sheets taken along the white lines in (a) and (b), respectively.

It is known that the electronic properties of graphene are heavily dependent on the number of graphene layers. Thicker graphene sheets have more complicated band structures. As the precursor of rGO, graphene oxide should therefore be as thin as possible. AFM images of GO-1 and GO-2 (Fig 1a and 1b, respectively) indicated that the GO sheets were plate-like and should be single-layer as the thicknesses of them were 1.34 and 1.26 nm, respectively.

**Fig 2:**
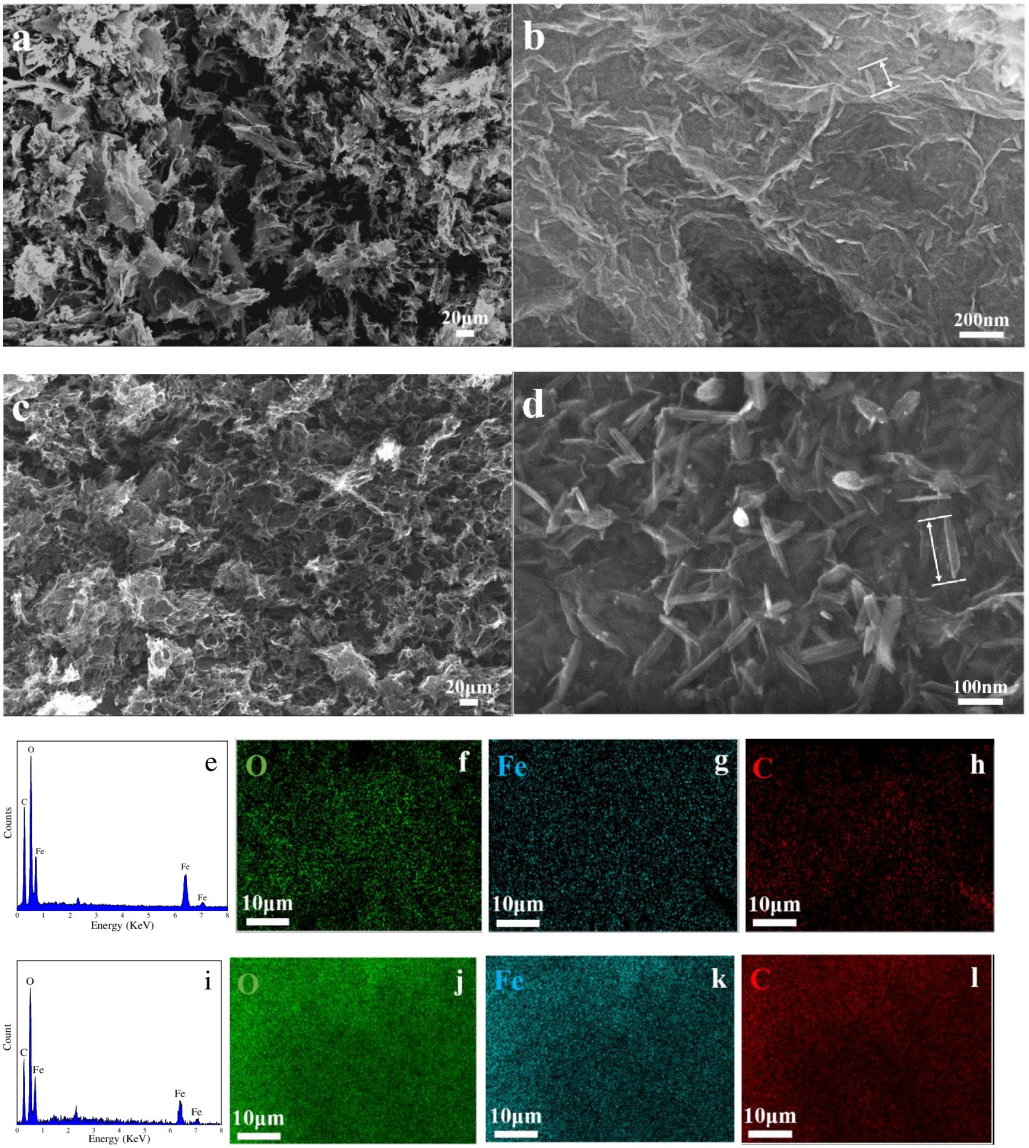
SEM images of (a, b) FeOOH/rGO-1 and (c, d) FeOOH/rGO-2; (e) EDS data of FeOOH/rGO-1 and EDS mapping of O (f), Fe (g) and C (h) elements, (i) EDS data of FeOOH/rGO-2 and EDS mapping of O (j), Fe (k) and C (l) elements.

To determine the effect of the degree of oxidation of GO on the formation of FeOOH/rGO composites, the porous structures of FeOOH/rGO-1 and FeOOH/rGO-2 were analyzed by scanning electron microscopy. Fig 2a and 2c show that both FeOOH/rGO-1 and FeOOH/rGO-2 have a complex macroscopic porous structure composed of rGO sheets. The pore size of FeOOH/rGO-2 was smaller than that of FeOOH/rGO-1, which might be attributed to a stronger π-π interaction between sp^2^-hybridized carbon aromatic structures in rGO-2 sheets. EDS analysis revealed the presence of C, O, and Fe elements, and EDS mapping showed that these elements were uniformly distributed in the FeOOH/rGO composites. Fig 2b and 2d also show that the length of the needle-like FeOOH nanoparticles was approximately 100 nm, and they were distributed not only on the rGO surface but also in the pores of the rGO aerogel, which increased the specific surface area of FeOOH/rGO and the number of mesopores.

**Fig 3:**
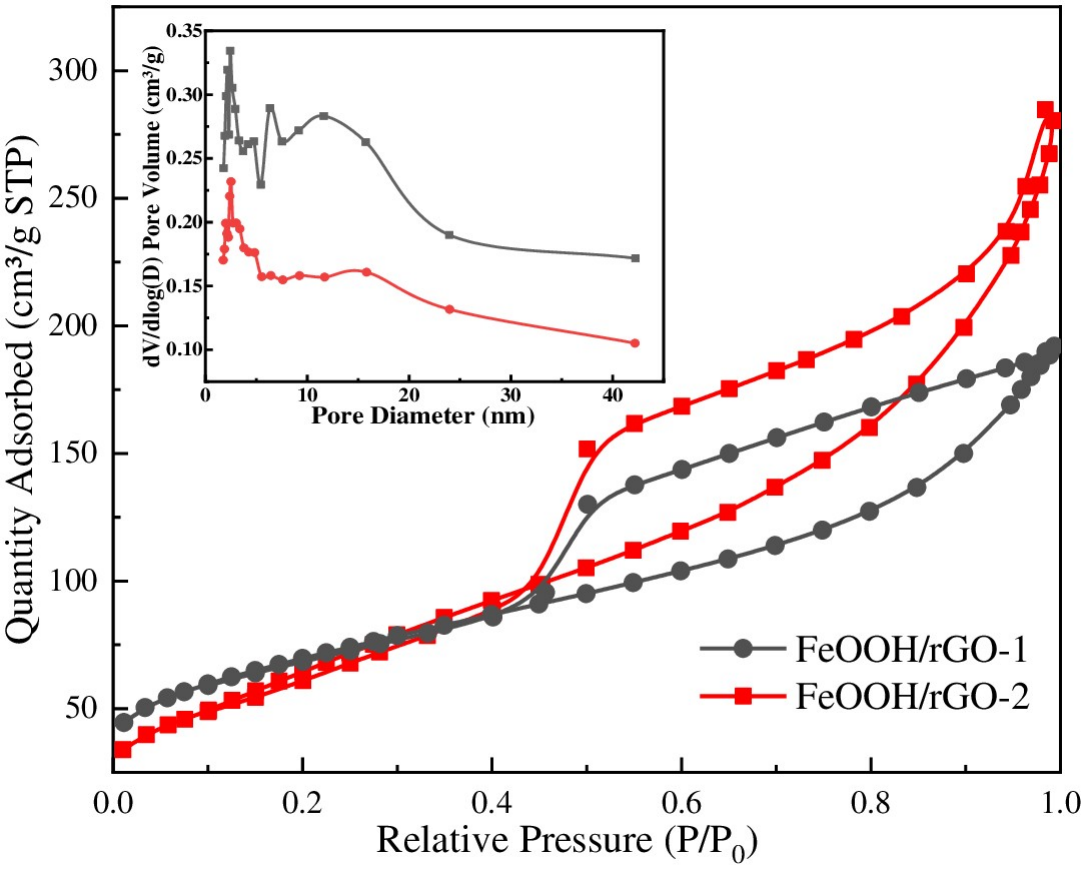
Nitrogen adsorption/desorption isotherms of FeOOH/rGO and the corresponding BJH pore size distributions.

Nitrogen adsorption/desorption isotherms of FeOOH/rGO-1 and FeOOH/rGO-2 were recorded (Fig 3) to elucidate the effect of the degree of oxidation of GO on the specific surface area and pore size distribution of FeOOH/rGO. Both samples had type IV hysteresis loops, indicating that the pore structures were mainly slit-like and wedge-shaped mesopores. The pore size distributions of FeOOH/rGO were analyzed using the Barrett-Joyner-Halenda (BJH) adsorption data, and the results are presented in Table 1 [21]. The BET specific surface area of FeOOH/rGO-2 (257.73 m^2^/g) was slightly larger than that of FeOOH/rGO-1 (246.04 m^2^/g), while the average pore size of FeOOH/rGO-2 (4.82 nm) was smaller than that of FeOOH/rGO-1 (6.73 nm). This may be due to a more intact graphitized structure in the rGO-2 sheets prepared from mildly oxidized graphite, resulting in stronger hydrophobic and π-π interactions between rGO-2 sheets, producing a denser porous structure.

**Table 1:** BET specific surface areas, BJH average pore sizes, and pore volumes of FeOOH/rGO-1 and FeOOH/rGO-2.

Materials	Surface area (cm^2^g^-1^)	BJH average pore size (nm)	Pore volume (cm^3^g^-1^)
FeOOH/rGO-1	246.04	6.73	0.43
FeOOH/rGO-2	257.73	4.82	0.26

**Fig 4:**
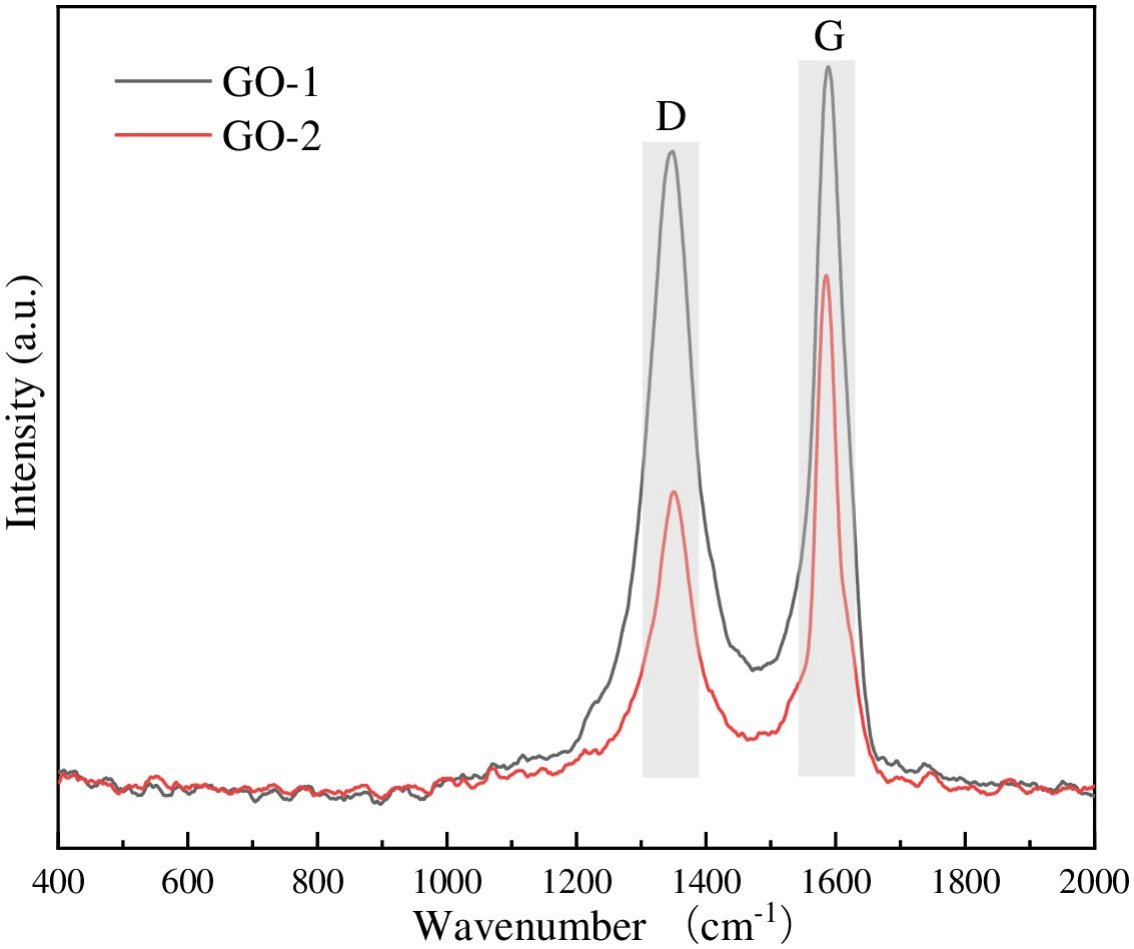
Raman spectra of GO-1 and GO-2.

**Fig 5:**
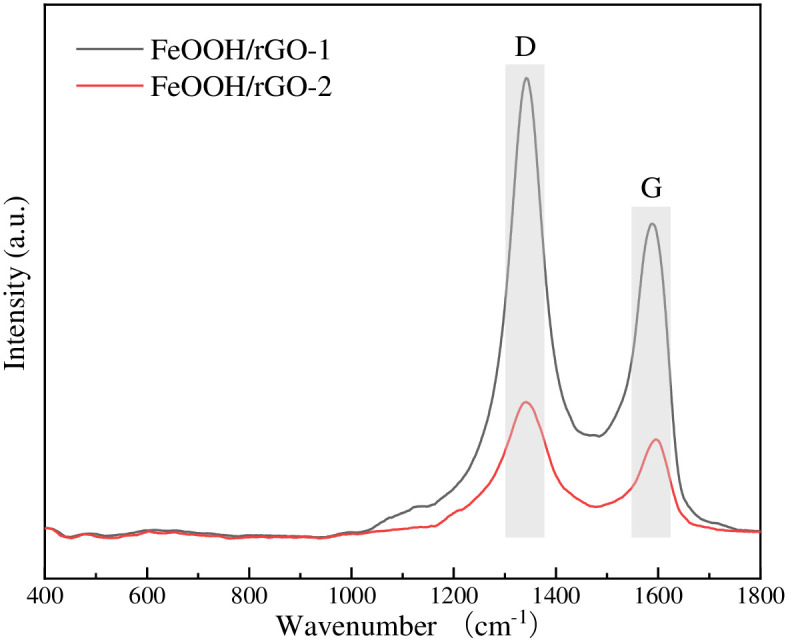
Raman spectra of FeOOH/rGO-1 and FeOOH/rGO-2.

Raman spectra of GO and FeOOH/rGO were recorded (Figs 4 and 5) to analyze changes in the chemical structure of GO during the reduction process. Both GO-1 and GO-2 showed two peaks at 1347.74 cm^-1^ (D peak) and 1588.87 cm^-1^ (G peak), derived from the lattice motion and in-plane motion of carbon atoms, respectively [22]. Generally, the presence of the D peak indicates defects (oxidation functional groups, vacancies or edges) in GO and FeOOH/rGO, while the G peak indicates a graphitic structure. The ratio of the intensities of D and G peaks is typically used to evaluate the defect degree of graphene-based materials. I_D_/I_G_ was 0.86 for GO-1 and 0.58 for GO-2, indicating that the defect degree (or oxidation degree) of GO-1 was higher than that of GO-2 [23]. After reduction, the I_D_/I_G_ values of FeOOH/rGO-1 (1.47) and FeOOH/rGO-2 (1.34) both increased significantly, indicating successful reduction of GO.

**Fig 6:**
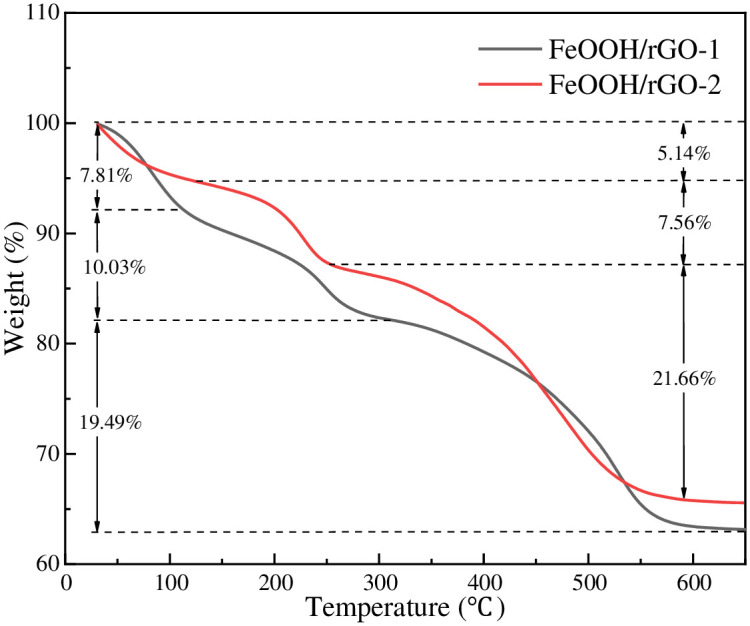
TGA curves of FeOOH/rGO-1 and FeOOH/rGO-2.

To quantitatively evaluate the amount of FeOOH nanoparticles coated on the surface of rGO, thermogravimetric analysis was carried out on the two FeOOH/rGO samples. Fig 6 presents TGA curves which showed a small weight loss for both FeOOH/rGO-1 and FeOOH/rGO-2 when heated to approximately 130°C. This was attributed to detachment of physically absorbed free water. The amount of water liberated in FeOOH/rGO-1 was higher than that of FeOOH/rGO-2, which might be attributed to more residual hydrophilic oxygen-containing groups in FeOOH/rGO-1. The mass loss in the temperature range 130–300°C was mainly due to the decomposition of FeOOH and the evaporation of the interlamellar water in rGO [24]. When the temperature exceeded 300°C, rGO dissociated into CO and CO_2_ in air. After heating, 62.67 wt.% remained from FeOOH/rGO-1, and 65.64 wt.% Fe_2_O_3_ remained from FeOOH/rGO-2, indicating that there were about 69.72 wt.% and 73.02 wt.% FeOOH in FeOOH/rGO-1 and FeOOH/rGO-2, respectively.

**Fig 7:**
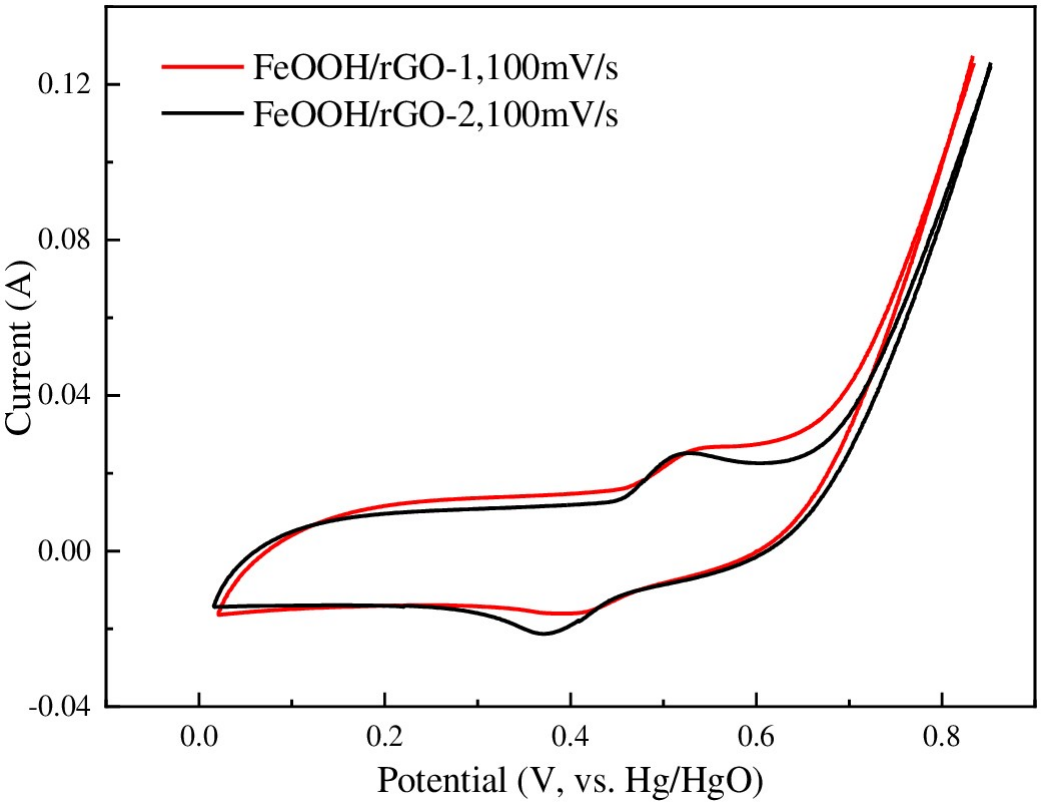
The CV curves of FeOOH/rGO-1 and FeOOH/rGO-2.

**Fig 8:**
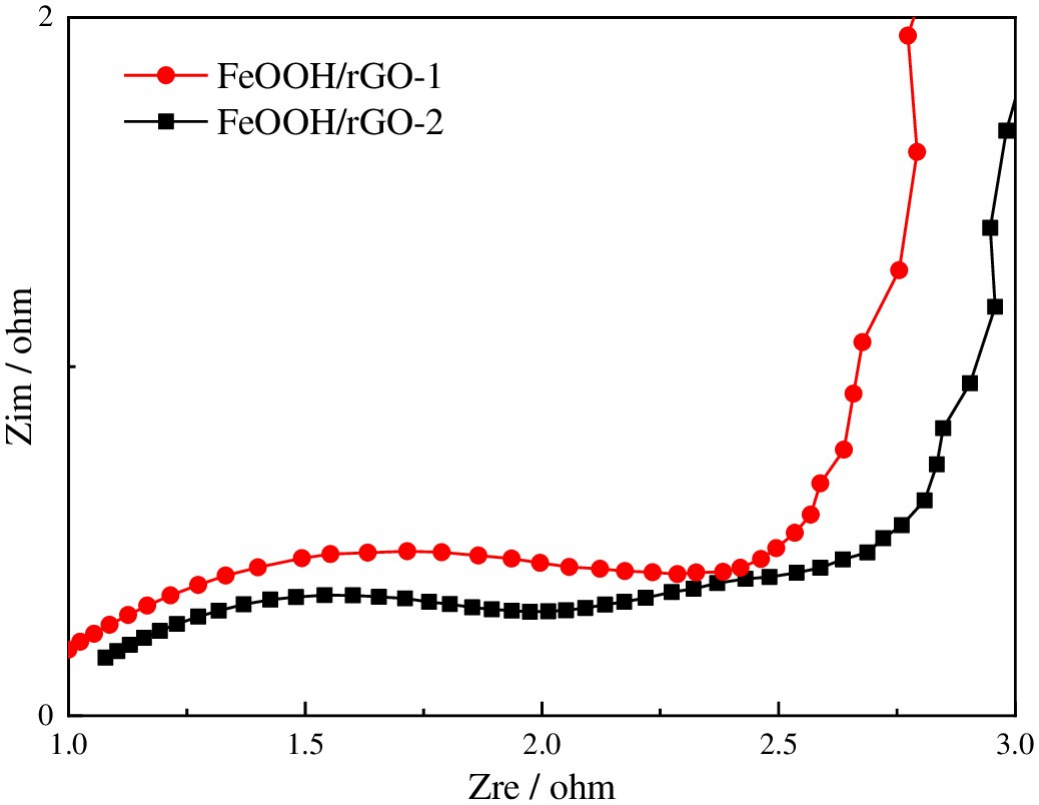
Electrochemical impedance spectroscopy curves of FeOOH/rGO-1 and FeOOH/rGO-2.

The electrochemical performances of FeOOH/rGO-1 and FeOOH/rGO-2 were tested using a three-electrode system within the potential range from 0 to +0.8 V at a scan rate of 100 mV/s. Fig 7 shows the CV curves of FeOOH/rGO-1 and FeOOH/rGO-2. There was an obvious oxidation peak at 0.5–0.6 V and a reduction peak at 0.3–0.4 V. The redox peak of FeOOH/rGO-2 was clearer than that of FeOOH/rGO-1, which might be attributed to the higher FeOOH content in FeOOH/rGO-2, because the electrochemical capacitance of FeOOH/rGO was mainly derived from the pseudo-capacitance of FeOOH. The specific capacitance of FeOOH/rGO-1 was calculated to be 86.46 F/g, larger than that of FeOOH/rGO-2 (72.2 F/g). The intrinsic kinetics of FeOOH/rGO in the electrochemical reaction were tested using electrochemical impedance spectroscopy (EIS). Fig 8 shows that both EIS curves were depressed semicircles, attributed the charge transfer in FeOOH/rGO. The semicircle for FeOOH/rGO-1 had a larger diameter than that for FeOOH/rGO-2, indicating that the charge transfer resistance of FeOOH/rGO-1 was higher. This could be attributed to the greater number of defects in FeOOH/rGO-1.

## 4. Conclusions

FeOOH/rGO composites were successfully prepared by growing ~100-nm FeOOH nanoparticles on rGO. The rGO was reduced from GOs with different degrees of oxidation. It was found that decreasing the degree of oxidation of GO may reduce the defect degree of FeOOH/rGO, while increasing its specific surface area and the amount of FeOOH nanoparticles. These features resulted in better electrochemical properties, such as larger specific capacitance and lower charge transfer resistance.

## Supporting information

S1 File (DOCX)
