# Supplementary material for: One-spot synthesis of FeOOH/rGO composites by ferrous-ion-induced self-assembly of graphene oxides with different degrees of oxidation
Source: PLoS One. 2021 Feb 1;16(2):e0246386. doi: 10.1371/journal.pone.0246386 (PMC7850491; doi:10.1371/journal.pone.0246386)
Supplement: S1 File — (DOCX) [file pone.0246386.s001.docx]

Data for Fig. 3:

Nitrogen adsorption/desorption isotherms of FeOOH/rGO

| FeOOH/rGO-1 | | FeOOH/rGO-2 | |
| --- | --- | --- | --- |
| Relative Pressure （P/P0） | Quantity Adsorbed (cm³/g STP) | Relative Pressure （P/P0） | Quantity Adsorbed (cm³/g STP) |
| 0.01047 | 33.9459 | 0.01148 | 44.5336 |
| 0.03518 | 39.8022 | 0.03381 | 50.4153 |
| 0.05818 | 43.5773 | 0.05713 | 54.2167 |
| 0.07565 | 45.8093 | 0.07491 | 56.6075 |
| 0.10076 | 49.3503 | 0.10002 | 59.6695 |
| 0.12564 | 53.1847 | 0.12505 | 62.4056 |
| 0.15051 | 56.9325 | 0.15001 | 64.9092 |
| 0.17538 | 60.7029 | 0.17502 | 67.2887 |
| 0.20023 | 64.4127 | 0.2 | 69.6483 |
| 0.2251 | 68.15 | 0.22503 | 71.8682 |
| 0.24996 | 71.8817 | 0.25001 | 73.999 |
| 0.2748 | 75.1989 | 0.27504 | 76.2627 |
| 0.29966 | 78.8638 | 0.30001 | 78.5471 |
| 0.3496 | 85.7178 | 0.34957 | 82.6431 |
| 0.39951 | 92.3261 | 0.39949 | 86.7779 |
| 0.44939 | 98.6929 | 0.44941 | 90.9379 |
| 0.49924 | 105.1525 | 0.49923 | 95.04 |
| 0.54912 | 112.0534 | 0.54912 | 99.3298 |
| 0.59897 | 119.4866 | 0.59909 | 103.9471 |
| 0.64887 | 126.8969 | 0.64902 | 108.6267 |
| 0.69867 | 136.6858 | 0.6988 | 113.8964 |
| 0.74859 | 147.1999 | 0.74866 | 119.9091 |
| 0.79838 | 160.1078 | 0.79855 | 127.282 |
| 0.84819 | 177.103 | 0.84831 | 136.6248 |
| 0.89799 | 199.3652 | 0.89784 | 149.9828 |
| 0.94784 | 227.5556 | 0.94732 | 168.953 |
| 0.95832 | 236.6744 | 0.95879 | 175.0025 |
| 0.96836 | 245.5129 | 0.96869 | 179.9908 |
| 0.97836 | 255.1143 | 0.97866 | 184.4352 |
| 0.98814 | 267.4868 | 0.98854 | 188.4683 |
| 0.99306 | 280.3584 | 0.99373 | 191.9388 |
| 0.98408 | 284.6635 | 0.98461 | 189.8632 |
| 0.96344 | 254.5974 | 0.96231 | 185.7338 |
| 0.94268 | 237.0072 | 0.94197 | 183.529 |
| 0.9009 | 220.2955 | 0.90089 | 179.2094 |
| 0.83235 | 203.5687 | 0.85055 | 173.8267 |
| 0.78196 | 194.5727 | 0.80054 | 168.2081 |
| 0.73192 | 186.7253 | 0.75067 | 162.2246 |
| 0.70044 | 182.3904 | 0.70059 | 156.1596 |
| 0.65046 | 175.3311 | 0.65055 | 149.9674 |
| 0.60038 | 168.436 | 0.60065 | 143.689 |
| 0.5504 | 161.6746 | 0.55057 | 137.6435 |
| 0.50051 | 151.7213 | 0.50102 | 130.0403 |
| 0.4527 | 98.2332 | 0.45634 | 95.4645 |
| 0.40054 | 88.5384 | 0.40096 | 85.8494 |
| 0.33163 | 78.5979 | 0.33214 | 79.5667 |
| 0.28142 | 72.1047 | 0.28181 | 75.3874 |
| 0.25037 | 67.8228 | 0.25047 | 72.7028 |
| 0.2003 | 60.8683 | 0.20033 | 68.4846 |
| 0.15023 | 54.3883 | 0.15036 | 63.9229 |
| 0.10027 | 48.7345 | 0.10034 | 59.0474 |

The corresponding BJH pore size distributions

| FeOOH/rGO-1 | | FeOOH/rGO-2 | |
| --- | --- | --- | --- |
| Pore Diameter (nm) | dV/dlog(D) Pore Volume (cm³/g) | Pore Diameter (nm) | dV/dlog(D) Pore Volume (cm³/g) |
| 42.2427 | 0.17166 | 42.19738 | 0.10513 |
| 23.95172 | 0.18999 | 23.98572 | 0.13167 |
| 15.74993 | 0.26274 | 15.83595 | 0.16074 |
| 11.62106 | 0.28321 | 11.71137 | 0.15697 |
| 9.15829 | 0.27189 | 9.24354 | 0.15807 |
| 7.51584 | 0.26326 | 7.59992 | 0.15476 |
| 6.33874 | 0.28942 | 6.42278 | 0.15819 |
| 5.44847 | 0.22936 | 5.53173 | 0.15728 |
| 4.74766 | 0.26332 | 4.82943 | 0.17613 |
| 4.17824 | 0.26114 | 4.25939 | 0.17654 |
| 3.70338 | 0.25555 | 3.78468 | 0.17983 |
| 3.29828 | 0.26399 | 3.37945 | 0.19472 |
| 2.94538 | 0.28877 | 3.02639 | 0.19938 |
| 2.63193 | 0.30537 | 2.71429 | 0.19924 |
| 2.42871 | 0.33436 | 2.51149 | 0.23181 |
| 2.29333 | 0.26858 | 2.37522 | 0.22035 |
| 2.16278 | 0.31946 | 2.24385 | 0.18818 |
| 2.036 | 0.29877 | 2.11634 | 0.191 |
| 1.91204 | 0.26763 | 1.99167 | 0.19918 |
| 1.78958 | 0.24236 | 1.86859 | 0.17897 |
| -- | -- | 1.74559 | 0.17026 |

Data for Fig. 4:

| GO-1 | | GO-2 | |
| --- | --- | --- | --- |
| Wavenumber （cm-1） | Intensity (a.u.) | Wavenumber （cm-1） | Intensity (a.u.) |
| 408.7763 | 1.49574 | 408.7763 | 1.14779 |
| 409.7405 | 1.47996 | 409.7405 | 1.07269 |
| 410.7048 | 1.42342 | 410.7048 | 0.99318 |
| 411.669 | 1.34192 | 411.669 | 0.92651 |
| 412.6332 | 1.2641 | 412.6332 | 0.88492 |
| 413.5975 | 1.20626 | 413.5975 | 0.86481 |
| 414.5617 | 1.18082 | 414.5617 | 0.85072 |
| 415.5259 | 1.19128 | 415.5259 | 0.84441 |
| 416.4902 | 1.24016 | 416.4902 | 0.85937 |
| 417.4544 | 1.3032 | 417.4544 | 0.90135 |
| 418.4186 | 1.34495 | 418.4186 | 0.95228 |
| 419.3829 | 1.34047 | 419.3829 | 0.98404 |
| 420.3471 | 1.31364 | 420.3471 | 0.99864 |
| 421.3113 | 1.31095 | 421.3113 | 1.00911 |
| 422.2756 | 1.3359 | 422.2756 | 1.03221 |
| 423.2398 | 1.37337 | 423.2398 | 1.08095 |
| 424.204 | 1.39508 | 424.204 | 1.13015 |
| 425.1683 | 1.39915 | 425.1683 | 1.15523 |
| 426.1325 | 1.39885 | 426.1325 | 1.13524 |
| 427.0967 | 1.38784 | 427.0967 | 1.0668 |
| 428.061 | 1.33078 | 428.061 | 0.94713 |
| 429.0252 | 1.22725 | 429.0252 | 0.80925 |
| 429.9894 | 1.10575 | 429.9894 | 0.70787 |
| 430.9537 | 1.01254 | 430.9537 | 0.672 |
| 431.9179 | 0.97633 | 431.9179 | 0.69974 |
| 432.8821 | 0.96621 | 432.8821 | 0.7398 |
| 433.8464 | 0.95095 | 433.8464 | 0.76888 |
| 434.8106 | 0.91841 | 434.8106 | 0.78969 |
| 435.7748 | 0.87808 | 435.7748 | 0.8056 |
| 436.7391 | 0.83685 | 436.7391 | 0.81183 |
| 437.7033 | 0.79766 | 437.7033 | 0.80029 |
| 438.6675 | 0.77519 | 438.6675 | 0.77924 |
| 439.6318 | 0.76579 | 439.6318 | 0.74099 |
| 440.596 | 0.75489 | 440.596 | 0.67705 |
| 441.5602 | 0.72458 | 441.5602 | 0.59629 |
| 442.5245 | 0.66417 | 442.5245 | 0.52522 |
| 443.4887 | 0.55051 | 443.4887 | 0.48452 |
| 444.4529 | 0.4212 | 444.4529 | 0.46772 |
| 445.4172 | 0.323 | 445.4172 | 0.47058 |
| 446.3814 | 0.2673 | 446.3814 | 0.49342 |
| 447.3456 | 0.23475 | 447.3456 | 0.5303 |
| 448.3099 | 0.19388 | 448.3099 | 0.57291 |
| 449.2741 | 0.14266 | 449.2741 | 0.61408 |
| 450.2383 | 0.09611 | 450.2383 | 0.64608 |
| 451.2026 | 0.05892 | 451.2026 | 0.65326 |
| 452.1668 | 0.05306 | 452.1668 | 0.62632 |
| 453.131 | 0.09205 | 453.131 | 0.5803 |
| 454.0953 | 0.15918 | 454.0953 | 0.54673 |
| 455.0595 | 0.19605 | 455.0595 | 0.51856 |
| 456.0237 | 0.20199 | 456.0237 | 0.48592 |
| 456.988 | 0.19425 | 456.988 | 0.45744 |
| 457.9522 | 0.18651 | 457.9522 | 0.44567 |
| 458.9164 | 0.1842 | 458.9164 | 0.45106 |
| 459.8807 | 0.20893 | 459.8807 | 0.47123 |
| 460.8449 | 0.27505 | 460.8449 | 0.51247 |
| 461.8091 | 0.36855 | 461.8091 | 0.56659 |
| 462.7734 | 0.46695 | 462.7734 | 0.61072 |
| 463.7376 | 0.54719 | 463.7376 | 0.61694 |
| 464.7018 | 0.59409 | 464.7018 | 0.58323 |
| 465.6661 | 0.58963 | 465.6661 | 0.5257 |
| 466.6303 | 0.55872 | 466.6303 | 0.4638 |
| 467.5945 | 0.55059 | 467.5945 | 0.41338 |
| 468.5588 | 0.59538 | 468.5588 | 0.37858 |
| 469.523 | 0.68328 | 469.523 | 0.35739 |
| 470.4872 | 0.75066 | 470.4872 | 0.36341 |
| 471.4514 | 0.78536 | 471.4514 | 0.40038 |
| 472.4157 | 0.80462 | 472.4157 | 0.43475 |
| 473.3799 | 0.82187 | 473.3799 | 0.4362 |
| 474.3441 | 0.8353 | 474.3441 | 0.393 |
| 475.3084 | 0.81786 | 475.3084 | 0.3343 |
| 476.2726 | 0.75242 | 476.2726 | 0.27695 |
| 477.2368 | 0.69987 | 477.2368 | 0.24645 |
| 478.2011 | 0.6992 | 478.2011 | 0.256 |
| 479.1653 | 0.74393 | 479.1653 | 0.29133 |
| 480.1295 | 0.78132 | 480.1295 | 0.31232 |
| 481.0938 | 0.76669 | 481.0938 | 0.28902 |
| 482.058 | 0.72351 | 482.058 | 0.24044 |
| 483.0222 | 0.65811 | 483.0222 | 0.16972 |
| 483.9865 | 0.57022 | 483.9865 | 0.07613 |
| 484.9507 | 0.47233 | 484.9507 | -0.02899 |
| 485.9149 | 0.4012 | 485.9149 | -0.11382 |
| 486.8792 | 0.37154 | 486.8792 | -0.16206 |
| 487.8434 | 0.33099 | 487.8434 | -0.20717 |
| 488.8076 | 0.25658 | 488.8076 | -0.26117 |
| 489.7719 | 0.14871 | 489.7719 | -0.31853 |
| 490.7361 | 0.02367 | 490.7361 | -0.36947 |
| 491.7003 | -0.10982 | 491.7003 | -0.42815 |
| 492.6646 | -0.18692 | 492.6646 | -0.47085 |
| 493.6288 | -0.17302 | 493.6288 | -0.46739 |
| 494.593 | -0.10323 | 494.593 | -0.42234 |
| 495.5573 | -0.04131 | 495.5573 | -0.35221 |
| 496.5215 | -0.0345 | 496.5215 | -0.2653 |
| 497.4857 | -0.06982 | 497.4857 | -0.18778 |
| 498.45 | -0.13861 | 498.45 | -0.16127 |
| 499.4142 | -0.21968 | 499.4142 | -0.16674 |
| 500.3784 | -0.28041 | 500.3784 | -0.16102 |
| 501.3427 | -0.29388 | 501.3427 | -0.1113 |
| 502.3069 | -0.26178 | 502.3069 | -0.03198 |
| 503.2711 | -0.2145 | 503.2711 | 0.02029 |
| 504.2354 | -0.16526 | 504.2354 | 0.04565 |
| 505.1996 | -0.11873 | 505.1996 | 0.06581 |
| 506.1638 | -0.07635 | 506.1638 | 0.09837 |
| 507.1281 | -0.03801 | 507.1281 | 0.14525 |
| 508.0923 | 2.62E-04 | 508.0923 | 0.19633 |
| 509.0565 | 0.02909 | 509.0565 | 0.23334 |
| 510.0208 | 0.02856 | 510.0208 | 0.23678 |
| 510.985 | -0.0155 | 510.985 | 0.19873 |
| 511.9492 | -0.0996 | 511.9492 | 0.1356 |
| 512.9135 | -0.2019 | 512.9135 | 0.08852 |
| 513.8777 | -0.30513 | 513.8777 | 0.08487 |
| 514.842 | -0.38303 | 514.842 | 0.10584 |
| 515.8062 | -0.41425 | 515.8062 | 0.13309 |
| 516.7704 | -0.40831 | 516.7704 | 0.16043 |
| 517.7347 | -0.39701 | 517.7347 | 0.19179 |
| 518.6989 | -0.41173 | 518.6989 | 0.23208 |
| 519.6631 | -0.44321 | 519.6631 | 0.25632 |
| 520.6274 | -0.48784 | 520.6274 | 0.22537 |
| 521.5916 | -0.54199 | 521.5916 | 0.14315 |
| 522.5558 | -0.59185 | 522.5558 | 0.05167 |
| 523.5201 | -0.61795 | 523.5201 | 0.00446 |
| 524.4843 | -0.59918 | 524.4843 | 0.01234 |
| 525.4485 | -0.5618 | 525.4485 | 0.03474 |
| 526.4128 | -0.55996 | 526.4128 | 0.03335 |
| 527.377 | -0.58653 | 527.377 | 0.00824 |
| 528.3412 | -0.59991 | 528.3412 | -0.02441 |
| 529.3055 | -0.55454 | 529.3055 | -0.04322 |
| 530.2697 | -0.47915 | 530.2697 | -0.04926 |
| 531.2339 | -0.41888 | 531.2339 | -0.03693 |
| 532.1982 | -0.34706 | 532.1982 | 0.01331 |
| 533.1624 | -0.24029 | 533.1624 | 0.11859 |
| 534.1266 | -0.10183 | 534.1266 | 0.26953 |
| 535.0909 | 0.02384 | 535.0909 | 0.41573 |
| 536.0551 | 0.10108 | 536.0551 | 0.50145 |
| 537.0193 | 0.14765 | 537.0193 | 0.54057 |
| 537.9836 | 0.19011 | 537.9836 | 0.57355 |
| 538.9478 | 0.24167 | 538.9478 | 0.62399 |
| 539.912 | 0.29565 | 539.912 | 0.69403 |
| 540.8763 | 0.3459 | 540.8763 | 0.78284 |
| 541.8405 | 0.36346 | 541.8405 | 0.85427 |
| 542.8047 | 0.32892 | 542.8047 | 0.89662 |
| 543.769 | 0.25366 | 543.769 | 0.91269 |
| 544.7332 | 0.16772 | 544.7332 | 0.90695 |
| 545.6974 | 0.10733 | 545.6974 | 0.87738 |
| 546.6617 | 0.07705 | 546.6617 | 0.84768 |
| 547.6259 | 0.08899 | 547.6259 | 0.85423 |
| 548.5901 | 0.13819 | 548.5901 | 0.89156 |
| 549.5544 | 0.19848 | 549.5544 | 0.94032 |
| 550.5186 | 0.23545 | 550.5186 | 0.97212 |
| 551.4828 | 0.2325 | 551.4828 | 0.97526 |
| 552.447 | 0.20364 | 552.447 | 0.96932 |
| 553.4113 | 0.15415 | 553.4113 | 0.96358 |
| 554.3755 | 0.09032 | 554.3755 | 0.95261 |
| 555.3397 | 0.02267 | 555.3397 | 0.92313 |
| 556.304 | -0.04357 | 556.304 | 0.86863 |
| 557.2682 | -0.10908 | 557.2682 | 0.80439 |
| 558.2324 | -0.18028 | 558.2324 | 0.75893 |
| 559.1967 | -0.26007 | 559.1967 | 0.73962 |
| 560.1609 | -0.34483 | 560.1609 | 0.72075 |
| 561.1251 | -0.43182 | 561.1251 | 0.68077 |
| 562.0894 | -0.51485 | 562.0894 | 0.61496 |
| 563.0536 | -0.57543 | 563.0536 | 0.53022 |
| 564.0178 | -0.60248 | 564.0178 | 0.45686 |
| 564.9821 | -0.61489 | 564.9821 | 0.38994 |
| 565.9463 | -0.61417 | 565.9463 | 0.31492 |
| 566.9105 | -0.60916 | 566.9105 | 0.23511 |
| 567.8748 | -0.609 | 567.8748 | 0.16109 |
| 568.839 | -0.61877 | 568.839 | 0.10782 |
| 569.8032 | -0.61893 | 569.8032 | 0.08488 |
| 570.7675 | -0.59969 | 570.7675 | 0.06952 |
| 571.7317 | -0.57075 | 571.7317 | 0.05334 |
| 572.6959 | -0.55741 | 572.6959 | 0.02171 |
| 573.6602 | -0.57966 | 573.6602 | -0.03676 |
| 574.6244 | -0.58203 | 574.6244 | -0.08213 |
| 575.5886 | -0.49379 | 575.5886 | -0.07021 |
| 576.5529 | -0.32603 | 576.5529 | 3.64E-04 |
| 577.5171 | -0.15855 | 577.5171 | 0.10608 |
| 578.4813 | -0.07129 | 578.4813 | 0.21975 |
| 579.4456 | -0.05054 | 579.4456 | 0.32992 |
| 580.4098 | -0.03662 | 580.4098 | 0.41918 |
| 581.374 | -0.01747 | 581.374 | 0.47172 |
| 582.3383 | 0.00987 | 582.3383 | 0.47883 |
| 583.3025 | 0.05375 | 583.3025 | 0.42871 |
| 584.2667 | 0.10436 | 584.2667 | 0.34304 |
| 585.231 | 0.14152 | 585.231 | 0.27486 |
| 586.1952 | 0.14912 | 586.1952 | 0.24783 |
| 587.1594 | 0.1278 | 587.1594 | 0.23008 |
| 588.1237 | 0.10928 | 588.1237 | 0.19306 |
| 589.0879 | 0.08849 | 589.0879 | 0.13367 |
| 590.0521 | 0.04987 | 590.0521 | 0.0656 |
| 591.0164 | -0.00733 | 591.0164 | 0.01065 |
| 591.9806 | -0.08525 | 591.9806 | -0.03212 |
| 592.9448 | -0.15755 | 592.9448 | -0.05001 |
| 593.9091 | -0.20554 | 593.9091 | -0.03606 |
| 594.8733 | -0.23555 | 594.8733 | 0.00136 |
| 595.8375 | -0.25272 | 595.8375 | 0.04621 |
| 596.8018 | -0.26734 | 596.8018 | 0.0902 |
| 597.766 | -0.29236 | 597.766 | 0.12092 |
| 598.7302 | -0.3399 | 598.7302 | 0.11568 |
| 599.6945 | -0.39516 | 599.6945 | 0.08643 |
| 600.6587 | -0.4499 | 600.6587 | 0.03003 |
| 601.6229 | -0.49127 | 601.6229 | -0.03008 |
| 602.5872 | -0.49788 | 602.5872 | -0.05182 |
| 603.5514 | -0.4601 | 603.5514 | -0.00334 |
| 604.5156 | -0.40106 | 604.5156 | 0.09075 |
| 605.4799 | -0.34757 | 605.4799 | 0.17041 |
| 606.4441 | -0.31108 | 606.4441 | 0.22516 |
| 607.4083 | -0.298 | 607.4083 | 0.24344 |
| 608.3726 | -0.30291 | 608.3726 | 0.22069 |
| 609.3368 | -0.30113 | 609.3368 | 0.19053 |
| 610.301 | -0.25451 | 610.301 | 0.18811 |
| 611.2653 | -0.15497 | 611.2653 | 0.22076 |
| 612.2295 | -0.03484 | 612.2295 | 0.26184 |
| 613.1937 | 0.07113 | 613.1937 | 0.27522 |
| 614.158 | 0.1458 | 614.158 | 0.26546 |
| 615.1222 | 0.16332 | 615.1222 | 0.25037 |
| 616.0864 | 0.11528 | 616.0864 | 0.22725 |
| 617.0507 | 0.05499 | 617.0507 | 0.19701 |
| 618.0149 | 0.0578 | 618.0149 | 0.16657 |
| 618.9791 | 0.10758 | 618.9791 | 0.13345 |
| 619.9434 | 0.15815 | 619.9434 | 0.10945 |
| 620.9076 | 0.17564 | 620.9076 | 0.08615 |
| 621.8718 | 0.17654 | 621.8718 | 0.07552 |
| 622.8361 | 0.1999 | 622.8361 | 0.10442 |
| 623.8003 | 0.23573 | 623.8003 | 0.16128 |
| 624.7645 | 0.25667 | 624.7645 | 0.22963 |
| 625.7288 | 0.24781 | 625.7288 | 0.29134 |
| 626.693 | 0.22695 | 626.693 | 0.32779 |
| 627.6572 | 0.221 | 627.6572 | 0.31805 |
| 628.6215 | 0.23656 | 628.6215 | 0.26106 |
| 629.5857 | 0.24481 | 629.5857 | 0.18472 |
| 630.5499 | 0.22975 | 630.5499 | 0.11497 |
| 631.5142 | 0.20337 | 631.5142 | 0.05886 |
| 632.4784 | 0.18164 | 632.4784 | 0.02919 |
| 633.4426 | 0.18824 | 633.4426 | 0.02098 |
| 634.4069 | 0.20992 | 634.4069 | 0.02149 |
| 635.3711 | 0.23645 | 635.3711 | 0.01772 |
| 636.3353 | 0.25314 | 636.3353 | 0.00437 |
| 637.2996 | 0.20846 | 637.2996 | -0.01527 |
| 638.2638 | 0.10409 | 638.2638 | -0.03782 |
| 639.228 | -0.00615 | 639.228 | -0.04934 |
| 640.1923 | -0.06368 | 640.1923 | -0.0415 |
| 641.1565 | -0.06742 | 641.1565 | -0.01778 |
| 642.1207 | -0.0639 | 642.1207 | -0.00771 |
| 643.085 | -0.08528 | 643.085 | -0.02748 |
| 644.0492 | -0.1286 | 644.0492 | -0.07255 |
| 645.0134 | -0.18633 | 645.0134 | -0.13346 |
| 645.9777 | -0.24538 | 645.9777 | -0.19752 |
| 646.9419 | -0.2751 | 646.9419 | -0.24033 |
| 647.9061 | -0.25302 | 647.9061 | -0.23471 |
| 648.8704 | -0.20544 | 648.8704 | -0.20167 |
| 649.8346 | -0.16026 | 649.8346 | -0.14981 |
| 650.7988 | -0.13568 | 650.7988 | -0.1009 |
| 651.7631 | -0.11735 | 651.7631 | -0.07145 |
| 652.7273 | -0.06813 | 652.7273 | -0.0676 |
| 653.6915 | -0.02233 | 653.6915 | -0.1011 |
| 654.6558 | -0.01611 | 654.6558 | -0.14503 |
| 655.62 | -0.05554 | 655.62 | -0.19612 |
| 656.5842 | -0.10494 | 656.5842 | -0.24021 |
| 657.5485 | -0.12697 | 657.5485 | -0.26043 |
| 658.5127 | -0.13031 | 658.5127 | -0.26903 |
| 659.4769 | -0.15781 | 659.4769 | -0.28933 |
| 660.4412 | -0.22131 | 660.4412 | -0.32746 |
| 661.4054 | -0.29426 | 661.4054 | -0.37468 |
| 662.3696 | -0.34905 | 662.3696 | -0.42023 |
| 663.3339 | -0.36996 | 663.3339 | -0.45701 |
| 664.2981 | -0.35299 | 664.2981 | -0.48314 |
| 665.2623 | -0.28261 | 665.2623 | -0.48952 |
| 666.2266 | -0.18854 | 666.2266 | -0.47913 |
| 667.1908 | -0.13891 | 667.1908 | -0.47117 |
| 668.155 | -0.13374 | 668.155 | -0.46033 |
| 669.1193 | -0.13474 | 669.1193 | -0.42478 |
| 670.0835 | -0.10181 | 670.0835 | -0.33918 |
| 671.0477 | -0.05148 | 671.0477 | -0.23513 |
| 672.012 | -0.03996 | 672.012 | -0.17797 |
| 672.9762 | -0.06669 | 672.9762 | -0.17194 |
| 673.9404 | -0.10628 | 673.9404 | -0.20609 |
| 674.9047 | -0.13719 | 674.9047 | -0.26511 |
| 675.8689 | -0.16205 | 675.8689 | -0.32091 |
| 676.8331 | -0.20458 | 676.8331 | -0.30976 |
| 677.7974 | -0.25952 | 677.7974 | -0.23765 |
| 678.7616 | -0.28274 | 678.7616 | -0.15835 |
| 679.7258 | -0.24734 | 679.7258 | -0.1079 |
| 680.6901 | -0.1751 | 680.6901 | -0.0877 |
| 681.6543 | -0.12859 | 681.6543 | -0.07383 |
| 682.6185 | -0.15518 | 682.6185 | -0.04773 |
| 683.5828 | -0.20623 | 683.5828 | -0.0128 |
| 684.547 | -0.23737 | 684.547 | 0.01828 |
| 685.5112 | -0.24722 | 685.5112 | 0.04652 |
| 686.4755 | -0.24545 | 686.4755 | 0.07615 |
| 687.4397 | -0.24116 | 687.4397 | 0.11093 |
| 688.4039 | -0.23227 | 688.4039 | 0.13374 |
| 689.3682 | -0.22343 | 689.3682 | 0.11707 |
| 690.3324 | -0.22318 | 690.3324 | 0.07793 |
| 691.2966 | -0.2335 | 691.2966 | 0.04803 |
| 692.2609 | -0.25452 | 692.2609 | 0.0415 |
| 693.2251 | -0.28198 | 693.2251 | 0.04822 |
| 694.1893 | -0.31122 | 694.1893 | 0.04481 |
| 695.1536 | -0.34864 | 695.1536 | 0.01961 |
| 696.1178 | -0.42395 | 696.1178 | -0.03386 |
| 697.082 | -0.54656 | 697.082 | -0.11591 |
| 698.0463 | -0.68997 | 698.0463 | -0.20652 |
| 699.0105 | -0.80991 | 699.0105 | -0.27393 |
| 699.9747 | -0.8766 | 699.9747 | -0.29433 |
| 700.939 | -0.91574 | 700.939 | -0.28158 |
| 701.9032 | -0.99491 | 701.9032 | -0.27995 |
| 702.8674 | -1.08623 | 702.8674 | -0.28557 |
| 703.8317 | -1.13282 | 703.8317 | -0.27916 |
| 704.7959 | -1.11313 | 704.7959 | -0.25139 |
| 705.7601 | -1.05993 | 705.7601 | -0.21058 |
| 706.7244 | -1.02772 | 706.7244 | -0.17676 |
| 707.6886 | -1.00566 | 707.6886 | -0.15405 |
| 708.6528 | -0.9555 | 708.6528 | -0.12229 |
| 709.6171 | -0.87557 | 709.6171 | -0.08757 |
| 710.5813 | -0.79395 | 710.5813 | -0.08085 |
| 711.5455 | -0.74098 | 711.5455 | -0.12704 |
| 712.5098 | -0.71393 | 712.5098 | -0.20053 |
| 713.474 | -0.66949 | 713.474 | -0.22421 |
| 714.4382 | -0.60283 | 714.4382 | -0.18762 |
| 715.4025 | -0.53874 | 715.4025 | -0.12467 |
| 716.3667 | -0.49695 | 716.3667 | -0.06722 |
| 717.3309 | -0.4823 | 717.3309 | -0.03239 |
| 718.2952 | -0.48699 | 718.2952 | -0.02476 |
| 719.2594 | -0.50604 | 719.2594 | -0.05453 |
| 720.2236 | -0.52245 | 720.2236 | -0.09248 |
| 721.1879 | -0.50931 | 721.1879 | -0.09492 |
| 722.1521 | -0.47087 | 722.1521 | -0.05737 |
| 723.1163 | -0.43624 | 723.1163 | 0.00565 |
| 724.0806 | -0.43004 | 724.0806 | 0.08116 |
| 725.0448 | -0.42986 | 725.0448 | 0.16375 |
| 726.009 | -0.37037 | 726.009 | 0.22069 |
| 726.9733 | -0.28117 | 726.9733 | 0.23718 |
| 727.9375 | -0.23192 | 727.9375 | 0.22108 |
| 728.9017 | -0.247 | 728.9017 | 0.19812 |
| 729.866 | -0.2894 | 729.866 | 0.19564 |
| 730.8302 | -0.28593 | 730.8302 | 0.22607 |
| 731.7944 | -0.21326 | 731.7944 | 0.27489 |
| 732.7587 | -0.1397 | 732.7587 | 0.303 |
| 733.7229 | -0.11203 | 733.7229 | 0.2976 |
| 734.6871 | -0.13172 | 734.6871 | 0.26855 |
| 735.6514 | -0.17195 | 735.6514 | 0.22554 |
| 736.6156 | -0.20522 | 736.6156 | 0.16714 |
| 737.5798 | -0.23454 | 737.5798 | 0.10427 |
| 738.5441 | -0.27567 | 738.5441 | 0.07525 |
| 739.5083 | -0.33196 | 739.5083 | 0.06476 |
| 740.4725 | -0.40074 | 740.4725 | 0.0446 |
| 741.4368 | -0.47384 | 741.4368 | 0.00344 |
| 742.401 | -0.54385 | 742.401 | -0.0514 |
| 743.3652 | -0.60849 | 743.3652 | -0.10767 |
| 744.3295 | -0.67218 | 744.3295 | -0.16418 |
| 745.2937 | -0.7373 | 745.2937 | -0.23254 |
| 746.2579 | -0.79554 | 746.2579 | -0.30249 |
| 747.2222 | -0.84589 | 747.2222 | -0.35563 |
| 748.1864 | -0.89039 | 748.1864 | -0.38285 |
| 749.1506 | -0.92504 | 749.1506 | -0.38844 |
| 750.1149 | -0.93397 | 750.1149 | -0.38487 |
| 751.0791 | -0.91299 | 751.0791 | -0.38387 |
| 752.0433 | -0.8886 | 752.0433 | -0.39103 |
| 753.0076 | -0.88897 | 753.0076 | -0.40415 |
| 753.9718 | -0.92047 | 753.9718 | -0.41682 |
| 754.936 | -0.96192 | 754.936 | -0.42765 |
| 755.9003 | -0.9789 | 755.9003 | -0.44138 |
| 756.8645 | -0.97615 | 756.8645 | -0.45645 |
| 757.8287 | -0.97047 | 757.8287 | -0.47413 |
| 758.793 | -0.96185 | 758.793 | -0.49838 |
| 759.7572 | -0.93006 | 759.7572 | -0.51107 |
| 760.7214 | -0.85939 | 760.7214 | -0.47762 |
| 761.6857 | -0.76496 | 761.6857 | -0.39695 |
| 762.6499 | -0.69653 | 762.6499 | -0.32802 |
| 763.6141 | -0.65765 | 763.6141 | -0.28878 |
| 764.5784 | -0.61164 | 764.5784 | -0.25786 |
| 765.5426 | -0.54418 | 765.5426 | -0.22162 |
| 766.5068 | -0.46269 | 766.5068 | -0.18307 |
| 767.4711 | -0.3837 | 767.4711 | -0.1488 |
| 768.4353 | -0.31516 | 768.4353 | -0.11144 |
| 769.3995 | -0.26094 | 769.3995 | -0.06216 |
| 770.3638 | -0.22908 | 770.3638 | -0.00434 |
| 771.328 | -0.2143 | 771.328 | 0.04236 |
| 772.2922 | -0.19594 | 772.2922 | 0.07266 |
| 773.2565 | -0.15062 | 773.2565 | 0.09682 |
| 774.2207 | -0.08423 | 774.2207 | 0.11735 |
| 775.1849 | -0.04552 | 775.1849 | 0.11644 |
| 776.1492 | -0.03475 | 776.1492 | 0.09486 |
| 777.1134 | -0.02237 | 777.1134 | 0.06796 |
| 778.0776 | 0.02159 | 778.0776 | 0.05131 |
| 779.0419 | 0.08888 | 779.0419 | 0.03811 |
| 780.0061 | 0.13667 | 780.0061 | 0.00495 |
| 780.9703 | 0.14013 | 780.9703 | -0.05413 |
| 781.9346 | 0.11485 | 781.9346 | -0.10784 |
| 782.8988 | 0.08523 | 782.8988 | -0.13447 |
| 783.863 | 0.0884 | 783.863 | -0.12517 |
| 784.8273 | 0.12297 | 784.8273 | -0.09671 |
| 785.7915 | 0.159 | 785.7915 | -0.07256 |
| 786.7557 | 0.16733 | 786.7557 | -0.06651 |
| 787.72 | 0.13784 | 787.72 | -0.09225 |
| 788.6842 | 0.10129 | 788.6842 | -0.1451 |
| 789.6484 | 0.09378 | 789.6484 | -0.21145 |
| 790.6127 | 0.08608 | 790.6127 | -0.2872 |
| 791.5769 | 0.03428 | 791.5769 | -0.38236 |
| 792.5411 | -0.0693 | 792.5411 | -0.48821 |
| 793.5054 | -0.19893 | 793.5054 | -0.57855 |
| 794.4696 | -0.31873 | 794.4696 | -0.61809 |
| 795.4338 | -0.41584 | 795.4338 | -0.61339 |
| 796.3981 | -0.47445 | 796.3981 | -0.58976 |
| 797.3623 | -0.49833 | 797.3623 | -0.5642 |
| 798.3265 | -0.49657 | 798.3265 | -0.53864 |
| 799.2908 | -0.49068 | 799.2908 | -0.51357 |
| 800.255 | -0.50234 | 800.255 | -0.49794 |
| 801.2192 | -0.5343 | 801.2192 | -0.49982 |
| 802.1835 | -0.57535 | 802.1835 | -0.51085 |
| 803.1477 | -0.6013 | 803.1477 | -0.49647 |
| 804.1119 | -0.60502 | 804.1119 | -0.44813 |
| 805.0762 | -0.58826 | 805.0762 | -0.3841 |
| 806.0404 | -0.55162 | 806.0404 | -0.3252 |
| 807.0046 | -0.50208 | 807.0046 | -0.2946 |
| 807.9689 | -0.45843 | 807.9689 | -0.2861 |
| 808.9331 | -0.449 | 808.9331 | -0.28683 |
| 809.8973 | -0.46532 | 809.8973 | -0.27867 |
| 810.8616 | -0.48774 | 810.8616 | -0.25097 |
| 811.8258 | -0.50042 | 811.8258 | -0.21393 |
| 812.79 | -0.50685 | 812.79 | -0.1805 |
| 813.7543 | -0.53083 | 813.7543 | -0.16783 |
| 814.7185 | -0.57677 | 814.7185 | -0.17912 |
| 815.6827 | -0.63223 | 815.6827 | -0.20743 |
| 816.647 | -0.68613 | 816.647 | -0.2338 |
| 817.6112 | -0.73793 | 817.6112 | -0.24631 |
| 818.5754 | -0.7889 | 818.5754 | -0.25765 |
| 819.5397 | -0.83528 | 819.5397 | -0.28437 |
| 820.5039 | -0.87022 | 820.5039 | -0.31541 |
| 821.4681 | -0.88695 | 821.4681 | -0.33682 |
| 822.4324 | -0.87978 | 822.4324 | -0.34843 |
| 823.3966 | -0.85324 | 823.3966 | -0.36083 |
| 824.3608 | -0.83933 | 824.3608 | -0.38519 |
| 825.3251 | -0.85459 | 825.3251 | -0.42202 |
| 826.2893 | -0.88096 | 826.2893 | -0.45545 |
| 827.2535 | -0.90256 | 827.2535 | -0.4697 |
| 828.2178 | -0.91091 | 828.2178 | -0.44877 |
| 829.182 | -0.91226 | 829.182 | -0.40746 |
| 830.1462 | -0.89888 | 830.1462 | -0.36194 |
| 831.1105 | -0.85897 | 831.1105 | -0.31303 |
| 832.0747 | -0.80648 | 832.0747 | -0.26927 |
| 833.0389 | -0.78336 | 833.0389 | -0.25071 |
| 834.0032 | -0.7937 | 834.0032 | -0.26054 |
| 834.9674 | -0.80084 | 834.9674 | -0.28798 |
| 835.9316 | -0.78074 | 835.9316 | -0.32503 |
| 836.8959 | -0.75541 | 836.8959 | -0.37242 |
| 837.8601 | -0.76299 | 837.8601 | -0.42837 |
| 838.8243 | -0.83209 | 838.8243 | -0.48716 |
| 839.7886 | -0.92572 | 839.7886 | -0.539 |
| 840.7528 | -0.98925 | 840.7528 | -0.55952 |
| 841.717 | -0.99864 | 841.717 | -0.53002 |
| 842.6813 | -0.94167 | 842.6813 | -0.43474 |
| 843.6455 | -0.85293 | 843.6455 | -0.32883 |
| 844.6097 | -0.76261 | 844.6097 | -0.25655 |
| 845.574 | -0.69785 | 845.574 | -0.22129 |
| 846.5382 | -0.66819 | 846.5382 | -0.21632 |
| 847.5024 | -0.68099 | 847.5024 | -0.23931 |
| 848.4667 | -0.72652 | 848.4667 | -0.27479 |
| 849.4309 | -0.78013 | 849.4309 | -0.27895 |
| 850.3951 | -0.81443 | 850.3951 | -0.23932 |
| 851.3594 | -0.81692 | 851.3594 | -0.18034 |
| 852.3236 | -0.8002 | 852.3236 | -0.14606 |
| 853.2878 | -0.76988 | 853.2878 | -0.14417 |
| 854.2521 | -0.72649 | 854.2521 | -0.14708 |
| 855.2163 | -0.67618 | 855.2163 | -0.13084 |
| 856.1805 | -0.62755 | 856.1805 | -0.09329 |
| 857.1448 | -0.57565 | 857.1448 | -0.03876 |
| 858.109 | -0.51902 | 858.109 | 0.02181 |
| 859.0732 | -0.49245 | 859.0732 | 0.05585 |
| 860.0375 | -0.50587 | 860.0375 | 0.0483 |
| 861.0017 | -0.52623 | 861.0017 | 0.03109 |
| 861.9659 | -0.51457 | 861.9659 | 0.04759 |
| 862.9302 | -0.49154 | 862.9302 | 0.07922 |
| 863.8944 | -0.49848 | 863.8944 | 0.08725 |
| 864.8586 | -0.52712 | 864.8586 | 0.08837 |
| 865.8229 | -0.5413 | 865.8229 | 0.10555 |
| 866.7871 | -0.51738 | 866.7871 | 0.14273 |
| 867.7513 | -0.47058 | 867.7513 | 0.18706 |
| 868.7156 | -0.4389 | 868.7156 | 0.22084 |
| 869.6798 | -0.42942 | 869.6798 | 0.23736 |
| 870.644 | -0.40464 | 870.644 | 0.23803 |
| 871.6083 | -0.362 | 871.6083 | 0.22755 |
| 872.5725 | -0.3365 | 872.5725 | 0.21427 |
| 873.5367 | -0.34466 | 873.5367 | 0.20673 |
| 874.501 | -0.37042 | 874.501 | 0.21215 |
| 875.4652 | -0.38288 | 875.4652 | 0.22075 |
| 876.4294 | -0.36963 | 876.4294 | 0.20531 |
| 877.3937 | -0.36218 | 877.3937 | 0.15191 |
| 878.3579 | -0.37671 | 878.3579 | 0.07823 |
| 879.3221 | -0.41797 | 879.3221 | -3.84E-04 |
| 880.2864 | -0.47663 | 880.2864 | -0.0829 |
| 881.2505 | -0.53773 | 881.2505 | -0.16385 |
| 882.2148 | -0.60477 | 882.2148 | -0.23246 |
| 883.179 | -0.71176 | 883.179 | -0.28093 |
| 884.1432 | -0.86132 | 884.1432 | -0.31778 |
| 885.1075 | -1.00956 | 885.1075 | -0.34461 |
| 886.0717 | -1.11258 | 886.0717 | -0.36313 |
| 887.0359 | -1.16992 | 887.0359 | -0.3932 |
| 888.0002 | -1.20548 | 888.0002 | -0.45266 |
| 888.9644 | -1.22837 | 888.9644 | -0.52981 |
| 889.9286 | -1.21516 | 889.9286 | -0.57544 |
| 890.8929 | -1.15234 | 890.8929 | -0.56397 |
| 891.8571 | -1.10736 | 891.8571 | -0.57625 |
| 892.8214 | -1.10459 | 892.8214 | -0.62561 |
| 893.7856 | -1.13733 | 893.7856 | -0.68371 |
| 894.7498 | -1.19599 | 894.7498 | -0.73961 |
| 895.7141 | -1.26901 | 895.7141 | -0.79255 |
| 896.6783 | -1.32699 | 896.6783 | -0.82263 |
| 897.6425 | -1.34248 | 897.6425 | -0.8034 |
| 898.6068 | -1.32365 | 898.6068 | -0.78704 |
| 899.571 | -1.28079 | 899.571 | -0.81544 |
| 900.5352 | -1.21966 | 900.5352 | -0.86103 |
| 901.4995 | -1.14694 | 901.4995 | -0.86735 |
| 902.4637 | -1.07352 | 902.4637 | -0.80033 |
| 903.4279 | -1.01221 | 903.4279 | -0.69003 |
| 904.3922 | -0.9729 | 904.3922 | -0.61105 |
| 905.3564 | -0.9545 | 905.3564 | -0.58996 |
| 906.3206 | -0.94557 | 906.3206 | -0.60272 |
| 907.2849 | -0.91946 | 907.2849 | -0.61282 |
| 908.2491 | -0.85235 | 908.2491 | -0.58296 |
| 909.2133 | -0.76004 | 909.2133 | -0.50578 |
| 910.1776 | -0.69087 | 910.1776 | -0.41201 |
| 911.1418 | -0.65658 | 911.1418 | -0.35029 |
| 912.106 | -0.58335 | 912.106 | -0.30858 |
| 913.0703 | -0.458 | 913.0703 | -0.24788 |
| 914.0345 | -0.32028 | 914.0345 | -0.17051 |
| 914.9987 | -0.21845 | 914.9987 | -0.09933 |
| 915.963 | -0.17926 | 915.963 | -0.05469 |
| 916.9272 | -0.19067 | 916.9272 | -0.03952 |
| 917.8914 | -0.19281 | 917.8914 | -0.03645 |
| 918.8557 | -0.12042 | 918.8557 | -0.02222 |
| 919.8199 | -0.01772 | 919.8199 | 0.00318 |
| 920.7841 | 0.04059 | 920.7841 | 0.02453 |
| 921.7484 | 0.03482 | 921.7484 | 0.02864 |
| 922.7126 | 0.0053 | 922.7126 | 0.01468 |
| 923.6768 | 0.00756 | 923.6768 | -0.00503 |
| 924.6411 | 0.05016 | 924.6411 | -0.01454 |
| 925.6053 | 0.07802 | 925.6053 | -0.01583 |
| 926.5695 | 0.06716 | 926.5695 | -0.04052 |
| 927.5338 | 0.03263 | 927.5338 | -0.07655 |
| 928.498 | -0.02044 | 928.498 | -0.09194 |
| 929.4622 | -0.0914 | 929.4622 | -0.07467 |
| 930.4265 | -0.17601 | 930.4265 | -0.03746 |
| 931.3907 | -0.25428 | 931.3907 | -6.56E-04 |
| 932.3549 | -0.27389 | 932.3549 | 0.02921 |
| 933.3192 | -0.20172 | 933.3192 | 0.07227 |
| 934.2834 | -0.10259 | 934.2834 | 0.10818 |
| 935.2476 | -0.04956 | 935.2476 | 0.10625 |
| 936.2119 | -0.05058 | 936.2119 | 0.07953 |
| 937.1761 | -0.05732 | 937.1761 | 0.07368 |
| 938.1403 | -0.02512 | 938.1403 | 0.11962 |
| 939.1046 | 0.02022 | 939.1046 | 0.18456 |
| 940.0688 | -0.00643 | 940.0688 | 0.21134 |
| 941.033 | -0.11662 | 941.033 | 0.21615 |
| 941.9973 | -0.25771 | 941.9973 | 0.20463 |
| 942.9615 | -0.38818 | 942.9615 | 0.16496 |
| 943.9257 | -0.48754 | 943.9257 | 0.11164 |
| 944.89 | -0.54797 | 944.89 | 0.08565 |
| 945.8542 | -0.58084 | 945.8542 | 0.10063 |
| 946.8184 | -0.60755 | 946.8184 | 0.10558 |
| 947.7827 | -0.6125 | 947.7827 | 0.0951 |
| 948.7469 | -0.60933 | 948.7469 | 0.10681 |
| 949.7111 | -0.62619 | 949.7111 | 0.14742 |
| 950.6754 | -0.66636 | 950.6754 | 0.18475 |
| 951.6396 | -0.70762 | 951.6396 | 0.17615 |
| 952.6038 | -0.72583 | 952.6038 | 0.11731 |
| 953.5681 | -0.71947 | 953.5681 | 0.05559 |
| 954.5323 | -0.71278 | 954.5323 | 0.01111 |
| 955.4965 | -0.73126 | 955.4965 | -0.02926 |
| 956.4608 | -0.78748 | 956.4608 | -0.05887 |
| 957.425 | -0.87213 | 957.425 | -0.06687 |
| 958.3892 | -0.94233 | 958.3892 | -0.05804 |
| 959.3535 | -0.94555 | 959.3535 | -0.05273 |
| 960.3177 | -0.89083 | 960.3177 | -0.06576 |
| 961.2819 | -0.86711 | 961.2819 | -0.09305 |
| 962.2462 | -0.88895 | 962.2462 | -0.1175 |
| 963.2104 | -0.91965 | 963.2104 | -0.14752 |
| 964.1746 | -0.93282 | 964.1746 | -0.20065 |
| 965.1389 | -0.91367 | 965.1389 | -0.26794 |
| 966.1031 | -0.85031 | 966.1031 | -0.32037 |
| 967.0673 | -0.75445 | 967.0673 | -0.3417 |
| 968.0316 | -0.68751 | 968.0316 | -0.35472 |
| 968.9958 | -0.66871 | 968.9958 | -0.38118 |
| 969.96 | -0.65833 | 969.96 | -0.41466 |
| 970.9243 | -0.62078 | 970.9243 | -0.44377 |
| 971.8885 | -0.55635 | 971.8885 | -0.46626 |
| 972.8527 | -0.49571 | 972.8527 | -0.48657 |
| 973.817 | -0.46354 | 973.817 | -0.50425 |
| 974.7812 | -0.43685 | 974.7812 | -0.49285 |
| 975.7454 | -0.38149 | 975.7454 | -0.40676 |
| 976.7097 | -0.28442 | 976.7097 | -0.242 |
| 977.6739 | -0.16752 | 977.6739 | -0.05876 |
| 978.6381 | -0.06413 | 978.6381 | 0.1012 |
| 979.6024 | 0.02474 | 979.6024 | 0.23517 |
| 980.5666 | 0.11161 | 980.5666 | 0.34714 |
| 981.5308 | 0.19448 | 981.5308 | 0.42916 |
| 982.4951 | 0.22934 | 982.4951 | 0.46242 |
| 983.4593 | 0.22237 | 983.4593 | 0.46588 |
| 984.4235 | 0.21575 | 984.4235 | 0.47525 |
| 985.3878 | 0.23083 | 985.3878 | 0.51003 |
| 986.352 | 0.25524 | 986.352 | 0.56671 |
| 987.3162 | 0.255 | 987.3162 | 0.62579 |
| 988.2805 | 0.2159 | 988.2805 | 0.67173 |
| 989.2447 | 0.17807 | 989.2447 | 0.69686 |
| 990.2089 | 0.18394 | 990.2089 | 0.69497 |
| 991.1732 | 0.2267 | 991.1732 | 0.68343 |
| 992.1374 | 0.29239 | 992.1374 | 0.69786 |
| 993.1016 | 0.37866 | 993.1016 | 0.75354 |
| 994.0659 | 0.47286 | 994.0659 | 0.81608 |
| 995.0301 | 0.5516 | 995.0301 | 0.8297 |
| 995.9943 | 0.60176 | 995.9943 | 0.79486 |
| 996.9586 | 0.61649 | 996.9586 | 0.75395 |
| 997.9228 | 0.6233 | 997.9228 | 0.74697 |
| 998.887 | 0.64825 | 998.887 | 0.76901 |
| 999.8513 | 0.69772 | 999.8513 | 0.79786 |
| 1000.815 | 0.75041 | 1000.815 | 0.82009 |
| 1001.78 | 0.76892 | 1001.78 | 0.82086 |
| 1002.744 | 0.74381 | 1002.744 | 0.8005 |
| 1003.708 | 0.70706 | 1003.708 | 0.78654 |
| 1004.672 | 0.69183 | 1004.672 | 0.80021 |
| 1005.637 | 0.7043 | 1005.637 | 0.82223 |
| 1006.601 | 0.71843 | 1006.601 | 0.80738 |
| 1007.565 | 0.70653 | 1007.565 | 0.7364 |
| 1008.529 | 0.68136 | 1008.529 | 0.64598 |
| 1009.494 | 0.66146 | 1009.494 | 0.57906 |
| 1010.458 | 0.66018 | 1010.458 | 0.53081 |
| 1011.422 | 0.68273 | 1011.422 | 0.45829 |
| 1012.386 | 0.71069 | 1012.386 | 0.35136 |
| 1013.351 | 0.73964 | 1013.351 | 0.2596 |
| 1014.315 | 0.77071 | 1014.315 | 0.22456 |
| 1015.279 | 0.79698 | 1015.279 | 0.22641 |
| 1016.243 | 0.80945 | 1016.243 | 0.23686 |
| 1017.207 | 0.80932 | 1017.207 | 0.24832 |
| 1018.172 | 0.80287 | 1018.172 | 0.27019 |
| 1019.136 | 0.79751 | 1019.136 | 0.30669 |
| 1020.1 | 0.81134 | 1020.1 | 0.35888 |
| 1021.064 | 0.87065 | 1021.064 | 0.42403 |
| 1022.029 | 0.97455 | 1022.029 | 0.49715 |
| 1022.993 | 1.07319 | 1022.993 | 0.5777 |
| 1023.957 | 1.12802 | 1023.957 | 0.65974 |
| 1024.921 | 1.13355 | 1024.921 | 0.72587 |
| 1025.886 | 1.08522 | 1025.886 | 0.74492 |
| 1026.85 | 1.00991 | 1026.85 | 0.72548 |
| 1027.814 | 0.94497 | 1027.814 | 0.67868 |
| 1028.778 | 0.90115 | 1028.778 | 0.60657 |
| 1029.743 | 0.8696 | 1029.743 | 0.52672 |
| 1030.707 | 0.83369 | 1030.707 | 0.45125 |
| 1031.671 | 0.79947 | 1031.671 | 0.38385 |
| 1032.635 | 0.78744 | 1032.635 | 0.32795 |
| 1033.599 | 0.80263 | 1033.599 | 0.28325 |
| 1034.564 | 0.826 | 1034.564 | 0.23536 |
| 1035.528 | 0.82728 | 1035.528 | 0.16565 |
| 1036.492 | 0.80674 | 1036.492 | 0.08223 |
| 1037.456 | 0.79614 | 1037.456 | 0.02489 |
| 1038.421 | 0.81362 | 1038.421 | 0.00964 |
| 1039.385 | 0.86481 | 1039.385 | 0.01585 |
| 1040.349 | 0.9501 | 1040.349 | 0.02446 |
| 1041.313 | 1.042 | 1041.313 | 0.03268 |
| 1042.278 | 1.09106 | 1042.278 | 0.04851 |
| 1043.242 | 1.0811 | 1043.242 | 0.08915 |
| 1044.206 | 1.05427 | 1044.206 | 0.15544 |
| 1045.17 | 1.04518 | 1045.17 | 0.21165 |
| 1046.134 | 1.04176 | 1046.134 | 0.25931 |
| 1047.099 | 1.02058 | 1047.099 | 0.29466 |
| 1048.063 | 0.98218 | 1048.063 | 0.32126 |
| 1049.027 | 0.93443 | 1049.027 | 0.34569 |
| 1049.991 | 0.88647 | 1049.991 | 0.36552 |
| 1050.956 | 0.85143 | 1050.956 | 0.38694 |
| 1051.92 | 0.83415 | 1051.92 | 0.41398 |
| 1052.884 | 0.82893 | 1052.884 | 0.43988 |
| 1053.848 | 0.82829 | 1053.848 | 0.44859 |
| 1054.813 | 0.82863 | 1054.813 | 0.43648 |
| 1055.777 | 0.85195 | 1055.777 | 0.43133 |
| 1056.741 | 0.92164 | 1056.741 | 0.46292 |
| 1057.705 | 1.01257 | 1057.705 | 0.52024 |
| 1058.669 | 1.08379 | 1058.669 | 0.57295 |
| 1059.634 | 1.11991 | 1059.634 | 0.61376 |
| 1060.598 | 1.14803 | 1060.598 | 0.67807 |
| 1061.562 | 1.20336 | 1061.562 | 0.81423 |
| 1062.526 | 1.2822 | 1062.526 | 0.9944 |
| 1063.491 | 1.38395 | 1063.491 | 1.16726 |
| 1064.455 | 1.4993 | 1064.455 | 1.3169 |
| 1065.419 | 1.60436 | 1065.419 | 1.43354 |
| 1066.383 | 1.67931 | 1066.383 | 1.51228 |
| 1067.348 | 1.74299 | 1067.348 | 1.55813 |
| 1068.312 | 1.83611 | 1068.312 | 1.57982 |
| 1069.276 | 1.95035 | 1069.276 | 1.58749 |
| 1070.24 | 2.0173 | 1070.24 | 1.59668 |
| 1071.204 | 2.00236 | 1071.204 | 1.61455 |
| 1072.169 | 1.95638 | 1072.169 | 1.62976 |
| 1073.133 | 1.93928 | 1073.133 | 1.62858 |
| 1074.097 | 1.95475 | 1074.097 | 1.60196 |
| 1075.061 | 1.95309 | 1075.061 | 1.54053 |
| 1076.026 | 1.88281 | 1076.026 | 1.43628 |
| 1076.99 | 1.79325 | 1076.99 | 1.31352 |
| 1077.954 | 1.75452 | 1077.954 | 1.20525 |
| 1078.918 | 1.75397 | 1078.918 | 1.11357 |
| 1079.883 | 1.76935 | 1079.883 | 1.03709 |
| 1080.847 | 1.78317 | 1080.847 | 0.97445 |
| 1081.811 | 1.77793 | 1081.811 | 0.92366 |
| 1082.775 | 1.75254 | 1082.775 | 0.88948 |
| 1083.74 | 1.72883 | 1083.74 | 0.87319 |
| 1084.704 | 1.74618 | 1084.704 | 0.86724 |
| 1085.668 | 1.8078 | 1085.668 | 0.87167 |
| 1086.632 | 1.86642 | 1086.632 | 0.87807 |
| 1087.596 | 1.88948 | 1087.596 | 0.87816 |
| 1088.561 | 1.86935 | 1088.561 | 0.87555 |
| 1089.525 | 1.80565 | 1089.525 | 0.87072 |
| 1090.489 | 1.71574 | 1090.489 | 0.85991 |
| 1091.453 | 1.63611 | 1091.453 | 0.8406 |
| 1092.418 | 1.60267 | 1092.418 | 0.81409 |
| 1093.382 | 1.60665 | 1093.382 | 0.79537 |
| 1094.346 | 1.63349 | 1094.346 | 0.83699 |
| 1095.31 | 1.67422 | 1095.31 | 0.91658 |
| 1096.275 | 1.71894 | 1096.275 | 0.98612 |
| 1097.239 | 1.76157 | 1097.239 | 1.03033 |
| 1098.203 | 1.79792 | 1098.203 | 1.05689 |
| 1099.167 | 1.82564 | 1099.167 | 1.07732 |
| 1100.131 | 1.85253 | 1100.131 | 1.09897 |
| 1101.096 | 1.89846 | 1101.096 | 1.13166 |
| 1102.06 | 1.96255 | 1102.06 | 1.17446 |
| 1103.024 | 2.01934 | 1103.024 | 1.21726 |
| 1103.988 | 2.05245 | 1103.988 | 1.25457 |
| 1104.953 | 2.06097 | 1104.953 | 1.2902 |
| 1105.917 | 2.05051 | 1105.917 | 1.3314 |
| 1106.881 | 2.0329 | 1106.881 | 1.37738 |
| 1107.845 | 2.03283 | 1107.845 | 1.41236 |
| 1108.81 | 2.07473 | 1108.81 | 1.41346 |
| 1109.774 | 2.15529 | 1109.774 | 1.40983 |
| 1110.738 | 2.26998 | 1110.738 | 1.43103 |
| 1111.702 | 2.39522 | 1111.702 | 1.46393 |
| 1112.667 | 2.48193 | 1112.667 | 1.4795 |
| 1113.631 | 2.51864 | 1113.631 | 1.46857 |
| 1114.595 | 2.54892 | 1114.595 | 1.45009 |
| 1115.559 | 2.62149 | 1115.559 | 1.44603 |
| 1116.523 | 2.71595 | 1116.523 | 1.44929 |
| 1117.488 | 2.75017 | 1117.488 | 1.43067 |
| 1118.452 | 2.70284 | 1118.452 | 1.38292 |
| 1119.416 | 2.63347 | 1119.416 | 1.31742 |
| 1120.38 | 2.58312 | 1120.38 | 1.24205 |
| 1121.345 | 2.54099 | 1121.345 | 1.15934 |
| 1122.309 | 2.47302 | 1122.309 | 1.07063 |
| 1123.273 | 2.37245 | 1123.273 | 0.98662 |
| 1124.237 | 2.28776 | 1124.237 | 0.93559 |
| 1125.202 | 2.28966 | 1125.202 | 0.95562 |
| 1126.166 | 2.34714 | 1126.166 | 1.01416 |
| 1127.13 | 2.38601 | 1127.13 | 1.06291 |
| 1128.094 | 2.40353 | 1128.094 | 1.09863 |
| 1129.058 | 2.42234 | 1129.058 | 1.129 |
| 1130.023 | 2.45409 | 1130.023 | 1.15634 |
| 1130.987 | 2.49061 | 1130.987 | 1.16765 |
| 1131.951 | 2.51822 | 1131.951 | 1.14647 |
| 1132.915 | 2.53472 | 1132.915 | 1.10539 |
| 1133.88 | 2.54357 | 1133.88 | 1.09153 |
| 1134.844 | 2.54474 | 1134.844 | 1.11981 |
| 1135.808 | 2.53595 | 1135.808 | 1.15841 |
| 1136.772 | 2.52338 | 1136.772 | 1.18413 |
| 1137.737 | 2.52115 | 1137.737 | 1.20282 |
| 1138.701 | 2.54016 | 1138.701 | 1.23554 |
| 1139.665 | 2.57871 | 1139.665 | 1.2926 |
| 1140.629 | 2.62326 | 1140.629 | 1.35722 |
| 1141.594 | 2.65902 | 1141.594 | 1.40307 |
| 1142.558 | 2.67792 | 1142.558 | 1.44153 |
| 1143.522 | 2.65686 | 1143.522 | 1.49698 |
| 1144.486 | 2.61189 | 1144.486 | 1.55477 |
| 1145.45 | 2.57694 | 1145.45 | 1.59906 |
| 1146.415 | 2.56108 | 1146.415 | 1.62588 |
| 1147.379 | 2.54916 | 1147.379 | 1.63065 |
| 1148.343 | 2.52882 | 1148.343 | 1.61293 |
| 1149.307 | 2.50299 | 1149.307 | 1.58921 |
| 1150.272 | 2.47388 | 1150.272 | 1.5837 |
| 1151.236 | 2.44413 | 1151.236 | 1.59502 |
| 1152.2 | 2.42065 | 1152.2 | 1.58246 |
| 1153.164 | 2.40981 | 1153.164 | 1.53082 |
| 1154.129 | 2.41072 | 1154.129 | 1.45515 |
| 1155.093 | 2.423 | 1155.093 | 1.36882 |
| 1156.057 | 2.45171 | 1156.057 | 1.28346 |
| 1157.021 | 2.49401 | 1157.021 | 1.23043 |
| 1157.985 | 2.54125 | 1157.985 | 1.25631 |
| 1158.95 | 2.59043 | 1158.95 | 1.31206 |
| 1159.914 | 2.62333 | 1159.914 | 1.29914 |
| 1160.878 | 2.63919 | 1160.878 | 1.23487 |
| 1161.842 | 2.65586 | 1161.842 | 1.16773 |
| 1162.807 | 2.68628 | 1162.807 | 1.12664 |
| 1163.771 | 2.7214 | 1163.771 | 1.11939 |
| 1164.735 | 2.73655 | 1164.735 | 1.13262 |
| 1165.699 | 2.72722 | 1165.699 | 1.15279 |
| 1166.664 | 2.7222 | 1166.664 | 1.18415 |
| 1167.628 | 2.7344 | 1167.628 | 1.223 |
| 1168.592 | 2.74526 | 1168.592 | 1.23977 |
| 1169.556 | 2.74208 | 1169.556 | 1.21965 |
| 1170.521 | 2.74157 | 1170.521 | 1.2024 |
| 1171.485 | 2.76809 | 1171.485 | 1.23393 |
| 1172.449 | 2.81905 | 1172.449 | 1.30885 |
| 1173.413 | 2.86663 | 1173.413 | 1.37672 |
| 1174.377 | 2.8917 | 1174.377 | 1.38896 |
| 1175.342 | 2.91119 | 1175.342 | 1.37249 |
| 1176.306 | 2.94393 | 1176.306 | 1.36835 |
| 1177.27 | 2.98037 | 1177.27 | 1.38644 |
| 1178.234 | 3.00121 | 1178.234 | 1.43877 |
| 1179.199 | 3.0105 | 1179.199 | 1.5178 |
| 1180.163 | 3.01977 | 1180.163 | 1.61254 |
| 1181.127 | 3.03482 | 1181.127 | 1.71429 |
| 1182.091 | 3.05153 | 1182.091 | 1.81023 |
| 1183.056 | 3.05426 | 1183.056 | 1.88114 |
| 1184.02 | 3.0368 | 1184.02 | 1.91963 |
| 1184.984 | 3.01455 | 1184.984 | 1.93525 |
| 1185.948 | 3.00217 | 1185.948 | 1.93853 |
| 1186.912 | 3.00935 | 1186.912 | 1.94918 |
| 1187.877 | 3.03129 | 1187.877 | 1.98451 |
| 1188.841 | 3.05629 | 1188.841 | 2.02462 |
| 1189.805 | 3.08327 | 1189.805 | 2.02541 |
| 1190.769 | 3.115 | 1190.769 | 1.99656 |
| 1191.734 | 3.1581 | 1191.734 | 1.98895 |
| 1192.698 | 3.23282 | 1192.698 | 2.01601 |
| 1193.662 | 3.33132 | 1193.662 | 2.05614 |
| 1194.626 | 3.43133 | 1194.626 | 2.08367 |
| 1195.591 | 3.51982 | 1195.591 | 2.10634 |
| 1196.555 | 3.60609 | 1196.555 | 2.14225 |
| 1197.519 | 3.70186 | 1197.519 | 2.2028 |
| 1198.483 | 3.79435 | 1198.483 | 2.28087 |
| 1199.448 | 3.86136 | 1199.448 | 2.33875 |
| 1200.412 | 3.90324 | 1200.412 | 2.35219 |
| 1201.376 | 3.93961 | 1201.376 | 2.3533 |
| 1202.34 | 3.98396 | 1202.34 | 2.37344 |
| 1203.304 | 4.04432 | 1203.304 | 2.4269 |
| 1204.269 | 4.13596 | 1204.269 | 2.51825 |
| 1205.233 | 4.25716 | 1205.233 | 2.62247 |
| 1206.197 | 4.3932 | 1206.197 | 2.71513 |
| 1207.161 | 4.54254 | 1207.161 | 2.77302 |
| 1208.126 | 4.70333 | 1208.126 | 2.79636 |
| 1209.09 | 4.86384 | 1209.09 | 2.82702 |
| 1210.054 | 5.02747 | 1210.054 | 2.88935 |
| 1211.018 | 5.20386 | 1211.018 | 3.0006 |
| 1211.983 | 5.38591 | 1211.983 | 3.12629 |
| 1212.947 | 5.55223 | 1212.947 | 3.18413 |
| 1213.911 | 5.68953 | 1213.911 | 3.15592 |
| 1214.875 | 5.79738 | 1214.875 | 3.09001 |
| 1215.839 | 5.88775 | 1215.839 | 3.0267 |
| 1216.804 | 5.97631 | 1216.804 | 2.96797 |
| 1217.768 | 6.07846 | 1217.768 | 2.91574 |
| 1218.732 | 6.20237 | 1218.732 | 2.88983 |
| 1219.696 | 6.33356 | 1219.696 | 2.88948 |
| 1220.661 | 6.45063 | 1220.661 | 2.89465 |
| 1221.625 | 6.54956 | 1221.625 | 2.89426 |
| 1222.589 | 6.64935 | 1222.589 | 2.91425 |
| 1223.553 | 6.76032 | 1223.553 | 2.96436 |
| 1224.518 | 6.86039 | 1224.518 | 3.01673 |
| 1225.482 | 6.92799 | 1225.482 | 3.0614 |
| 1226.446 | 6.98015 | 1226.446 | 3.10864 |
| 1227.41 | 7.04516 | 1227.41 | 3.1505 |
| 1228.375 | 7.11545 | 1228.375 | 3.17936 |
| 1229.339 | 7.16886 | 1229.339 | 3.19039 |
| 1230.303 | 7.19615 | 1230.303 | 3.17595 |
| 1231.267 | 7.193 | 1231.267 | 3.11327 |
| 1232.231 | 7.1705 | 1232.231 | 3.0078 |
| 1233.196 | 7.17091 | 1233.196 | 2.90659 |
| 1234.16 | 7.23341 | 1234.16 | 2.84856 |
| 1235.124 | 7.3493 | 1235.124 | 2.84371 |
| 1236.088 | 7.46655 | 1236.088 | 2.87555 |
| 1237.053 | 7.5553 | 1237.053 | 2.91088 |
| 1238.017 | 7.63874 | 1238.017 | 2.93382 |
| 1238.981 | 7.7446 | 1238.981 | 2.96857 |
| 1239.945 | 7.85693 | 1239.945 | 3.0524 |
| 1240.91 | 7.93673 | 1240.91 | 3.17307 |
| 1241.874 | 7.99923 | 1241.874 | 3.26277 |
| 1242.838 | 8.08543 | 1242.838 | 3.30882 |
| 1243.802 | 8.19183 | 1243.802 | 3.34899 |
| 1244.766 | 8.28595 | 1244.766 | 3.40165 |
| 1245.731 | 8.34509 | 1245.731 | 3.47279 |
| 1246.695 | 8.38306 | 1246.695 | 3.56534 |
| 1247.659 | 8.44191 | 1247.659 | 3.67504 |
| 1248.623 | 8.545 | 1248.623 | 3.78784 |
| 1249.588 | 8.67231 | 1249.588 | 3.88001 |
| 1250.552 | 8.79288 | 1250.552 | 3.94352 |
| 1251.516 | 8.91472 | 1251.516 | 3.99728 |
| 1252.48 | 9.06441 | 1252.48 | 4.03333 |
| 1253.445 | 9.23014 | 1253.445 | 4.03889 |
| 1254.409 | 9.39072 | 1254.409 | 4.03549 |
| 1255.373 | 9.54836 | 1255.373 | 4.03934 |
| 1256.337 | 9.71871 | 1256.337 | 4.059 |
| 1257.302 | 9.91298 | 1257.302 | 4.11581 |
| 1258.266 | 10.11667 | 1258.266 | 4.21297 |
| 1259.23 | 10.29429 | 1259.23 | 4.31608 |
| 1260.194 | 10.44957 | 1260.194 | 4.3788 |
| 1261.158 | 10.62436 | 1261.158 | 4.40486 |
| 1262.123 | 10.83513 | 1262.123 | 4.45262 |
| 1263.087 | 11.05896 | 1263.087 | 4.5404 |
| 1264.051 | 11.2745 | 1264.051 | 4.63553 |
| 1265.015 | 11.48731 | 1265.015 | 4.71328 |
| 1265.98 | 11.71827 | 1265.98 | 4.78315 |
| 1266.944 | 11.97528 | 1266.944 | 4.87382 |
| 1267.908 | 12.2437 | 1267.908 | 5.00385 |
| 1268.872 | 12.51209 | 1268.872 | 5.16488 |
| 1269.837 | 12.77395 | 1269.837 | 5.3241 |
| 1270.801 | 13.02841 | 1270.801 | 5.46583 |
| 1271.765 | 13.26816 | 1271.765 | 5.58772 |
| 1272.729 | 13.4849 | 1272.729 | 5.71493 |
| 1273.693 | 13.68764 | 1273.693 | 5.86623 |
| 1274.658 | 13.88925 | 1274.658 | 6.0095 |
| 1275.622 | 14.09109 | 1275.622 | 6.09678 |
| 1276.586 | 14.28824 | 1276.586 | 6.13637 |
| 1277.55 | 14.4837 | 1277.55 | 6.18573 |
| 1278.515 | 14.7 | 1278.515 | 6.26268 |
| 1279.479 | 14.97198 | 1279.479 | 6.33278 |
| 1280.443 | 15.32395 | 1280.443 | 6.41269 |
| 1281.407 | 15.74744 | 1281.407 | 6.54633 |
| 1282.372 | 16.19542 | 1282.372 | 6.73638 |
| 1283.336 | 16.61046 | 1283.336 | 6.94779 |
| 1284.3 | 16.96319 | 1284.3 | 7.14123 |
| 1285.264 | 17.27527 | 1285.264 | 7.28878 |
| 1286.229 | 17.6197 | 1286.229 | 7.38273 |
| 1287.193 | 18.0376 | 1287.193 | 7.43396 |
| 1288.157 | 18.4803 | 1288.157 | 7.50997 |
| 1289.121 | 18.91852 | 1289.121 | 7.6481 |
| 1290.085 | 19.39116 | 1290.085 | 7.80408 |
| 1291.05 | 19.90361 | 1291.05 | 7.96386 |
| 1292.014 | 20.41093 | 1292.014 | 8.13252 |
| 1292.978 | 20.87876 | 1292.978 | 8.29345 |
| 1293.942 | 21.31859 | 1293.942 | 8.42773 |
| 1294.907 | 21.77865 | 1294.907 | 8.55009 |
| 1295.871 | 22.30321 | 1295.871 | 8.70192 |
| 1296.835 | 22.88466 | 1296.835 | 8.89253 |
| 1297.799 | 23.45433 | 1297.799 | 9.07402 |
| 1298.764 | 23.96975 | 1298.764 | 9.22679 |
| 1299.728 | 24.49862 | 1299.728 | 9.38992 |
| 1300.692 | 25.09349 | 1300.692 | 9.58001 |
| 1301.656 | 25.72129 | 1301.656 | 9.8014 |
| 1302.62 | 26.34754 | 1302.62 | 10.04383 |
| 1303.585 | 26.97766 | 1303.585 | 10.29207 |
| 1304.549 | 27.63071 | 1304.549 | 10.53787 |
| 1305.513 | 28.30178 | 1305.513 | 10.77885 |
| 1306.477 | 28.96932 | 1306.477 | 11.01008 |
| 1307.442 | 29.63302 | 1307.442 | 11.23126 |
| 1308.406 | 30.32547 | 1308.406 | 11.44974 |
| 1309.37 | 31.04609 | 1309.37 | 11.67876 |
| 1310.334 | 31.75508 | 1310.334 | 11.92145 |
| 1311.299 | 32.43211 | 1311.299 | 12.16391 |
| 1312.263 | 33.09551 | 1312.263 | 12.39048 |
| 1313.227 | 33.77726 | 1313.227 | 12.6013 |
| 1314.191 | 34.48949 | 1314.191 | 12.81162 |
| 1315.156 | 35.214 | 1315.156 | 13.03376 |
| 1316.12 | 35.92087 | 1316.12 | 13.25959 |
| 1317.084 | 36.59481 | 1317.084 | 13.46219 |
| 1318.048 | 37.26226 | 1318.048 | 13.65684 |
| 1319.012 | 37.96207 | 1319.012 | 13.88837 |
| 1319.977 | 38.67817 | 1319.977 | 14.16704 |
| 1320.941 | 39.38552 | 1320.941 | 14.48288 |
| 1321.905 | 40.08888 | 1321.905 | 14.80207 |
| 1322.869 | 40.79942 | 1322.869 | 15.09836 |
| 1323.834 | 41.51968 | 1323.834 | 15.39174 |
| 1324.798 | 42.25165 | 1324.798 | 15.72983 |
| 1325.762 | 43.00571 | 1325.762 | 16.11839 |
| 1326.726 | 43.79551 | 1326.726 | 16.51584 |
| 1327.691 | 44.62384 | 1327.691 | 16.92298 |
| 1328.655 | 45.464 | 1328.655 | 17.39528 |
| 1329.619 | 46.25793 | 1329.619 | 17.91674 |
| 1330.583 | 46.96313 | 1330.583 | 18.42503 |
| 1331.547 | 47.59161 | 1331.547 | 18.88699 |
| 1332.512 | 48.19573 | 1332.512 | 19.31802 |
| 1333.476 | 48.81797 | 1333.476 | 19.7351 |
| 1334.44 | 49.45073 | 1334.44 | 20.12106 |
| 1335.404 | 50.02681 | 1335.404 | 20.46252 |
| 1336.369 | 50.49348 | 1336.369 | 20.81594 |
| 1337.333 | 50.87132 | 1337.333 | 21.2086 |
| 1338.297 | 51.21566 | 1338.297 | 21.59588 |
| 1339.261 | 51.57179 | 1339.261 | 21.9627 |
| 1340.226 | 51.91859 | 1340.226 | 22.30519 |
| 1341.19 | 52.20891 | 1341.19 | 22.61815 |
| 1342.154 | 52.43319 | 1342.154 | 22.92127 |
| 1343.118 | 52.61351 | 1343.118 | 23.25285 |
| 1344.083 | 52.75386 | 1344.083 | 23.62031 |
| 1345.047 | 52.82883 | 1345.047 | 23.96744 |
| 1346.011 | 52.84209 | 1346.011 | 24.22235 |
| 1346.975 | 52.86997 | 1346.975 | 24.3859 |
| 1347.939 | 52.93928 | 1347.939 | 24.50785 |
| 1348.904 | 52.94922 | 1348.904 | 24.59161 |
| 1349.868 | 52.85649 | 1349.868 | 24.63089 |
| 1350.832 | 52.68489 | 1350.832 | 24.63661 |
| 1351.796 | 52.46181 | 1351.796 | 24.60961 |
| 1352.761 | 52.19451 | 1352.761 | 24.53601 |
| 1353.725 | 51.87207 | 1353.725 | 24.40994 |
| 1354.689 | 51.48405 | 1354.689 | 24.24334 |
| 1355.653 | 51.03201 | 1355.653 | 24.05258 |
| 1356.618 | 50.52313 | 1356.618 | 23.8448 |
| 1357.582 | 49.95918 | 1357.582 | 23.61463 |
| 1358.546 | 49.34799 | 1358.546 | 23.34955 |
| 1359.51 | 48.72096 | 1359.51 | 23.0555 |
| 1360.474 | 48.10376 | 1360.474 | 22.75301 |
| 1361.439 | 47.49476 | 1361.439 | 22.44232 |
| 1362.403 | 46.88974 | 1362.403 | 22.10663 |
| 1363.367 | 46.2727 | 1363.367 | 21.73592 |
| 1364.331 | 45.62804 | 1364.331 | 21.33247 |
| 1365.296 | 44.95693 | 1365.296 | 20.91814 |
| 1366.26 | 44.27136 | 1366.26 | 20.53204 |
| 1367.224 | 43.54579 | 1367.224 | 20.16302 |
| 1368.188 | 42.75557 | 1368.188 | 19.76955 |
| 1369.153 | 41.93653 | 1369.153 | 19.34516 |
| 1370.117 | 41.11956 | 1370.117 | 18.8887 |
| 1371.081 | 40.30413 | 1371.081 | 18.39987 |
| 1372.045 | 39.49295 | 1372.045 | 17.9038 |
| 1373.01 | 38.69537 | 1373.01 | 17.43853 |
| 1373.974 | 37.91388 | 1373.974 | 17.02337 |
| 1374.938 | 37.1388 | 1374.938 | 16.65223 |
| 1375.902 | 36.35702 | 1375.902 | 16.30378 |
| 1376.866 | 35.55954 | 1376.866 | 15.94116 |
| 1377.831 | 34.75834 | 1377.831 | 15.53683 |
| 1378.795 | 34.00852 | 1378.795 | 15.12506 |
| 1379.759 | 33.30029 | 1379.759 | 14.73465 |
| 1380.723 | 32.55464 | 1380.723 | 14.35617 |
| 1381.688 | 31.75686 | 1381.688 | 13.96118 |
| 1382.652 | 30.93436 | 1382.652 | 13.54048 |
| 1383.616 | 30.13189 | 1383.616 | 13.1299 |
| 1384.58 | 29.38284 | 1384.58 | 12.76947 |
| 1385.545 | 28.68487 | 1385.545 | 12.44785 |
| 1386.509 | 28.0352 | 1386.509 | 12.12527 |
| 1387.473 | 27.42204 | 1387.473 | 11.79189 |
| 1388.437 | 26.80169 | 1388.437 | 11.46436 |
| 1389.401 | 26.16833 | 1389.401 | 11.17683 |
| 1390.366 | 25.56052 | 1390.366 | 10.95609 |
| 1391.33 | 25.02662 | 1391.33 | 10.7783 |
| 1392.294 | 24.59523 | 1392.294 | 10.60009 |
| 1393.258 | 24.23506 | 1393.258 | 10.3927 |
| 1394.223 | 23.89421 | 1394.223 | 10.13789 |
| 1395.187 | 23.5318 | 1395.187 | 9.8674 |
| 1396.151 | 23.11269 | 1396.151 | 9.63009 |
| 1397.115 | 22.66915 | 1397.115 | 9.4307 |
| 1398.08 | 22.2673 | 1398.08 | 9.25779 |
| 1399.044 | 21.8877 | 1399.044 | 9.09742 |
| 1400.008 | 21.51729 | 1400.008 | 8.92406 |
| 1400.972 | 21.18305 | 1400.972 | 8.72392 |
| 1401.937 | 20.88329 | 1401.937 | 8.52272 |
| 1402.901 | 20.57401 | 1402.901 | 8.35614 |
| 1403.865 | 20.24596 | 1403.865 | 8.23245 |
| 1404.829 | 19.927 | 1404.829 | 8.14811 |
| 1405.793 | 19.64326 | 1405.793 | 8.10149 |
| 1406.758 | 19.40747 | 1406.758 | 8.08639 |
| 1407.722 | 19.21936 | 1407.722 | 8.0927 |
| 1408.686 | 19.04705 | 1408.686 | 8.08771 |
| 1409.65 | 18.85184 | 1409.65 | 8.01526 |
| 1410.615 | 18.62083 | 1410.615 | 7.85703 |
| 1411.579 | 18.34789 | 1411.579 | 7.68318 |
| 1412.543 | 18.03216 | 1412.543 | 7.56225 |
| 1413.507 | 17.68783 | 1413.507 | 7.47305 |
| 1414.472 | 17.33273 | 1414.472 | 7.36717 |
| 1415.436 | 16.98994 | 1415.436 | 7.23965 |
| 1416.4 | 16.69297 | 1416.4 | 7.10272 |
| 1417.364 | 16.43632 | 1417.364 | 6.96748 |
| 1418.328 | 16.17983 | 1418.328 | 6.83707 |
| 1419.293 | 15.90539 | 1419.293 | 6.71387 |
| 1420.257 | 15.6263 | 1420.257 | 6.61296 |
| 1421.221 | 15.35293 | 1421.221 | 6.54188 |
| 1422.185 | 15.08588 | 1422.185 | 6.48845 |
| 1423.15 | 14.82796 | 1423.15 | 6.43206 |
| 1424.114 | 14.58442 | 1424.114 | 6.36327 |
| 1425.078 | 14.3648 | 1425.078 | 6.28849 |
| 1426.042 | 14.1724 | 1426.042 | 6.20766 |
| 1427.007 | 13.97224 | 1427.007 | 6.11454 |
| 1427.971 | 13.71659 | 1427.971 | 6.00418 |
| 1428.935 | 13.43033 | 1428.935 | 5.8793 |
| 1429.899 | 13.17479 | 1429.899 | 5.73181 |
| 1430.864 | 12.93914 | 1430.864 | 5.56219 |
| 1431.828 | 12.72269 | 1431.828 | 5.40532 |
| 1432.792 | 12.55172 | 1432.792 | 5.29747 |
| 1433.756 | 12.41983 | 1433.756 | 5.23415 |
| 1434.72 | 12.28111 | 1434.72 | 5.17722 |
| 1435.685 | 12.11667 | 1435.685 | 5.11287 |
| 1436.649 | 11.96605 | 1436.649 | 5.06673 |
| 1437.613 | 11.85835 | 1437.613 | 5.04309 |
| 1438.577 | 11.79411 | 1438.577 | 5.01509 |
| 1439.542 | 11.77028 | 1439.542 | 4.96718 |
| 1440.506 | 11.75954 | 1440.506 | 4.90919 |
| 1441.47 | 11.7211 | 1441.47 | 4.84583 |
| 1442.434 | 11.66117 | 1442.434 | 4.7758 |
| 1443.399 | 11.60626 | 1443.399 | 4.72673 |
| 1444.363 | 11.55863 | 1444.363 | 4.7064 |
| 1445.327 | 11.51503 | 1445.327 | 4.68579 |
| 1446.291 | 11.48377 | 1446.291 | 4.63959 |
| 1447.255 | 11.46748 | 1447.255 | 4.56852 |
| 1448.22 | 11.44828 | 1448.22 | 4.47905 |
| 1449.184 | 11.40585 | 1449.184 | 4.39031 |
| 1450.148 | 11.31809 | 1450.148 | 4.33913 |
| 1451.112 | 11.19354 | 1451.112 | 4.33983 |
| 1452.077 | 11.08509 | 1452.077 | 4.37762 |
| 1453.041 | 11.01978 | 1453.041 | 4.4372 |
| 1454.005 | 10.98421 | 1454.005 | 4.50006 |
| 1454.969 | 10.95931 | 1454.969 | 4.54997 |
| 1455.934 | 10.93593 | 1455.934 | 4.57623 |
| 1456.898 | 10.90327 | 1456.898 | 4.55401 |
| 1457.862 | 10.85591 | 1457.862 | 4.47177 |
| 1458.826 | 10.79184 | 1458.826 | 4.37437 |
| 1459.791 | 10.70055 | 1459.791 | 4.30648 |
| 1460.755 | 10.59805 | 1460.755 | 4.25321 |
| 1461.719 | 10.51833 | 1461.719 | 4.17093 |
| 1462.683 | 10.46437 | 1462.683 | 4.07588 |
| 1463.647 | 10.41829 | 1463.647 | 4.01774 |
| 1464.612 | 10.36663 | 1464.612 | 4.00539 |
| 1465.576 | 10.30076 | 1465.576 | 4.02235 |
| 1466.54 | 10.22465 | 1466.54 | 4.05266 |
| 1467.504 | 10.14496 | 1467.504 | 4.08518 |
| 1468.469 | 10.05959 | 1468.469 | 4.10607 |
| 1469.433 | 9.96772 | 1469.433 | 4.10424 |
| 1470.397 | 9.88414 | 1470.397 | 4.08904 |
| 1471.361 | 9.8145 | 1471.361 | 4.07355 |
| 1472.326 | 9.77918 | 1472.326 | 4.06225 |
| 1473.29 | 9.82563 | 1473.29 | 4.06217 |
| 1474.254 | 9.91343 | 1474.254 | 4.07294 |
| 1475.218 | 9.95383 | 1475.218 | 4.09288 |
| 1476.182 | 9.93705 | 1476.182 | 4.12188 |
| 1477.147 | 9.91207 | 1477.147 | 4.15967 |
| 1478.111 | 9.9184 | 1478.111 | 4.21234 |
| 1479.075 | 9.95647 | 1479.075 | 4.2952 |
| 1480.039 | 9.99724 | 1480.039 | 4.40975 |
| 1481.004 | 10.00706 | 1481.004 | 4.52167 |
| 1481.968 | 9.97046 | 1481.968 | 4.58162 |
| 1482.932 | 9.90774 | 1482.932 | 4.58025 |
| 1483.896 | 9.86845 | 1483.896 | 4.56102 |
| 1484.861 | 9.88684 | 1484.861 | 4.55703 |
| 1485.825 | 9.94058 | 1485.825 | 4.56355 |
| 1486.789 | 10.00462 | 1486.789 | 4.56932 |
| 1487.753 | 10.06971 | 1487.753 | 4.57475 |
| 1488.718 | 10.12081 | 1488.718 | 4.58148 |
| 1489.682 | 10.1559 | 1489.682 | 4.59035 |
| 1490.646 | 10.1808 | 1490.646 | 4.59953 |
| 1491.61 | 10.20188 | 1491.61 | 4.60243 |
| 1492.574 | 10.23005 | 1492.574 | 4.58901 |
| 1493.539 | 10.27615 | 1493.539 | 4.55346 |
| 1494.503 | 10.3293 | 1494.503 | 4.50627 |
| 1495.467 | 10.36318 | 1495.467 | 4.46411 |
| 1496.431 | 10.37274 | 1496.431 | 4.43764 |
| 1497.396 | 10.37451 | 1497.396 | 4.44335 |
| 1498.36 | 10.37219 | 1498.36 | 4.48774 |
| 1499.324 | 10.37771 | 1499.324 | 4.55133 |
| 1500.288 | 10.41175 | 1500.288 | 4.6122 |
| 1501.253 | 10.46314 | 1501.253 | 4.66025 |
| 1502.217 | 10.49228 | 1502.217 | 4.68747 |
| 1503.181 | 10.493 | 1503.181 | 4.69179 |
| 1504.145 | 10.50877 | 1504.145 | 4.67523 |
| 1505.109 | 10.56963 | 1505.109 | 4.64184 |
| 1506.074 | 10.64123 | 1506.074 | 4.60816 |
| 1507.038 | 10.6714 | 1507.038 | 4.5972 |
| 1508.002 | 10.67779 | 1508.002 | 4.61324 |
| 1508.966 | 10.73402 | 1508.966 | 4.66633 |
| 1509.931 | 10.85212 | 1509.931 | 4.74854 |
| 1510.895 | 10.99152 | 1510.895 | 4.83341 |
| 1511.859 | 11.13134 | 1511.859 | 4.91023 |
| 1512.823 | 11.27726 | 1512.823 | 5.00307 |
| 1513.788 | 11.43629 | 1513.788 | 5.14334 |
| 1514.752 | 11.60106 | 1514.752 | 5.30177 |
| 1515.716 | 11.75822 | 1515.716 | 5.40164 |
| 1516.68 | 11.90356 | 1516.68 | 5.42363 |
| 1517.645 | 12.04563 | 1517.645 | 5.43621 |
| 1518.609 | 12.19473 | 1518.609 | 5.49756 |
| 1519.573 | 12.35122 | 1519.573 | 5.59973 |
| 1520.537 | 12.51725 | 1520.537 | 5.72339 |
| 1521.501 | 12.69135 | 1521.501 | 5.86524 |
| 1522.466 | 12.8488 | 1522.466 | 6.01775 |
| 1523.43 | 13.00265 | 1523.43 | 6.18509 |
| 1524.394 | 13.2128 | 1524.394 | 6.39223 |
| 1525.358 | 13.48509 | 1525.358 | 6.63614 |
| 1526.323 | 13.74864 | 1526.323 | 6.87072 |
| 1527.287 | 13.94034 | 1527.287 | 7.05344 |
| 1528.251 | 14.07367 | 1528.251 | 7.18019 |
| 1529.215 | 14.204 | 1529.215 | 7.26852 |
| 1530.18 | 14.36332 | 1530.18 | 7.33669 |
| 1531.144 | 14.55469 | 1531.144 | 7.4085 |
| 1532.108 | 14.77802 | 1532.108 | 7.50571 |
| 1533.072 | 15.02146 | 1533.072 | 7.61494 |
| 1534.036 | 15.27515 | 1534.036 | 7.72062 |
| 1535.001 | 15.5317 | 1535.001 | 7.82454 |
| 1535.965 | 15.78166 | 1535.965 | 7.93713 |
| 1536.929 | 16.02488 | 1536.929 | 8.05317 |
| 1537.893 | 16.26027 | 1537.893 | 8.1425 |
| 1538.858 | 16.49786 | 1538.858 | 8.20428 |
| 1539.822 | 16.76519 | 1539.822 | 8.28906 |
| 1540.786 | 17.07524 | 1540.786 | 8.42988 |
| 1541.75 | 17.39828 | 1541.75 | 8.58539 |
| 1542.715 | 17.68731 | 1542.715 | 8.6876 |
| 1543.679 | 17.93556 | 1543.679 | 8.73464 |
| 1544.643 | 18.17912 | 1544.643 | 8.80362 |
| 1545.607 | 18.45007 | 1545.607 | 8.9472 |
| 1546.572 | 18.77119 | 1546.572 | 9.1118 |
| 1547.536 | 19.17263 | 1547.536 | 9.23465 |
| 1548.5 | 19.62559 | 1548.5 | 9.33659 |
| 1549.464 | 20.08218 | 1549.464 | 9.45511 |
| 1550.428 | 20.53102 | 1550.428 | 9.59316 |
| 1551.393 | 20.98849 | 1551.393 | 9.74949 |
| 1552.357 | 21.47382 | 1552.357 | 9.92325 |
| 1553.321 | 21.96548 | 1553.321 | 10.10078 |
| 1554.285 | 22.43831 | 1554.285 | 10.28197 |
| 1555.25 | 22.9329 | 1555.25 | 10.49826 |
| 1556.214 | 23.51974 | 1556.214 | 10.78439 |
| 1557.178 | 24.2049 | 1557.178 | 11.14987 |
| 1558.142 | 24.93621 | 1558.142 | 11.58398 |
| 1559.107 | 25.69227 | 1559.107 | 12.08926 |
| 1560.071 | 26.50987 | 1560.071 | 12.7074 |
| 1561.035 | 27.42597 | 1561.035 | 13.46663 |
| 1561.999 | 28.42302 | 1561.999 | 14.34843 |
| 1562.964 | 29.4652 | 1562.964 | 15.31734 |
| 1563.928 | 30.57135 | 1563.928 | 16.37554 |
| 1564.892 | 31.76905 | 1564.892 | 17.60203 |
| 1565.856 | 33.05921 | 1565.856 | 19.01938 |
| 1566.82 | 34.43766 | 1566.82 | 20.55305 |
| 1567.785 | 35.90049 | 1567.785 | 22.14077 |
| 1568.749 | 37.43902 | 1568.749 | 23.78964 |
| 1569.713 | 39.05094 | 1569.713 | 25.50206 |
| 1570.677 | 40.70832 | 1570.677 | 27.22172 |
| 1571.642 | 42.34968 | 1571.642 | 28.87169 |
| 1572.606 | 43.94872 | 1572.606 | 30.42457 |
| 1573.57 | 45.50314 | 1573.57 | 31.91084 |
| 1574.534 | 47.01503 | 1574.534 | 33.35926 |
| 1575.499 | 48.47583 | 1575.499 | 34.75158 |
| 1576.463 | 49.8602 | 1576.463 | 36.0774 |
| 1577.427 | 51.20724 | 1577.427 | 37.32772 |
| 1578.391 | 52.56212 | 1578.391 | 38.47602 |
| 1579.355 | 53.89816 | 1579.355 | 39.5067 |
| 1580.32 | 55.16853 | 1580.32 | 40.40678 |
| 1581.284 | 56.31182 | 1581.284 | 41.15152 |
| 1582.248 | 57.27525 | 1582.248 | 41.7255 |
| 1583.212 | 58.03985 | 1583.212 | 42.13743 |
| 1584.177 | 58.63847 | 1584.177 | 42.4161 |
| 1585.141 | 59.12041 | 1585.141 | 42.58864 |
| 1586.105 | 59.50046 | 1586.105 | 42.64527 |
| 1587.069 | 59.77679 | 1587.069 | 42.55348 |
| 1588.034 | 59.94448 | 1588.034 | 42.29204 |
| 1588.998 | 59.99238 | 1588.998 | 41.85247 |
| 1589.962 | 59.91196 | 1589.962 | 41.23373 |
| 1590.926 | 59.71648 | 1590.926 | 40.47021 |
| 1591.891 | 59.45027 | 1591.891 | 39.63724 |
| 1592.855 | 59.11969 | 1592.855 | 38.76757 |
| 1593.819 | 58.70226 | 1593.819 | 37.81766 |
| 1594.783 | 58.15402 | 1594.783 | 36.71789 |
| 1595.747 | 57.41827 | 1595.747 | 35.46966 |
| 1596.712 | 56.48154 | 1596.712 | 34.13112 |
| 1597.676 | 55.4256 | 1597.676 | 32.73871 |
| 1598.64 | 54.3261 | 1598.64 | 31.30815 |
| 1599.604 | 53.19345 | 1599.604 | 29.85314 |
| 1600.569 | 52.02728 | 1600.569 | 28.38376 |
| 1601.533 | 50.83604 | 1601.533 | 26.92564 |
| 1602.497 | 49.63971 | 1602.497 | 25.53223 |
| 1603.461 | 48.4482 | 1603.461 | 24.23794 |
| 1604.426 | 47.24677 | 1604.426 | 23.01122 |
| 1605.39 | 46.01753 | 1605.39 | 21.7955 |
| 1606.354 | 44.78562 | 1606.354 | 20.64052 |
| 1607.318 | 43.59213 | 1607.318 | 19.63122 |
| 1608.282 | 42.45437 | 1608.282 | 18.78955 |
| 1609.247 | 41.35877 | 1609.247 | 18.09813 |
| 1610.211 | 40.27111 | 1610.211 | 17.53176 |
| 1611.175 | 39.18222 | 1611.175 | 17.05431 |
| 1612.139 | 38.13161 | 1612.139 | 16.62844 |
| 1613.104 | 37.15568 | 1613.104 | 16.2533 |
| 1614.068 | 36.26411 | 1614.068 | 15.91206 |
| 1615.032 | 35.41811 | 1615.032 | 15.58318 |
| 1615.996 | 34.55085 | 1615.996 | 15.25357 |
| 1616.961 | 33.63515 | 1616.961 | 14.91605 |
| 1617.925 | 32.69128 | 1617.925 | 14.54732 |
| 1618.889 | 31.73589 | 1618.889 | 14.12353 |
| 1619.853 | 30.78881 | 1619.853 | 13.69232 |
| 1620.818 | 29.88 | 1620.818 | 13.35748 |
| 1621.782 | 28.99964 | 1621.782 | 13.13334 |
| 1622.746 | 28.13245 | 1622.746 | 12.95754 |
| 1623.71 | 27.25382 | 1623.71 | 12.73695 |
| 1624.674 | 26.35749 | 1624.674 | 12.43248 |
| 1625.639 | 25.48884 | 1625.639 | 12.05799 |
| 1626.603 | 24.63227 | 1626.603 | 11.64772 |
| 1627.567 | 23.73228 | 1627.567 | 11.23455 |
| 1628.531 | 22.77977 | 1628.531 | 10.82212 |
| 1629.496 | 21.77116 | 1629.496 | 10.39871 |
| 1630.46 | 20.71426 | 1630.46 | 9.96108 |
| 1631.424 | 19.64948 | 1631.424 | 9.52682 |
| 1632.388 | 18.61874 | 1632.388 | 9.11265 |
| 1633.353 | 17.63683 | 1633.353 | 8.7102 |
| 1634.317 | 16.69199 | 1634.317 | 8.29357 |
| 1635.281 | 15.77595 | 1635.281 | 7.86884 |
| 1636.245 | 14.90882 | 1636.245 | 7.49742 |
| 1637.209 | 14.09622 | 1637.209 | 7.18839 |
| 1638.174 | 13.31807 | 1638.174 | 6.90081 |
| 1639.138 | 12.55718 | 1639.138 | 6.60337 |
| 1640.102 | 11.81506 | 1640.102 | 6.28938 |
| 1641.066 | 11.07848 | 1641.066 | 5.96481 |
| 1642.031 | 10.33635 | 1642.031 | 5.64022 |
| 1642.995 | 9.57977 | 1642.995 | 5.30891 |
| 1643.959 | 8.84882 | 1643.959 | 4.97937 |
| 1644.923 | 8.23428 | 1644.923 | 4.68913 |
| 1645.888 | 7.76821 | 1645.888 | 4.4593 |
| 1646.852 | 7.38981 | 1646.852 | 4.27436 |
| 1647.816 | 7.01914 | 1647.816 | 4.10188 |
| 1648.78 | 6.60531 | 1648.78 | 3.9077 |
| 1649.745 | 6.13483 | 1649.745 | 3.67349 |
| 1650.709 | 5.66652 | 1650.709 | 3.43354 |
| 1651.673 | 5.24262 | 1651.673 | 3.2107 |
| 1652.637 | 4.8485 | 1652.637 | 2.99086 |
| 1653.601 | 4.49355 | 1653.601 | 2.80646 |
| 1654.566 | 4.19427 | 1654.566 | 2.70458 |
| 1655.53 | 3.93032 | 1655.53 | 2.64168 |
| 1656.494 | 3.68577 | 1656.494 | 2.55697 |
| 1657.458 | 3.47553 | 1657.458 | 2.4398 |
| 1658.423 | 3.30058 | 1658.423 | 2.29221 |
| 1659.387 | 3.13894 | 1659.387 | 2.13052 |
| 1660.351 | 2.98926 | 1660.351 | 1.98472 |
| 1661.315 | 2.86804 | 1661.315 | 1.86536 |
| 1662.28 | 2.76386 | 1662.28 | 1.74826 |
| 1663.244 | 2.64009 | 1663.244 | 1.61038 |
| 1664.208 | 2.49908 | 1664.208 | 1.46933 |
| 1665.172 | 2.39717 | 1665.172 | 1.36467 |
| 1666.136 | 2.36352 | 1666.136 | 1.31446 |
| 1667.101 | 2.38768 | 1667.101 | 1.3289 |
| 1668.065 | 2.44441 | 1668.065 | 1.37684 |
| 1669.029 | 2.48208 | 1669.029 | 1.38354 |
| 1669.993 | 2.48596 | 1669.993 | 1.32439 |
| 1670.958 | 2.50625 | 1670.958 | 1.25046 |
| 1671.922 | 2.54151 | 1671.922 | 1.16265 |
| 1672.886 | 2.5572 | 1672.886 | 1.05584 |
| 1673.85 | 2.54485 | 1673.85 | 0.96139 |
| 1674.815 | 2.51297 | 1674.815 | 0.89419 |
| 1675.779 | 2.46274 | 1675.779 | 0.83714 |
| 1676.743 | 2.39096 | 1676.743 | 0.77144 |
| 1677.707 | 2.29808 | 1677.707 | 0.69347 |
| 1678.672 | 2.19086 | 1678.672 | 0.60983 |
| 1679.636 | 2.09057 | 1679.636 | 0.53377 |
| 1680.6 | 2.02772 | 1680.6 | 0.48138 |
| 1681.564 | 2.00671 | 1681.564 | 0.47359 |
| 1682.528 | 2.00787 | 1682.528 | 0.52393 |
| 1683.493 | 2.00599 | 1683.493 | 0.59836 |
| 1684.457 | 1.97647 | 1684.457 | 0.63656 |
| 1685.421 | 1.93105 | 1685.421 | 0.62201 |
| 1686.385 | 1.91495 | 1686.385 | 0.61046 |
| 1687.35 | 1.94054 | 1687.35 | 0.62911 |
| 1688.314 | 1.98147 | 1688.314 | 0.66108 |
| 1689.278 | 2.01332 | 1689.278 | 0.69943 |
| 1690.242 | 2.03959 | 1690.242 | 0.74791 |
| 1691.207 | 2.085 | 1691.207 | 0.80296 |
| 1692.171 | 2.15779 | 1692.171 | 0.85343 |
| 1693.135 | 2.23041 | 1693.135 | 0.88881 |
| 1694.099 | 2.26822 | 1694.099 | 0.90669 |
| 1695.063 | 2.26158 | 1695.063 | 0.90983 |
| 1696.028 | 2.20972 | 1696.028 | 0.89249 |
| 1696.992 | 2.10164 | 1696.992 | 0.84457 |
| 1697.956 | 1.96526 | 1697.956 | 0.78825 |
| 1698.92 | 1.87909 | 1698.92 | 0.77878 |
| 1699.885 | 1.84441 | 1699.885 | 0.81826 |
| 1700.849 | 1.81317 | 1700.849 | 0.8627 |
| 1701.813 | 1.77834 | 1701.813 | 0.88582 |
| 1702.777 | 1.73254 | 1702.777 | 0.87105 |
| 1703.741 | 1.66836 | 1703.741 | 0.83414 |
| 1704.706 | 1.59675 | 1704.706 | 0.79638 |
| 1705.67 | 1.53755 | 1705.67 | 0.75314 |
| 1706.634 | 1.48913 | 1706.634 | 0.69593 |
| 1707.598 | 1.43245 | 1707.598 | 0.64523 |
| 1708.563 | 1.3709 | 1708.563 | 0.63136 |
| 1709.527 | 1.33818 | 1709.527 | 0.64211 |
| 1710.491 | 1.35184 | 1710.491 | 0.64171 |
| 1711.455 | 1.39027 | 1711.455 | 0.62469 |
| 1712.42 | 1.4261 | 1712.42 | 0.60621 |
| 1713.384 | 1.45164 | 1713.384 | 0.58716 |
| 1714.348 | 1.46689 | 1714.348 | 0.57386 |
| 1715.312 | 1.48189 | 1715.312 | 0.58277 |
| 1716.276 | 1.48207 | 1716.276 | 0.56774 |
| 1717.241 | 1.45531 | 1717.241 | 0.49482 |
| 1718.205 | 1.43902 | 1718.205 | 0.41807 |
| 1719.169 | 1.44278 | 1719.169 | 0.38465 |
| 1720.133 | 1.42638 | 1720.133 | 0.37793 |
| 1721.098 | 1.37971 | 1721.098 | 0.3522 |
| 1722.062 | 1.34094 | 1722.062 | 0.29474 |
| 1723.026 | 1.33074 | 1723.026 | 0.23277 |
| 1723.99 | 1.34502 | 1723.99 | 0.19881 |
| 1724.955 | 1.36962 | 1724.955 | 0.18605 |
| 1725.919 | 1.38038 | 1725.919 | 0.16732 |
| 1726.883 | 1.38694 | 1726.883 | 0.14819 |
| 1727.847 | 1.4328 | 1727.847 | 0.16156 |
| 1728.812 | 1.52436 | 1728.812 | 0.22051 |
| 1729.776 | 1.61387 | 1729.776 | 0.31119 |
| 1730.74 | 1.67096 | 1730.74 | 0.4035 |
| 1731.704 | 1.70081 | 1731.704 | 0.45982 |
| 1732.668 | 1.72842 | 1732.668 | 0.49076 |
| 1733.633 | 1.79409 | 1733.633 | 0.55732 |
| 1734.597 | 1.897 | 1734.597 | 0.67852 |
| 1735.561 | 1.99239 | 1735.561 | 0.814 |
| 1736.525 | 2.03797 | 1736.525 | 0.90464 |
| 1737.49 | 2.02287 | 1737.49 | 0.91966 |
| 1738.454 | 1.98568 | 1738.454 | 0.91632 |
| 1739.418 | 1.94613 | 1739.418 | 0.94884 |
| 1740.382 | 1.90521 | 1740.382 | 1.00497 |
| 1741.347 | 1.8747 | 1741.347 | 1.08117 |
| 1742.311 | 1.86123 | 1742.311 | 1.16722 |
| 1743.275 | 1.86914 | 1743.275 | 1.23941 |
| 1744.239 | 1.8767 | 1744.239 | 1.28121 |
| 1745.203 | 1.84765 | 1745.203 | 1.28801 |
| 1746.168 | 1.78339 | 1746.168 | 1.27406 |
| 1747.132 | 1.7156 | 1747.132 | 1.26145 |
| 1748.096 | 1.65472 | 1748.096 | 1.25251 |
| 1749.06 | 1.59745 | 1749.06 | 1.23154 |
| 1750.025 | 1.55578 | 1750.025 | 1.20157 |
| 1750.989 | 1.53053 | 1750.989 | 1.17899 |
| 1751.953 | 1.492 | 1751.953 | 1.1579 |
| 1752.917 | 1.42821 | 1752.917 | 1.11785 |
| 1753.882 | 1.37619 | 1753.882 | 1.06205 |
| 1754.846 | 1.35411 | 1754.846 | 1.02073 |
| 1755.81 | 1.32848 | 1755.81 | 0.99122 |
| 1756.774 | 1.27525 | 1756.774 | 0.91574 |
| 1757.739 | 1.21841 | 1757.739 | 0.78482 |
| 1758.703 | 1.18621 | 1758.703 | 0.65893 |
| 1759.667 | 1.17192 | 1759.667 | 0.57508 |
| 1760.631 | 1.16425 | 1760.631 | 0.52857 |
| 1761.595 | 1.15622 | 1761.595 | 0.50127 |
| 1762.56 | 1.12853 | 1762.56 | 0.48074 |
| 1763.524 | 1.06013 | 1763.524 | 0.44297 |
| 1764.488 | 0.96206 | 1764.488 | 0.36051 |
| 1765.452 | 0.87807 | 1765.452 | 0.25343 |
| 1766.417 | 0.83662 | 1766.417 | 0.17683 |
| 1767.381 | 0.83071 | 1767.381 | 0.14463 |
| 1768.345 | 0.83973 | 1768.345 | 0.11741 |
| 1769.309 | 0.84607 | 1769.309 | 0.07013 |
| 1770.274 | 0.83816 | 1770.274 | 0.0256 |
| 1771.238 | 0.81711 | 1771.238 | 0.00204 |
| 1772.202 | 0.80041 | 1772.202 | -0.01499 |
| 1773.166 | 0.78155 | 1773.166 | -0.0541 |
| 1774.13 | 0.7467 | 1774.13 | -0.11071 |
| 1775.095 | 0.7257 | 1775.095 | -0.15235 |
| 1776.059 | 0.73687 | 1776.059 | -0.17537 |
| 1777.023 | 0.74735 | 1777.023 | -0.19934 |
| 1777.987 | 0.73737 | 1777.987 | -0.21222 |
| 1778.952 | 0.73548 | 1778.952 | -0.1859 |
| 1779.916 | 0.75771 | 1779.916 | -0.14496 |
| 1780.88 | 0.79345 | 1780.88 | -0.12363 |
| 1781.844 | 0.82422 | 1781.844 | -0.10603 |
| 1782.809 | 0.82943 | 1782.809 | -0.07508 |
| 1783.773 | 0.80175 | 1783.773 | -0.0586 |
| 1784.737 | 0.74493 | 1784.737 | -0.08839 |
| 1785.701 | 0.68743 | 1785.701 | -0.12901 |
| 1786.666 | 0.66671 | 1786.666 | -0.10698 |
| 1787.63 | 0.67663 | 1787.63 | -0.0396 |
| 1788.594 | 0.71283 | 1788.594 | 0.01241 |
| 1789.558 | 0.77334 | 1789.558 | 0.03805 |
| 1790.522 | 0.8328 | 1790.522 | 0.0328 |
| 1791.487 | 0.86964 | 1791.487 | 0.01083 |
| 1792.451 | 0.86891 | 1792.451 | -0.00477 |
| 1793.415 | 0.83948 | 1793.415 | -0.00881 |
| 1794.379 | 0.80865 | 1794.379 | -0.00305 |
| 1795.344 | 0.78603 | 1795.344 | 0.00551 |
| 1796.308 | 0.76135 | 1796.308 | 0.00173 |
| 1797.272 | 0.73551 | 1797.272 | -0.00273 |
| 1798.236 | 0.70104 | 1798.236 | 0.00638 |
| 1799.201 | 0.6468 | 1799.201 | 0.01979 |
| 1800.165 | 0.58277 | 1800.165 | 0.03874 |
| 1801.129 | 0.53449 | 1801.129 | 0.05728 |
| 1802.093 | 0.52473 | 1802.093 | 0.05346 |
| 1803.057 | 0.52636 | 1803.057 | 0.01876 |
| 1804.022 | 0.49676 | 1804.022 | -0.03828 |
| 1804.986 | 0.44246 | 1804.986 | -0.07902 |
| 1805.95 | 0.39927 | 1805.95 | -0.04623 |
| 1806.914 | 0.37647 | 1806.914 | 0.0428 |
| 1807.879 | 0.36785 | 1807.879 | 0.11365 |
| 1808.843 | 0.38996 | 1808.843 | 0.13604 |
| 1809.807 | 0.42978 | 1809.807 | 0.12794 |
| 1810.771 | 0.44569 | 1810.771 | 0.12089 |
| 1811.736 | 0.44255 | 1811.736 | 0.12584 |
| 1812.7 | 0.46361 | 1812.7 | 0.13242 |
| 1813.664 | 0.50856 | 1813.664 | 0.12676 |
| 1814.628 | 0.5358 | 1814.628 | 0.09535 |
| 1815.593 | 0.5283 | 1815.593 | 0.03476 |
| 1816.557 | 0.49605 | 1816.557 | -0.0338 |
| 1817.521 | 0.44736 | 1817.521 | -0.09029 |
| 1818.485 | 0.39644 | 1818.485 | -0.13473 |
| 1819.449 | 0.35942 | 1819.449 | -0.16625 |
| 1820.414 | 0.33001 | 1820.414 | -0.1839 |
| 1821.378 | 0.29462 | 1821.378 | -0.20828 |
| 1822.342 | 0.26565 | 1822.342 | -0.25925 |
| 1823.306 | 0.25767 | 1823.306 | -0.31956 |
| 1824.271 | 0.2609 | 1824.271 | -0.37594 |
| 1825.235 | 0.27425 | 1825.235 | -0.40032 |
| 1826.199 | 0.29781 | 1826.199 | -0.36363 |
| 1827.163 | 0.32025 | 1827.163 | -0.30992 |
| 1828.128 | 0.33462 | 1828.128 | -0.29325 |
| 1829.092 | 0.34012 | 1829.092 | -0.30748 |
| 1830.056 | 0.3204 | 1830.056 | -0.33713 |
| 1831.02 | 0.27612 | 1831.02 | -0.38735 |
| 1831.984 | 0.24542 | 1831.984 | -0.45263 |
| 1832.949 | 0.24926 | 1832.949 | -0.50919 |
| 1833.913 | 0.27486 | 1833.913 | -0.53347 |
| 1834.877 | 0.30289 | 1834.877 | -0.52245 |
| 1835.841 | 0.33117 | 1835.841 | -0.49927 |
| 1836.806 | 0.36324 | 1836.806 | -0.493 |
| 1837.77 | 0.39162 | 1837.77 | -0.50561 |
| 1838.734 | 0.41259 | 1838.734 | -0.5107 |
| 1839.698 | 0.4336 | 1839.698 | -0.49626 |
| 1840.663 | 0.44961 | 1840.663 | -0.47963 |
| 1841.627 | 0.45012 | 1841.627 | -0.47067 |
| 1842.591 | 0.42952 | 1842.591 | -0.46866 |
| 1843.555 | 0.38728 | 1843.555 | -0.47117 |
| 1844.52 | 0.33808 | 1844.52 | -0.46798 |
| 1845.484 | 0.30093 | 1845.484 | -0.44336 |
| 1846.448 | 0.27751 | 1846.448 | -0.4062 |
| 1847.412 | 0.27381 | 1847.412 | -0.3716 |
| 1848.376 | 0.28491 | 1848.376 | -0.33847 |
| 1849.341 | 0.28737 | 1849.341 | -0.31242 |
| 1850.305 | 0.27744 | 1850.305 | -0.28094 |
| 1851.269 | 0.25847 | 1851.269 | -0.22024 |
| 1852.233 | 0.22961 | 1852.233 | -0.1343 |
| 1853.198 | 0.21694 | 1853.198 | -0.04615 |
| 1854.162 | 0.25727 | 1854.162 | 0.02701 |
| 1855.126 | 0.3408 | 1855.126 | 0.08738 |
| 1856.09 | 0.42814 | 1856.09 | 0.14385 |
| 1857.055 | 0.49203 | 1857.055 | 0.20552 |
| 1858.019 | 0.51293 | 1858.019 | 0.28595 |
| 1858.983 | 0.49699 | 1858.983 | 0.38403 |
| 1859.947 | 0.4837 | 1859.947 | 0.48018 |
| 1860.911 | 0.49192 | 1860.911 | 0.55999 |
| 1861.876 | 0.49215 | 1861.876 | 0.61215 |
| 1862.84 | 0.45336 | 1862.84 | 0.62209 |
| 1863.804 | 0.38858 | 1863.804 | 0.6064 |
| 1864.768 | 0.34318 | 1864.768 | 0.61405 |
| 1865.733 | 0.33862 | 1865.733 | 0.65889 |
| 1866.697 | 0.3419 | 1866.697 | 0.69967 |
| 1867.661 | 0.31654 | 1867.661 | 0.69874 |
| 1868.625 | 0.30004 | 1868.625 | 0.68145 |
| 1869.59 | 0.34041 | 1869.59 | 0.6968 |
| 1870.554 | 0.39421 | 1870.554 | 0.71961 |
| 1871.518 | 0.39627 | 1871.518 | 0.69632 |
| 1872.482 | 0.35878 | 1872.482 | 0.64367 |
| 1873.447 | 0.32329 | 1873.447 | 0.62934 |
| 1874.411 | 0.29741 | 1874.411 | 0.6492 |
| 1875.375 | 0.27921 | 1875.375 | 0.64285 |
| 1876.339 | 0.26766 | 1876.339 | 0.59378 |
| 1877.303 | 0.24726 | 1877.303 | 0.50219 |
| 1878.268 | 0.21943 | 1878.268 | 0.37843 |
| 1879.232 | 0.21511 | 1879.232 | 0.27251 |
| 1880.196 | 0.25239 | 1880.196 | 0.22244 |
| 1881.16 | 0.30424 | 1881.16 | 0.20948 |
| 1882.125 | 0.32576 | 1882.125 | 0.19903 |
| 1883.089 | 0.3265 | 1883.089 | 0.17474 |
| 1884.053 | 0.36343 | 1884.053 | 0.13292 |
| 1885.017 | 0.44221 | 1885.017 | 0.07787 |
| 1885.982 | 0.50497 | 1885.982 | 0.02124 |
| 1886.946 | 0.51668 | 1886.946 | -0.02797 |
| 1887.91 | 0.48679 | 1887.91 | -0.0758 |
| 1888.874 | 0.4323 | 1888.874 | -0.12952 |
| 1889.839 | 0.38185 | 1889.839 | -0.18076 |
| 1890.803 | 0.35963 | 1890.803 | -0.22394 |
| 1891.767 | 0.35528 | 1891.767 | -0.26401 |
| 1892.731 | 0.33509 | 1892.731 | -0.31515 |
| 1893.695 | 0.28855 | 1893.695 | -0.37962 |
| 1894.66 | 0.25665 | 1894.66 | -0.42782 |
| 1895.624 | 0.27119 | 1895.624 | -0.43955 |
| 1896.588 | 0.29124 | 1896.588 | -0.42071 |
| 1897.552 | 0.27525 | 1897.552 | -0.37427 |
| 1898.517 | 0.24068 | 1898.517 | -0.3303 |
| 1899.481 | 0.21007 | 1899.481 | -0.33143 |
| 1900.445 | 0.19923 | 1900.445 | -0.3559 |
| 1901.409 | 0.23492 | 1901.409 | -0.35659 |
| 1902.374 | 0.30398 | 1902.374 | -0.32312 |
| 1903.338 | 0.36016 | 1903.338 | -0.255 |
| 1904.302 | 0.3884 | 1904.302 | -0.16548 |
| 1905.266 | 0.40077 | 1905.266 | -0.09189 |
| 1906.23 | 0.40033 | 1906.23 | -0.05869 |
| 1907.195 | 0.389 | 1907.195 | -0.04917 |
| 1908.159 | 0.3741 | 1908.159 | -0.0396 |
| 1909.123 | 0.34071 | 1909.123 | -0.03711 |
| 1910.087 | 0.26002 | 1910.087 | -0.04887 |
| 1911.052 | 0.15739 | 1911.052 | -0.06569 |
| 1912.016 | 0.08333 | 1912.016 | -0.07861 |
| 1912.98 | 0.0541 | 1912.98 | -0.07405 |
| 1913.944 | 0.05419 | 1913.944 | -0.03931 |
| 1914.909 | 0.05528 | 1914.909 | 0.01236 |
| 1915.873 | 0.0485 | 1915.873 | 0.05737 |
| 1916.837 | 0.04955 | 1916.837 | 0.09324 |
| 1917.801 | 0.03586 | 1917.801 | 0.10105 |
| 1918.766 | -0.03189 | 1918.766 | 0.04441 |
| 1919.73 | -0.12036 | 1919.73 | -0.03326 |
| 1920.694 | -0.16691 | 1920.694 | -0.04344 |
| 1921.658 | -0.16754 | 1921.658 | 0.00477 |
| 1922.622 | -0.15527 | 1922.622 | 0.02524 |
| 1923.587 | -0.14972 | 1923.587 | -0.01546 |
| 1924.551 | -0.13568 | 1924.551 | -0.08399 |
| 1925.515 | -0.09922 | 1925.515 | -0.1513 |
| 1926.479 | -0.07095 | 1926.479 | -0.20513 |
| 1927.444 | -0.08746 | 1927.444 | -0.23653 |
| 1928.408 | -0.12164 | 1928.408 | -0.25004 |
| 1929.372 | -0.12748 | 1929.372 | -0.25763 |
| 1930.336 | -0.10963 | 1930.336 | -0.2702 |
| 1931.301 | -0.08868 | 1931.301 | -0.30049 |
| 1932.265 | -0.08016 | 1932.265 | -0.3479 |
| 1933.229 | -0.11043 | 1933.229 | -0.39795 |
| 1934.193 | -0.18012 | 1934.193 | -0.43782 |
| 1935.157 | -0.24329 | 1935.157 | -0.45529 |
| 1936.122 | -0.26175 | 1936.122 | -0.44134 |
| 1937.086 | -0.23958 | 1937.086 | -0.41007 |
| 1938.05 | -0.19787 | 1938.05 | -0.38225 |
| 1939.014 | -0.15344 | 1939.014 | -0.36699 |
| 1939.979 | -0.116 | 1939.979 | -0.37322 |
| 1940.943 | -0.08667 | 1940.943 | -0.39048 |
| 1941.907 | -0.04776 | 1941.907 | -0.38673 |
| 1942.871 | 0.02282 | 1942.871 | -0.3445 |
| 1943.836 | 0.11472 | 1943.836 | -0.27769 |
| 1944.8 | 0.18945 | 1944.8 | -0.21175 |
| 1945.764 | 0.23743 | 1945.764 | -0.16977 |
| 1946.728 | 0.27648 | 1946.728 | -0.17284 |
| 1947.693 | 0.32729 | 1947.693 | -0.19114 |
| 1948.657 | 0.41431 | 1948.657 | -0.16446 |
| 1949.621 | 0.5153 | 1949.621 | -0.09675 |
| 1950.585 | 0.56063 | 1950.585 | -0.03277 |
| 1951.549 | 0.53597 | 1951.549 | 0.00797 |
| 1952.514 | 0.48372 | 1952.514 | 0.02036 |
| 1953.478 | 0.43117 | 1953.478 | 0.00551 |
| 1954.442 | 0.38817 | 1954.442 | -0.02175 |
| 1955.406 | 0.36296 | 1955.406 | -0.03979 |
| 1956.371 | 0.33761 | 1956.371 | -0.03542 |
| 1957.335 | 0.28597 | 1957.335 | -0.01205 |
| 1958.299 | 0.22985 | 1958.299 | 0.01535 |
| 1959.263 | 0.2241 | 1959.263 | 0.02482 |
| 1960.228 | 0.2642 | 1960.228 | 0.01912 |
| 1961.192 | 0.30641 | 1961.192 | 0.03474 |
| 1962.156 | 0.32835 | 1962.156 | 0.07501 |
| 1963.12 | 0.32238 | 1963.12 | 0.08574 |
| 1964.084 | 0.29192 | 1964.084 | 0.03731 |
| 1965.049 | 0.25725 | 1965.049 | -0.02985 |
| 1966.013 | 0.23835 | 1966.013 | -0.07662 |
| 1966.977 | 0.23736 | 1966.977 | -0.08787 |
| 1967.941 | 0.2378 | 1967.941 | -0.06288 |
| 1968.906 | 0.22558 | 1968.906 | -0.03455 |
| 1969.87 | 0.19187 | 1969.87 | -0.03249 |
| 1970.834 | 0.13556 | 1970.834 | -0.06123 |
| 1971.798 | 0.08209 | 1971.798 | -0.09881 |
| 1972.763 | 0.04053 | 1972.763 | -0.12732 |
| 1973.727 | -0.03709 | 1973.727 | -0.16729 |
| 1974.691 | -0.17809 | 1974.691 | -0.22855 |
| 1975.655 | -0.31641 | 1975.655 | -0.28967 |
| 1976.62 | -0.3733 | 1976.62 | -0.32857 |
| 1977.584 | -0.3742 | 1977.584 | -0.33206 |
| 1978.548 | -0.38833 | 1978.548 | -0.28783 |
| 1979.512 | -0.42812 | 1979.512 | -0.22125 |
| 1980.476 | -0.46095 | 1980.476 | -0.16454 |
| 1981.441 | -0.46749 | 1981.441 | -0.10997 |
| 1982.405 | -0.45638 | 1982.405 | -0.03911 |
| 1983.369 | -0.44287 | 1983.369 | 0.03386 |
| 1984.333 | -0.43429 | 1984.333 | 0.07121 |
| 1985.298 | -0.43134 | 1985.298 | 0.05631 |
| 1986.262 | -0.43392 | 1986.262 | 0.00684 |
| 1987.226 | -0.44005 | 1987.226 | -0.04878 |
| 1988.19 | -0.45076 | 1988.19 | -0.08473 |
| 1989.155 | -0.47387 | 1989.155 | -0.07853 |
| 1990.119 | -0.50142 | 1990.119 | -0.04045 |
| 1991.083 | -0.50801 | 1991.083 | -0.00127 |
| 1992.047 | -0.4965 | 1992.047 | 0.02288 |
| 1993.011 | -0.51693 | 1993.011 | 0.01889 |
| 1993.976 | -0.58471 | 1993.976 | -0.00986 |
| 1994.94 | -0.63125 | 1994.94 | -0.01988 |
| 1995.904 | -0.60677 | 1995.904 | 0.01908 |
| 1996.868 | -0.56038 | 1996.868 | 0.08194 |
| 1997.833 | -0.56266 | 1997.833 | 0.1327 |
| 1998.797 | -0.61411 | 1998.797 | 0.16566 |
| 1999.761 | -0.66001 | 1999.761 | 0.19264 |
| 2000.725 | -0.67361 | 2000.725 | 0.21217 |
| 2001.69 | -0.67824 | 2001.69 | 0.20931 |
| 2002.654 | -0.69112 | 2002.654 | 0.18952 |
| 2003.618 | -0.70678 | 2003.618 | 0.19064 |
| 2004.582 | -0.72191 | 2004.582 | 0.2356 |
| 2005.547 | -0.73701 | 2005.547 | 0.29322 |
| 2006.511 | -0.74931 | 2006.511 | 0.32621 |
| 2007.475 | -0.75654 | 2007.475 | 0.32867 |
| 2008.439 | -0.76765 | 2008.439 | 0.30872 |
| 2009.403 | -0.7827 | 2009.403 | 0.28355 |
| 2010.368 | -0.77343 | 2010.368 | 0.2628 |
| 2011.332 | -0.74381 | 2011.332 | 0.23468 |
| 2012.296 | -0.73994 | 2012.296 | 0.18441 |
| 2013.26 | -0.77345 | 2013.26 | 0.13269 |
| 2014.225 | -0.81197 | 2014.225 | 0.11828 |
| 2015.189 | -0.82811 | 2015.189 | 0.15367 |
| 2016.153 | -0.82017 | 2016.153 | 0.19164 |
| 2017.117 | -0.7916 | 2017.117 | 0.1805 |
| 2018.082 | -0.74616 | 2018.082 | 0.14176 |
| 2019.046 | -0.68711 | 2019.046 | 0.10979 |
| 2020.01 | -0.63309 | 2020.01 | 0.07279 |
| 2020.974 | -0.61592 | 2020.974 | -0.00564 |
| 2021.938 | -0.62591 | 2021.938 | -0.08804 |
| 2022.903 | -0.62472 | 2022.903 | -0.09684 |
|  |  |  |  |

Data for Fig. 6:

| FeOOH/GO-1 | | FeOOH/GO-2 | |
| --- | --- | --- | --- |
| Temperature ℃ | Weight (%) | Temperature ℃ | Weight (%) |
| 29.95 | 99.9793 | 30.476 | 99.9166 |
| 47.749 | 99.2993 | 48.297 | 98.0972 |
| 67.027 | 97.6825 | 67.572 | 96.6544 |
| 86.195 | 95.0138 | 86.742 | 95.7725 |
| 105.342 | 92.6225 | 105.887 | 95.155 |
| 124.478 | 91.3295 | 125.021 | 94.7018 |
| 143.611 | 90.5126 | 144.152 | 94.2866 |
| 162.742 | 89.8433 | 163.286 | 93.8194 |
| 181.877 | 89.157 | 182.42 | 93.2283 |
| 201.012 | 88.3854 | 201.557 | 92.36 |
| 220.152 | 87.4945 | 220.694 | 90.6218 |
| 239.293 | 86.1623 | 239.837 | 87.7652 |
| 258.432 | 83.9427 | 258.98 | 86.9165 |
| 277.581 | 82.9054 | 278.126 | 86.5325 |
| 296.726 | 82.3683 | 297.272 | 86.1326 |
| 320.3146 | 82.02745 | 312.9731 | 85.79796 |
| 350.6384 | 81.32852 | 327.8158 | 85.32258 |
| 379.5258 | 80.18817 | 339.7857 | 84.79626 |
| 405.3808 | 79.02377 | 349.202 | 84.37182 |
| 425.171 | 78.07301 | 357.6607 | 83.89643 |
| 444.9612 | 76.96944 | 367.0771 | 83.52292 |
| 461.3999 | 75.76401 | 374.5782 | 82.9966 |
| 479.275 | 74.17374 | 384.4733 | 82.52122 |
| 500.1824 | 72.11375 | 392.9321 | 82.04584 |
| 517.0999 | 70.10894 | 402.3484 | 81.34975 |
| 533.8577 | 67.4236 | 411.2859 | 80.67063 |
| 551.8924 | 64.95897 | 420.7022 | 79.9236 |
| 577.5878 | 63.59791 | 428.6822 | 79.09168 |
| 616.3703 | 63.24844 | 439.0561 | 78.07301 |
| 651.6416 | 63.13809 | 452.9412 | 76.29032 |
| -- | -- | 465.3899 | 74.74533 |
| -- | -- | 478.7962 | 73.01358 |
| -- | -- | 489.8085 | 71.58744 |
| -- | -- | 501.6188 | 70.11036 |
| -- | -- | 515.0251 | 68.78608 |
| -- | -- | 531.4638 | 67.51273 |
| -- | -- | 553.8076 | 66.4601 |
| -- | -- | 569.1291 | 66.18846 |
| -- | -- | 588.1213 | 65.83192 |
| -- | -- | 608.3903 | 65.71307 |
| -- | -- | 627.223 | 65.61121 |
| -- | -- | 648.6092 | 65.56027 |

Data for Fig. 7:

| FeOOH/GO-1 | | FeOOH/GO-2 | |
| --- | --- | --- | --- |
| Potential (V, vs. Hg/HgO) | Current (A) | Potential (V, vs. Hg/HgO) | Current (A) |
| 0.83301 | 0.12731 | 0.85234 | 0.12558 |
| 0.8324 | 0.12689 | 0.85172 | 0.12517 |
| 0.8324 | 0.12648 | 0.85142 | 0.12476 |
| 0.83179 | 0.12608 | 0.85111 | 0.12434 |
| 0.83117 | 0.12567 | 0.84988 | 0.12393 |
| 0.83056 | 0.12527 | 0.85019 | 0.12352 |
| 0.83025 | 0.12485 | 0.84927 | 0.1231 |
| 0.82995 | 0.12446 | 0.84896 | 0.12268 |
| 0.82964 | 0.12404 | 0.84835 | 0.12226 |
| 0.82903 | 0.12365 | 0.84804 | 0.12184 |
| 0.82841 | 0.12324 | 0.84712 | 0.12144 |
| 0.82811 | 0.12284 | 0.84712 | 0.12102 |
| 0.82749 | 0.12244 | 0.8462 | 0.12055 |
| 0.82657 | 0.12204 | 0.8459 | 0.12006 |
| 0.82688 | 0.12163 | 0.84498 | 0.11965 |
| 0.82627 | 0.12123 | 0.84436 | 0.11926 |
| 0.82596 | 0.12082 | 0.84436 | 0.11886 |
| 0.82535 | 0.12042 | 0.84375 | 0.11845 |
| 0.82412 | 0.12001 | 0.84283 | 0.11803 |
| 0.82443 | 0.11962 | 0.84283 | 0.11765 |
| 0.82412 | 0.11922 | 0.84222 | 0.11722 |
| 0.8232 | 0.11881 | 0.84191 | 0.11682 |
| 0.82351 | 0.11839 | 0.8413 | 0.11642 |
| 0.82259 | 0.11801 | 0.84038 | 0.11601 |
| 0.82197 | 0.1176 | 0.84007 | 0.1156 |
| 0.82136 | 0.11719 | 0.83976 | 0.1152 |
| 0.82044 | 0.1168 | 0.83915 | 0.11479 |
| 0.82044 | 0.1164 | 0.83854 | 0.11438 |
| 0.82013 | 0.11601 | 0.83792 | 0.11396 |
| 0.81952 | 0.11562 | 0.83761 | 0.11354 |
| 0.81891 | 0.11521 | 0.83669 | 0.11316 |
| 0.81799 | 0.11482 | 0.83639 | 0.11276 |
| 0.81799 | 0.11443 | 0.83577 | 0.11236 |
| 0.81768 | 0.11401 | 0.83516 | 0.11195 |
| 0.81737 | 0.11362 | 0.83485 | 0.11155 |
| 0.81707 | 0.11322 | 0.83485 | 0.11114 |
| 0.81676 | 0.11283 | 0.83363 | 0.11074 |
| 0.81584 | 0.11243 | 0.83332 | 0.11032 |
| 0.81492 | 0.11202 | 0.8324 | 0.10993 |
| 0.81492 | 0.11161 | 0.8324 | 0.10954 |
| 0.81431 | 0.11122 | 0.83179 | 0.10913 |
| 0.81369 | 0.11084 | 0.83117 | 0.10872 |
| 0.81339 | 0.11043 | 0.83025 | 0.10831 |
| 0.81277 | 0.11003 | 0.83056 | 0.10791 |
| 0.81216 | 0.10964 | 0.82995 | 0.10752 |
| 0.81185 | 0.10925 | 0.82903 | 0.10712 |
| 0.81154 | 0.10884 | 0.82841 | 0.10672 |
| 0.81093 | 0.10845 | 0.8278 | 0.10631 |
| 0.81032 | 0.10805 | 0.82749 | 0.10591 |
| 0.81032 | 0.10766 | 0.82719 | 0.10551 |
| 0.81001 | 0.10726 | 0.82688 | 0.10511 |
| 0.80878 | 0.10688 | 0.82627 | 0.10472 |
| 0.80848 | 0.10648 | 0.82596 | 0.10432 |
| 0.80817 | 0.10609 | 0.82504 | 0.10394 |
| 0.80786 | 0.1057 | 0.82473 | 0.10353 |
| 0.80725 | 0.10529 | 0.82381 | 0.10314 |
| 0.80664 | 0.10491 | 0.82381 | 0.10274 |
| 0.80602 | 0.10453 | 0.82289 | 0.10235 |
| 0.80572 | 0.10413 | 0.82228 | 0.10194 |
| 0.80541 | 0.10374 | 0.82167 | 0.10154 |
| 0.8048 | 0.10335 | 0.82105 | 0.10116 |
| 0.80449 | 0.10297 | 0.82075 | 0.10077 |
| 0.80357 | 0.10256 | 0.82044 | 0.10036 |
| 0.80296 | 0.10219 | 0.81983 | 0.09997 |
| 0.80265 | 0.1018 | 0.81952 | 0.09958 |
| 0.80234 | 0.1014 | 0.81921 | 0.09917 |
| 0.80173 | 0.101 | 0.81829 | 0.09877 |
| 0.80142 | 0.10062 | 0.81768 | 0.09836 |
| 0.80081 | 0.10023 | 0.81737 | 0.09796 |
| 0.8002 | 0.09983 | 0.81676 | 0.0976 |
| 0.79958 | 0.09944 | 0.81615 | 0.09724 |
| 0.79928 | 0.09906 | 0.81584 | 0.09686 |
| 0.79897 | 0.09867 | 0.81523 | 0.09646 |
| 0.79836 | 0.09827 | 0.81461 | 0.09608 |
| 0.79805 | 0.09788 | 0.81369 | 0.09569 |
| 0.79744 | 0.0975 | 0.81339 | 0.09529 |
| 0.79713 | 0.09712 | 0.81246 | 0.09489 |
| 0.79621 | 0.09672 | 0.81246 | 0.0945 |
| 0.7959 | 0.09634 | 0.81185 | 0.0941 |
| 0.7959 | 0.09595 | 0.81154 | 0.0937 |
| 0.79468 | 0.09555 | 0.81062 | 0.09331 |
| 0.79468 | 0.09518 | 0.81032 | 0.09293 |
| 0.79406 | 0.09477 | 0.81001 | 0.09254 |
| 0.79345 | 0.09439 | 0.80909 | 0.09214 |
| 0.79314 | 0.09401 | 0.80878 | 0.09174 |
| 0.79222 | 0.09362 | 0.80786 | 0.09136 |
| 0.79192 | 0.09322 | 0.80756 | 0.09098 |
| 0.7913 | 0.09285 | 0.80725 | 0.09058 |
| 0.791 | 0.09245 | 0.80602 | 0.0902 |
| 0.79038 | 0.09207 | 0.80572 | 0.0898 |
| 0.79008 | 0.09168 | 0.8051 | 0.0894 |
| 0.78946 | 0.0913 | 0.8048 | 0.08902 |
| 0.78885 | 0.09091 | 0.80418 | 0.08863 |
| 0.78854 | 0.09052 | 0.80388 | 0.08825 |
| 0.78793 | 0.09014 | 0.80357 | 0.08785 |
| 0.78762 | 0.08976 | 0.80234 | 0.08746 |
| 0.78701 | 0.08937 | 0.80204 | 0.08707 |
| 0.7867 | 0.08899 | 0.80142 | 0.08669 |
| 0.78578 | 0.08861 | 0.80081 | 0.0863 |
| 0.78578 | 0.08824 | 0.8005 | 0.08593 |
| 0.78517 | 0.08785 | 0.79989 | 0.08553 |
| 0.78425 | 0.08747 | 0.79897 | 0.08515 |
| 0.78394 | 0.08709 | 0.79866 | 0.08476 |
| 0.78363 | 0.0867 | 0.79836 | 0.08436 |
| 0.78271 | 0.08632 | 0.79774 | 0.08399 |
| 0.7821 | 0.08594 | 0.79744 | 0.08361 |
| 0.7821 | 0.08556 | 0.79652 | 0.08321 |
| 0.78149 | 0.08518 | 0.79621 | 0.08283 |
| 0.78118 | 0.0848 | 0.7956 | 0.08245 |
| 0.78087 | 0.08442 | 0.79498 | 0.08206 |
| 0.77995 | 0.08404 | 0.79376 | 0.08169 |
| 0.77903 | 0.08366 | 0.79376 | 0.08132 |
| 0.77903 | 0.08327 | 0.79284 | 0.08094 |
| 0.77811 | 0.0829 | 0.79314 | 0.08056 |
| 0.77811 | 0.08252 | 0.79253 | 0.08017 |
| 0.7775 | 0.08214 | 0.79161 | 0.07977 |
| 0.77689 | 0.08175 | 0.7913 | 0.07939 |
| 0.77658 | 0.08138 | 0.79038 | 0.07902 |
| 0.77566 | 0.081 | 0.78977 | 0.07863 |
| 0.77566 | 0.08063 | 0.78916 | 0.07825 |
| 0.77535 | 0.08023 | 0.78854 | 0.07788 |
| 0.77443 | 0.07987 | 0.78824 | 0.07749 |
| 0.77413 | 0.07947 | 0.78732 | 0.07711 |
| 0.77321 | 0.07911 | 0.78732 | 0.07673 |
| 0.77259 | 0.07874 | 0.78701 | 0.07635 |
| 0.77229 | 0.07837 | 0.78578 | 0.07598 |
| 0.77198 | 0.07798 | 0.78547 | 0.07559 |
| 0.77137 | 0.0776 | 0.78486 | 0.07521 |
| 0.77075 | 0.07723 | 0.78425 | 0.07483 |
| 0.77045 | 0.07685 | 0.78394 | 0.07445 |
| 0.76983 | 0.07647 | 0.78302 | 0.07407 |
| 0.76983 | 0.0761 | 0.78302 | 0.0737 |
| 0.76861 | 0.07571 | 0.7821 | 0.07332 |
| 0.76861 | 0.07534 | 0.78149 | 0.07295 |
| 0.76769 | 0.07497 | 0.78149 | 0.07257 |
| 0.76769 | 0.07458 | 0.78057 | 0.07217 |
| 0.76738 | 0.07421 | 0.78026 | 0.07179 |
| 0.76615 | 0.07385 | 0.77965 | 0.07142 |
| 0.76554 | 0.07347 | 0.77873 | 0.07103 |
| 0.76523 | 0.0731 | 0.77811 | 0.07067 |
| 0.76462 | 0.07273 | 0.77781 | 0.07028 |
| 0.76431 | 0.07237 | 0.77689 | 0.0699 |
| 0.76401 | 0.07198 | 0.77658 | 0.06953 |
| 0.76339 | 0.07162 | 0.77658 | 0.06916 |
| 0.76278 | 0.07124 | 0.77597 | 0.06879 |
| 0.76217 | 0.07088 | 0.77505 | 0.06843 |
| 0.76186 | 0.07051 | 0.77474 | 0.06806 |
| 0.76094 | 0.07014 | 0.77382 | 0.06767 |
| 0.76094 | 0.06977 | 0.77351 | 0.0673 |
| 0.75971 | 0.0694 | 0.77259 | 0.06692 |
| 0.7594 | 0.06904 | 0.77198 | 0.06656 |
| 0.7591 | 0.06867 | 0.77137 | 0.06619 |
| 0.75848 | 0.0683 | 0.77075 | 0.06582 |
| 0.75818 | 0.06792 | 0.77045 | 0.06545 |
| 0.75756 | 0.06757 | 0.77045 | 0.06507 |
| 0.75695 | 0.06719 | 0.76922 | 0.06469 |
| 0.75634 | 0.06683 | 0.76891 | 0.06432 |
| 0.75572 | 0.06646 | 0.7683 | 0.06397 |
| 0.75511 | 0.06608 | 0.76738 | 0.06359 |
| 0.7548 | 0.06573 | 0.76738 | 0.06322 |
| 0.75388 | 0.06537 | 0.76646 | 0.06285 |
| 0.75419 | 0.065 | 0.76585 | 0.06251 |
| 0.75358 | 0.06463 | 0.76523 | 0.06213 |
| 0.75266 | 0.06426 | 0.76462 | 0.06176 |
| 0.75266 | 0.06389 | 0.76431 | 0.0614 |
| 0.75204 | 0.06353 | 0.76309 | 0.06103 |
| 0.75112 | 0.06317 | 0.76278 | 0.06067 |
| 0.75051 | 0.06281 | 0.76217 | 0.06029 |
| 0.7502 | 0.06244 | 0.76247 | 0.05993 |
| 0.7499 | 0.0621 | 0.76125 | 0.05957 |
| 0.74898 | 0.06172 | 0.76063 | 0.0592 |
| 0.74867 | 0.06135 | 0.76032 | 0.05884 |
| 0.74836 | 0.061 | 0.75971 | 0.05848 |
| 0.74744 | 0.06063 | 0.75879 | 0.0581 |
| 0.74744 | 0.06026 | 0.75879 | 0.05775 |
| 0.74652 | 0.05991 | 0.75848 | 0.0574 |
| 0.74622 | 0.05954 | 0.75726 | 0.05702 |
| 0.7453 | 0.05918 | 0.75664 | 0.05667 |
| 0.74499 | 0.05882 | 0.75634 | 0.05631 |
| 0.74438 | 0.05847 | 0.75542 | 0.05597 |
| 0.74376 | 0.0581 | 0.7548 | 0.05559 |
| 0.74376 | 0.05775 | 0.7548 | 0.05523 |
| 0.74284 | 0.05738 | 0.75388 | 0.05488 |
| 0.74223 | 0.05702 | 0.75327 | 0.05451 |
| 0.74162 | 0.05667 | 0.75296 | 0.05415 |
| 0.74131 | 0.05631 | 0.75235 | 0.05378 |
| 0.7407 | 0.05593 | 0.75143 | 0.05343 |
| 0.74008 | 0.05561 | 0.75112 | 0.05308 |
| 0.73947 | 0.05523 | 0.7499 | 0.05272 |
| 0.73916 | 0.05488 | 0.74959 | 0.05237 |
| 0.73855 | 0.05453 | 0.74898 | 0.05202 |
| 0.73794 | 0.05417 | 0.74898 | 0.05165 |
| 0.73763 | 0.05381 | 0.74775 | 0.05129 |
| 0.73732 | 0.05345 | 0.74775 | 0.05094 |
| 0.73702 | 0.0531 | 0.74683 | 0.05058 |
| 0.73579 | 0.05275 | 0.74622 | 0.05023 |
| 0.73548 | 0.0524 | 0.7456 | 0.04989 |
| 0.73487 | 0.05205 | 0.7453 | 0.04953 |
| 0.73425 | 0.05168 | 0.74438 | 0.04918 |
| 0.73364 | 0.05134 | 0.74376 | 0.04883 |
| 0.73303 | 0.05099 | 0.74346 | 0.04848 |
| 0.73272 | 0.05064 | 0.74254 | 0.04814 |
| 0.7318 | 0.05028 | 0.74192 | 0.04779 |
| 0.7318 | 0.04993 | 0.74192 | 0.04745 |
| 0.73119 | 0.04959 | 0.74131 | 0.0471 |
| 0.73027 | 0.04923 | 0.74008 | 0.04676 |
| 0.72996 | 0.04888 | 0.73978 | 0.0464 |
| 0.72935 | 0.04854 | 0.73886 | 0.04607 |
| 0.72873 | 0.0482 | 0.73886 | 0.04572 |
| 0.72843 | 0.04785 | 0.73794 | 0.04537 |
| 0.72751 | 0.0475 | 0.73763 | 0.04502 |
| 0.7272 | 0.04716 | 0.73671 | 0.04467 |
| 0.7272 | 0.04681 | 0.7364 | 0.04434 |
| 0.72597 | 0.04648 | 0.73548 | 0.04398 |
| 0.72597 | 0.04612 | 0.73487 | 0.04363 |
| 0.72505 | 0.04579 | 0.73425 | 0.0433 |
| 0.72475 | 0.04544 | 0.73364 | 0.04296 |
| 0.72383 | 0.04509 | 0.73333 | 0.04262 |
| 0.72321 | 0.04475 | 0.73272 | 0.04225 |
| 0.7226 | 0.04441 | 0.73211 | 0.04193 |
| 0.72229 | 0.04407 | 0.73088 | 0.0416 |
| 0.72199 | 0.04372 | 0.73119 | 0.04126 |
| 0.72137 | 0.04338 | 0.73057 | 0.04091 |
| 0.72076 | 0.04305 | 0.72935 | 0.04058 |
| 0.71984 | 0.04272 | 0.72904 | 0.04025 |
| 0.71953 | 0.04237 | 0.72843 | 0.0399 |
| 0.71892 | 0.04202 | 0.72751 | 0.03957 |
| 0.71831 | 0.04169 | 0.72689 | 0.03925 |
| 0.718 | 0.04136 | 0.72659 | 0.0389 |
| 0.71677 | 0.04102 | 0.72597 | 0.03858 |
| 0.71677 | 0.04069 | 0.72505 | 0.03823 |
| 0.71647 | 0.04036 | 0.72413 | 0.03758 |
| 0.71585 | 0.04002 | 0.72352 | 0.03723 |
| 0.71493 | 0.0397 | 0.7226 | 0.03692 |
| 0.71401 | 0.03935 | 0.7226 | 0.03658 |
| 0.7134 | 0.03903 | 0.72168 | 0.03625 |
| 0.71309 | 0.03869 | 0.72107 | 0.03593 |
| 0.71279 | 0.03836 | 0.72076 | 0.03558 |
| 0.71187 | 0.03803 | 0.71953 | 0.03526 |
| 0.71187 | 0.03769 | 0.71953 | 0.03494 |
| 0.71125 | 0.03738 | 0.71861 | 0.03462 |
| 0.71033 | 0.03704 | 0.718 | 0.0343 |
| 0.71003 | 0.03671 | 0.71739 | 0.03397 |
| 0.7088 | 0.03641 | 0.71616 | 0.03365 |
| 0.70849 | 0.03606 | 0.71647 | 0.03331 |
| 0.70818 | 0.03574 | 0.71493 | 0.033 |
| 0.70757 | 0.03542 | 0.71432 | 0.03269 |
| 0.70696 | 0.03508 | 0.71432 | 0.03236 |
| 0.70604 | 0.03477 | 0.7134 | 0.03204 |
| 0.70542 | 0.03446 | 0.71309 | 0.03172 |
| 0.70542 | 0.03411 | 0.71217 | 0.03142 |
| 0.7045 | 0.0338 | 0.71125 | 0.03111 |
| 0.7045 | 0.03348 | 0.71125 | 0.03077 |
| 0.70358 | 0.03316 | 0.71033 | 0.03047 |
| 0.70328 | 0.03283 | 0.70972 | 0.03017 |
| 0.70297 | 0.03253 | 0.7088 | 0.02984 |
| 0.70174 | 0.03222 | 0.70818 | 0.02954 |
| 0.70082 | 0.03187 | 0.70757 | 0.02921 |
| 0.70021 | 0.03157 | 0.70726 | 0.0289 |
| 0.70021 | 0.03127 | 0.70634 | 0.02859 |
| 0.6996 | 0.03092 | 0.70573 | 0.02828 |
| 0.69837 | 0.03063 | 0.70481 | 0.02797 |
| 0.69776 | 0.03031 | 0.7042 | 0.02768 |
| 0.69776 | 0.03 | 0.70389 | 0.02736 |
| 0.69684 | 0.02969 | 0.70328 | 0.02706 |
| 0.69622 | 0.02939 | 0.70297 | 0.02674 |
| 0.69622 | 0.02907 | 0.70205 | 0.02644 |
| 0.69469 | 0.02847 | 0.70113 | 0.02615 |
| 0.69408 | 0.02816 | 0.70082 | 0.02584 |
| 0.69346 | 0.02785 | 0.6999 | 0.02555 |
| 0.69285 | 0.02755 | 0.6996 | 0.02525 |
| 0.69254 | 0.02724 | 0.69868 | 0.02495 |
| 0.69162 | 0.02693 | 0.69806 | 0.02465 |
| 0.69132 | 0.02664 | 0.69745 | 0.02436 |
| 0.6907 | 0.02634 | 0.69653 | 0.02406 |
| 0.68978 | 0.02603 | 0.69592 | 0.02378 |
| 0.68978 | 0.02573 | 0.69592 | 0.0235 |
| 0.68886 | 0.02544 | 0.695 | 0.02319 |
| 0.68794 | 0.02515 | 0.69438 | 0.02289 |
| 0.68733 | 0.02486 | 0.69316 | 0.0226 |
| 0.68672 | 0.02455 | 0.69254 | 0.02231 |
| 0.6858 | 0.02425 | 0.69254 | 0.02204 |
| 0.68549 | 0.02397 | 0.69132 | 0.02176 |
| 0.68488 | 0.02368 | 0.6907 | 0.02146 |
| 0.68426 | 0.02338 | 0.69009 | 0.02119 |
| 0.68365 | 0.0231 | 0.68948 | 0.02091 |
| 0.68273 | 0.0228 | 0.68917 | 0.02062 |
| 0.68242 | 0.02252 | 0.68764 | 0.02035 |
| 0.6815 | 0.02224 | 0.68702 | 0.02007 |
| 0.68119 | 0.02196 | 0.68641 | 0.01979 |
| 0.68058 | 0.02165 | 0.68549 | 0.01952 |
| 0.67966 | 0.02138 | 0.68549 | 0.01924 |
| 0.67905 | 0.0211 | 0.68426 | 0.01897 |
| 0.67843 | 0.02083 | 0.68396 | 0.0187 |
| 0.67813 | 0.02053 | 0.68303 | 0.01841 |
| 0.67721 | 0.02027 | 0.68273 | 0.01816 |
| 0.67659 | 0.01998 | 0.6815 | 0.01789 |
| 0.67598 | 0.01972 | 0.68089 | 0.01762 |
| 0.67567 | 0.01945 | 0.68027 | 0.01734 |
| 0.67506 | 0.01917 | 0.67966 | 0.01708 |
| 0.67383 | 0.0189 | 0.67935 | 0.01683 |
| 0.67322 | 0.01862 | 0.67813 | 0.01657 |
| 0.67291 | 0.01835 | 0.67751 | 0.01629 |
| 0.67199 | 0.01808 | 0.6769 | 0.01603 |
| 0.67169 | 0.01783 | 0.67598 | 0.0158 |
| 0.67107 | 0.01756 | 0.67537 | 0.01553 |
| 0.67046 | 0.01729 | 0.67445 | 0.01527 |
| 0.66954 | 0.01703 | 0.67414 | 0.01503 |
| 0.66893 | 0.01677 | 0.67322 | 0.01478 |
| 0.66831 | 0.01651 | 0.67261 | 0.01452 |
| 0.66739 | 0.01625 | 0.67199 | 0.01429 |
| 0.66709 | 0.016 | 0.67138 | 0.01403 |
| 0.66647 | 0.01573 | 0.67138 | 0.01408 |
| 0.66586 | 0.01549 | 0.66985 | 0.01379 |
| 0.66494 | 0.01523 | 0.66954 | 0.01353 |
| 0.66433 | 0.01497 | 0.66862 | 0.01328 |
| 0.6631 | 0.01474 | 0.66801 | 0.01303 |
| 0.66249 | 0.01449 | 0.66709 | 0.01279 |
| 0.66249 | 0.01424 | 0.66647 | 0.01254 |
| 0.66187 | 0.01398 | 0.66586 | 0.01231 |
| 0.66126 | 0.01406 | 0.66525 | 0.01206 |
| 0.66034 | 0.01376 | 0.66463 | 0.01183 |
| 0.65942 | 0.0135 | 0.66341 | 0.01159 |
| 0.65942 | 0.01325 | 0.66279 | 0.01136 |
| 0.6585 | 0.01301 | 0.66218 | 0.01113 |
| 0.65788 | 0.01276 | 0.66095 | 0.0109 |
| 0.65696 | 0.01252 | 0.66126 | 0.01067 |
| 0.65666 | 0.01228 | 0.66003 | 0.01044 |
| 0.65604 | 0.01204 | 0.65911 | 0.01023 |
| 0.65543 | 0.01181 | 0.65819 | 0.01 |
| 0.6539 | 0.01158 | 0.65788 | 0.00977 |
| 0.65359 | 0.01135 | 0.65727 | 0.00956 |
| 0.65298 | 0.01111 | 0.65635 | 0.00934 |
| 0.65206 | 0.01089 | 0.65543 | 0.00913 |
| 0.65114 | 0.01066 | 0.6542 | 0.00891 |
| 0.65052 | 0.01044 | 0.65359 | 0.0087 |
| 0.6496 | 0.01021 | 0.65298 | 0.00849 |
| 0.64899 | 0.00999 | 0.65236 | 0.00827 |
| 0.64838 | 0.00977 | 0.65175 | 0.00807 |
| 0.64776 | 0.00955 | 0.65083 | 0.00786 |
| 0.64715 | 0.00934 | 0.64991 | 0.00766 |
| 0.64623 | 0.00912 | 0.6493 | 0.00746 |
| 0.64531 | 0.00891 | 0.64807 | 0.00726 |
| 0.645 | 0.0087 | 0.64746 | 0.00706 |
| 0.64378 | 0.00849 | 0.64684 | 0.00687 |
| 0.64286 | 0.00829 | 0.64623 | 0.00667 |
| 0.64286 | 0.00808 | 0.64531 | 0.00648 |
| 0.64224 | 0.00788 | 0.64439 | 0.00629 |
| 0.64071 | 0.00768 | 0.64408 | 0.00609 |
| 0.64071 | 0.00748 | 0.64286 | 0.0059 |
| 0.6401 | 0.00728 | 0.64224 | 0.00571 |
| 0.63856 | 0.00709 | 0.64102 | 0.00553 |
| 0.63795 | 0.00689 | 0.64071 | 0.00535 |
| 0.63795 | 0.0067 | 0.63887 | 0.00499 |
| 0.63672 | 0.00651 | 0.63795 | 0.00482 |
| 0.6358 | 0.00632 | 0.63764 | 0.00464 |
| 0.6355 | 0.00614 | 0.63672 | 0.00446 |
| 0.63396 | 0.00595 | 0.6358 | 0.00429 |
| 0.63335 | 0.00577 | 0.63519 | 0.00412 |
| 0.63274 | 0.00559 | 0.63366 | 0.00395 |
| 0.63243 | 0.00541 | 0.63366 | 0.00378 |
| 0.6312 | 0.00523 | 0.63243 | 0.00362 |
| 0.63059 | 0.00505 | 0.63151 | 0.00345 |
| 0.62936 | 0.00488 | 0.63089 | 0.0033 |
| 0.62844 | 0.00471 | 0.62967 | 0.00314 |
| 0.62783 | 0.00454 | 0.62905 | 0.00298 |
| 0.62752 | 0.00437 | 0.62813 | 0.00282 |
| 0.6266 | 0.0042 | 0.62721 | 0.00266 |
| 0.62568 | 0.00404 | 0.62691 | 0.00251 |
| 0.62537 | 0.00388 | 0.62599 | 0.00236 |
| 0.62445 | 0.00372 | 0.62507 | 0.00221 |
| 0.62292 | 0.00356 | 0.62384 | 0.00206 |
| 0.62231 | 0.0034 | 0.62353 | 0.00192 |
| 0.62169 | 0.00325 | 0.62231 | 0.00177 |
| 0.62047 | 0.00309 | 0.622 | 0.00163 |
| 0.61985 | 0.00294 | 0.62077 | 0.00149 |
| 0.61955 | 0.00279 | 0.61955 | 0.00134 |
| 0.61863 | 0.00264 | 0.61893 | 0.00121 |
| 0.61771 | 0.00249 | 0.61801 | 0.00107 |
| 0.61679 | 0.00234 | 0.61771 | 9.35E-04 |
| 0.61617 | 0.00221 | 0.61648 | 7.99E-04 |
| 0.61495 | 0.00206 | 0.61556 | 6.72E-04 |
| 0.61464 | 0.00192 | 0.61495 | 5.34E-04 |
| 0.61372 | 0.00178 | 0.61403 | 4.09E-04 |
| 0.61249 | 0.00164 | 0.61341 | 2.79E-04 |
| 0.61188 | 0.00151 | 0.61219 | 1.58E-04 |
| 0.61065 | 0.00138 | 0.61157 | 3.01E-05 |
| 0.61035 | 0.00124 | 0.61035 | -8.91E-05 |
| 0.60943 | 0.00111 | 0.60973 | -2.01E-04 |
| 0.60728 | 8.56E-04 | 0.60881 | -3.38E-04 |
| 0.60667 | 7.27E-04 | 0.60789 | -4.55E-04 |
| 0.60605 | 6.00E-04 | 0.60697 | -5.72E-04 |
| 0.60513 | 4.79E-04 | 0.60605 | -6.92E-04 |
| 0.60452 | 3.60E-04 | 0.60513 | -8.06E-04 |
| 0.6039 | 2.30E-04 | 0.6039 | -9.23E-04 |
| 0.60268 | 1.22E-04 | 0.6036 | -0.00103 |
| 0.60176 | 3.80E-07 | 0.60176 | -0.00114 |
| 0.60084 | -2.01E-06 | 0.60176 | -0.00125 |
| 0.60022 | -2.01E-05 | 0.60084 | -0.00136 |
| 0.5993 | -2.01E-04 | 0.59992 | -0.00147 |
| 0.59808 | -4.81E-04 | 0.5993 | -0.00158 |
| 0.59716 | -5.84E-04 | 0.59838 | -0.00168 |
| 0.59654 | -6.91E-04 | 0.59777 | -0.00179 |
| 0.59593 | -7.99E-04 | 0.59624 | -0.00189 |
| 0.59501 | -9.01E-04 | 0.59562 | -0.00199 |
| 0.59409 | -0.00101 | 0.5947 | -0.0021 |
| 0.59286 | -0.00111 | 0.59378 | -0.00219 |
| 0.59194 | -0.00122 | 0.59256 | -0.00229 |
| 0.59102 | -0.00132 | 0.59194 | -0.00239 |
| 0.59041 | -0.00142 | 0.59102 | -0.00249 |
| 0.58918 | -0.00152 | 0.5898 | -0.00258 |
| 0.58857 | -0.00162 | 0.58949 | -0.00268 |
| 0.58796 | -0.00172 | 0.58857 | -0.00277 |
| 0.58704 | -0.00182 | 0.58734 | -0.00286 |
| 0.58581 | -0.00191 | 0.58612 | -0.00295 |
| 0.5852 | -0.00201 | 0.58581 | -0.00304 |
| 0.58458 | -0.00211 | 0.58489 | -0.00313 |
| 0.58336 | -0.00219 | 0.58397 | -0.00322 |
| 0.58244 | -0.00229 | 0.58274 | -0.00331 |
| 0.58182 | -0.00238 | 0.58213 | -0.00339 |
| 0.58059 | -0.00247 | 0.58152 | -0.00347 |
| 0.57998 | -0.00256 | 0.58029 | -0.00356 |
| 0.57937 | -0.00265 | 0.57906 | -0.00364 |
| 0.57814 | -0.00273 | 0.57845 | -0.00373 |
| 0.57722 | -0.00282 | 0.57783 | -0.00381 |
| 0.57599 | -0.00291 | 0.5763 | -0.00389 |
| 0.57599 | -0.00299 | 0.57569 | -0.00397 |
| 0.57446 | -0.00308 | 0.57477 | -0.00405 |
| 0.57354 | -0.00316 | 0.57415 | -0.00412 |
| 0.57262 | -0.00324 | 0.57323 | -0.0042 |
| 0.5717 | -0.00332 | 0.57201 | -0.00428 |
| 0.57109 | -0.0034 | 0.57109 | -0.00436 |
| 0.57047 | -0.00349 | 0.57047 | -0.00443 |
| 0.56925 | -0.00356 | 0.56925 | -0.00451 |
| 0.56833 | -0.00364 | 0.56833 | -0.00458 |
| 0.56741 | -0.00372 | 0.56741 | -0.00466 |
| 0.56618 | -0.0038 | 0.56618 | -0.00473 |
| 0.56587 | -0.00387 | 0.56587 | -0.0048 |
| 0.56403 | -0.00395 | 0.56495 | -0.00487 |
| 0.56403 | -0.00403 | 0.56373 | -0.00494 |
| 0.56281 | -0.0041 | 0.56311 | -0.00501 |
| 0.56158 | -0.00417 | 0.56158 | -0.00508 |
| 0.56097 | -0.00425 | 0.56097 | -0.00515 |
| 0.56005 | -0.00432 | 0.56005 | -0.00522 |
| 0.55943 | -0.00439 | 0.55882 | -0.00529 |
| 0.55821 | -0.00446 | 0.5579 | -0.00535 |
| 0.55729 | -0.00453 | 0.55729 | -0.00541 |
| 0.55637 | -0.0046 | 0.55606 | -0.00548 |
| 0.55545 | -0.00468 | 0.55545 | -0.00554 |
| 0.55483 | -0.00474 | 0.55452 | -0.00561 |
| 0.55422 | -0.00481 | 0.5533 | -0.00568 |
| 0.55268 | -0.00488 | 0.55238 | -0.00574 |
| 0.55176 | -0.00495 | 0.55115 | -0.0058 |
| 0.55084 | -0.00501 | 0.55054 | -0.00586 |
| 0.54992 | -0.00508 | 0.54992 | -0.00592 |
| 0.549 | -0.00515 | 0.549 | -0.00598 |
| 0.54839 | -0.00522 | 0.54778 | -0.00605 |
| 0.54686 | -0.00528 | 0.54686 | -0.00611 |
| 0.54655 | -0.00535 | 0.54594 | -0.00617 |
| 0.54563 | -0.00541 | 0.54502 | -0.00623 |
| 0.54471 | -0.00547 | 0.54379 | -0.00629 |
| 0.54348 | -0.00553 | 0.54318 | -0.00635 |
| 0.54256 | -0.00561 | 0.54226 | -0.0064 |
| 0.54164 | -0.00566 | 0.54103 | -0.00646 |
| 0.54103 | -0.00573 | 0.54042 | -0.00651 |
| 0.54011 | -0.00579 | 0.53919 | -0.00657 |
| 0.53888 | -0.00586 | 0.53827 | -0.00663 |
| 0.53827 | -0.00591 | 0.53766 | -0.00668 |
| 0.53735 | -0.00597 | 0.53674 | -0.00674 |
| 0.53643 | -0.00603 | 0.53551 | -0.00679 |
| 0.5349 | -0.00609 | 0.5349 | -0.00686 |
| 0.53428 | -0.00615 | 0.53398 | -0.00689 |
| 0.53336 | -0.00621 | 0.53306 | -0.00695 |
| 0.53244 | -0.00627 | 0.53183 | -0.00701 |
| 0.53122 | -0.00633 | 0.5306 | -0.00706 |
| 0.5306 | -0.00639 | 0.5303 | -0.00711 |
| 0.52968 | -0.00645 | 0.52938 | -0.00716 |
| 0.52876 | -0.0065 | 0.52815 | -0.00721 |
| 0.52784 | -0.00657 | 0.52723 | -0.00727 |
| 0.52692 | -0.00662 | 0.52569 | -0.00732 |
| 0.526 | -0.00668 | 0.52539 | -0.00737 |
| 0.52539 | -0.00673 | 0.52477 | -0.00742 |
| 0.52447 | -0.00679 | 0.52324 | -0.00748 |
| 0.52355 | -0.00685 | 0.52263 | -0.00752 |
| 0.52263 | -0.0069 | 0.5214 | -0.00757 |
| 0.52109 | -0.00696 | 0.52048 | -0.00762 |
| 0.52079 | -0.00701 | 0.51956 | -0.00767 |
| 0.51925 | -0.00707 | 0.51864 | -0.00772 |
| 0.51864 | -0.00712 | 0.51772 | -0.00776 |
| 0.51772 | -0.00718 | 0.5168 | -0.00781 |
| 0.5168 | -0.00724 | 0.51588 | -0.00786 |
| 0.51619 | -0.00729 | 0.51465 | -0.00791 |
| 0.51496 | -0.00734 | 0.51373 | -0.00796 |
| 0.51373 | -0.0074 | 0.51281 | -0.008 |
| 0.51312 | -0.00746 | 0.51189 | -0.00805 |
| 0.51189 | -0.00751 | 0.51067 | -0.0081 |
| 0.51159 | -0.00757 | 0.51036 | -0.00814 |
| 0.51067 | -0.00762 | 0.50944 | -0.00819 |
| 0.50944 | -0.00768 | 0.50821 | -0.00824 |
| 0.50821 | -0.00773 | 0.50699 | -0.00828 |
| 0.50729 | -0.00779 | 0.50668 | -0.00833 |
| 0.50668 | -0.00784 | 0.50515 | -0.00838 |
| 0.50545 | -0.0079 | 0.50484 | -0.00843 |
| 0.50484 | -0.00795 | 0.503 | -0.00847 |
| 0.50392 | -0.00801 | 0.50238 | -0.00852 |
| 0.50269 | -0.00807 | 0.50116 | -0.00857 |
| 0.50208 | -0.00812 | 0.50085 | -0.00862 |
| 0.50085 | -0.00817 | 0.49962 | -0.00867 |
| 0.50024 | -0.00823 | 0.4987 | -0.00871 |
| 0.49932 | -0.00829 | 0.49778 | -0.00876 |
| 0.4984 | -0.00835 | 0.49686 | -0.0088 |
| 0.49686 | -0.0084 | 0.49533 | -0.00885 |
| 0.49594 | -0.00846 | 0.49502 | -0.0089 |
| 0.49502 | -0.00852 | 0.4938 | -0.00894 |
| 0.4941 | -0.00858 | 0.49349 | -0.00899 |
| 0.49318 | -0.00863 | 0.49226 | -0.00904 |
| 0.49257 | -0.0087 | 0.49104 | -0.00909 |
| 0.49165 | -0.00875 | 0.49012 | -0.00914 |
| 0.49073 | -0.00881 | 0.4892 | -0.00918 |
| 0.48981 | -0.00888 | 0.48858 | -0.00923 |
| 0.48858 | -0.00894 | 0.48736 | -0.00928 |
| 0.48797 | -0.009 | 0.48644 | -0.00933 |
| 0.48705 | -0.00906 | 0.48552 | -0.00938 |
| 0.48613 | -0.00912 | 0.4846 | -0.00944 |
| 0.48521 | -0.00918 | 0.48306 | -0.00948 |
| 0.48429 | -0.00925 | 0.48276 | -0.00953 |
| 0.48337 | -0.00931 | 0.48184 | -0.00959 |
| 0.48245 | -0.00938 | 0.48092 | -0.00963 |
| 0.48153 | -0.00944 | 0.47969 | -0.00969 |
| 0.48061 | -0.0095 | 0.47908 | -0.00975 |
| 0.47969 | -0.00958 | 0.47754 | -0.0098 |
| 0.47877 | -0.00964 | 0.47723 | -0.00985 |
| 0.47816 | -0.00971 | 0.47601 | -0.00991 |
| 0.47723 | -0.00978 | 0.47509 | -0.00996 |
| 0.47662 | -0.00985 | 0.47417 | -0.01002 |
| 0.47478 | -0.00992 | 0.47325 | -0.01007 |
| 0.47447 | -0.01 | 0.47233 | -0.01013 |
| 0.47355 | -0.01007 | 0.47141 | -0.01018 |
| 0.47233 | -0.01014 | 0.47018 | -0.01025 |
| 0.47141 | -0.01021 | 0.46926 | -0.01031 |
| 0.47049 | -0.01029 | 0.46895 | -0.01037 |
| 0.46987 | -0.01037 | 0.46711 | -0.01043 |
| 0.46865 | -0.01044 | 0.46681 | -0.01049 |
| 0.46803 | -0.01052 | 0.46589 | -0.01056 |
| 0.46711 | -0.01061 | 0.46497 | -0.01062 |
| 0.46619 | -0.01069 | 0.46374 | -0.01069 |
| 0.46497 | -0.01077 | 0.46343 | -0.01076 |
| 0.46435 | -0.01085 | 0.4619 | -0.01083 |
| 0.46343 | -0.01094 | 0.46098 | -0.0109 |
| 0.46221 | -0.01102 | 0.46037 | -0.01096 |
| 0.46159 | -0.01111 | 0.45975 | -0.01104 |
| 0.46098 | -0.0112 | 0.45822 | -0.01111 |
| 0.45975 | -0.01128 | 0.45761 | -0.01119 |
| 0.45914 | -0.01138 | 0.45669 | -0.01127 |
| 0.45791 | -0.01147 | 0.45577 | -0.01135 |
| 0.45699 | -0.01157 | 0.45454 | -0.01143 |
| 0.45607 | -0.01165 | 0.45393 | -0.01151 |
| 0.45546 | -0.01174 | 0.45301 | -0.0116 |
| 0.45423 | -0.01185 | 0.45209 | -0.01168 |
| 0.45362 | -0.01194 | 0.45116 | -0.01177 |
| 0.45331 | -0.01204 | 0.45024 | -0.01186 |
| 0.45209 | -0.01213 | 0.44932 | -0.01195 |
| 0.45086 | -0.01224 | 0.4484 | -0.01205 |
| 0.45024 | -0.01234 | 0.44748 | -0.01214 |
| 0.44902 | -0.01244 | 0.44656 | -0.01224 |
| 0.4484 | -0.01254 | 0.44564 | -0.01234 |
| 0.44779 | -0.01264 | 0.44472 | -0.01245 |
| 0.44687 | -0.01274 | 0.44442 | -0.01255 |
| 0.44564 | -0.01285 | 0.44319 | -0.01267 |
| 0.44472 | -0.01294 | 0.44135 | -0.01277 |
| 0.4435 | -0.01305 | 0.44135 | -0.01289 |
| 0.44319 | -0.01316 | 0.44043 | -0.013 |
| 0.44227 | -0.01326 | 0.43982 | -0.01311 |
| 0.44135 | -0.01336 | 0.4389 | -0.01324 |
| 0.44074 | -0.01346 | 0.43798 | -0.01336 |
| 0.43951 | -0.01357 | 0.43675 | -0.01349 |
| 0.4389 | -0.01367 | 0.43614 | -0.01361 |
| 0.43798 | -0.01377 | 0.43522 | -0.01374 |
| 0.43736 | -0.01386 | 0.43491 | -0.01387 |
| 0.43614 | -0.01396 | 0.43338 | -0.014 |
| 0.43522 | -0.01406 | 0.43276 | -0.01413 |
| 0.43368 | -0.01415 | 0.43154 | -0.01427 |
| 0.43338 | -0.01425 | 0.43092 | -0.0144 |
| 0.43276 | -0.01434 | 0.43 | -0.01455 |
| 0.43184 | -0.01444 | 0.42939 | -0.01469 |
| 0.43062 | -0.01453 | 0.42816 | -0.01483 |
| 0.4297 | -0.01462 | 0.42755 | -0.01498 |
| 0.42878 | -0.0147 | 0.42694 | -0.01511 |
| 0.42816 | -0.01479 | 0.42601 | -0.01527 |
| 0.42724 | -0.01487 | 0.42479 | -0.01541 |
| 0.42632 | -0.01495 | 0.42417 | -0.01555 |
| 0.42509 | -0.01502 | 0.42325 | -0.0157 |
| 0.42448 | -0.0151 | 0.42264 | -0.01585 |
| 0.42417 | -0.01517 | 0.42141 | -0.016 |
| 0.42264 | -0.01524 | 0.42111 | -0.01615 |
| 0.42203 | -0.0153 | 0.41988 | -0.01629 |
| 0.42111 | -0.01537 | 0.41896 | -0.01643 |
| 0.42019 | -0.01543 | 0.41835 | -0.01658 |
| 0.41927 | -0.01548 | 0.41773 | -0.01673 |
| 0.41835 | -0.01554 | 0.41681 | -0.01688 |
| 0.41743 | -0.01559 | 0.4162 | -0.01702 |
| 0.41681 | -0.01564 | 0.41497 | -0.01716 |
| 0.41528 | -0.01568 | 0.41405 | -0.01731 |
| 0.41405 | -0.01573 | 0.41344 | -0.01745 |
| 0.41344 | -0.01577 | 0.41252 | -0.01759 |
| 0.41283 | -0.0158 | 0.41191 | -0.01773 |
| 0.4116 | -0.01584 | 0.41099 | -0.01787 |
| 0.41007 | -0.01586 | 0.41007 | -0.01799 |
| 0.40976 | -0.01589 | 0.40884 | -0.01813 |
| 0.40853 | -0.01592 | 0.40884 | -0.0178 |
| 0.40761 | -0.01594 | 0.40792 | -0.01794 |
| 0.40669 | -0.01596 | 0.40731 | -0.01808 |
| 0.40547 | -0.01598 | 0.40639 | -0.01821 |
| 0.40485 | -0.01599 | 0.40547 | -0.01834 |
| 0.40363 | -0.016 | 0.40455 | -0.01848 |
| 0.4024 | -0.01601 | 0.40393 | -0.0186 |
| 0.40209 | -0.01602 | 0.40271 | -0.01872 |
| 0.40087 | -0.01603 | 0.40179 | -0.01886 |
| 0.39964 | -0.01604 | 0.40087 | -0.01897 |
| 0.39902 | -0.01604 | 0.39994 | -0.01908 |
| 0.3978 | -0.01604 | 0.39933 | -0.01921 |
| 0.39688 | -0.01604 | 0.39841 | -0.01933 |
| 0.39565 | -0.01605 | 0.39749 | -0.01942 |
| 0.39473 | -0.01605 | 0.39657 | -0.01954 |
| 0.39412 | -0.01605 | 0.39596 | -0.01964 |
| 0.39289 | -0.01605 | 0.39473 | -0.01975 |
| 0.39166 | -0.01605 | 0.39381 | -0.01984 |
| 0.39074 | -0.01604 | 0.3932 | -0.01994 |
| 0.39013 | -0.01604 | 0.39228 | -0.02004 |
| 0.3886 | -0.01603 | 0.39105 | -0.02014 |
| 0.38768 | -0.01603 | 0.39074 | -0.02024 |
| 0.38676 | -0.01602 | 0.38952 | -0.02031 |
| 0.38553 | -0.01603 | 0.3889 | -0.02041 |
| 0.38461 | -0.01602 | 0.38768 | -0.02049 |
| 0.384 | -0.01602 | 0.38676 | -0.02057 |
| 0.38246 | -0.01602 | 0.38553 | -0.02064 |
| 0.38216 | -0.01602 | 0.38369 | -0.02078 |
| 0.38062 | -0.01601 | 0.38308 | -0.02084 |
| 0.37909 | -0.01601 | 0.38246 | -0.02091 |
| 0.37878 | -0.016 | 0.38093 | -0.02097 |
| 0.37786 | -0.016 | 0.38062 | -0.02102 |
| 0.37664 | -0.01599 | 0.3797 | -0.02107 |
| 0.37572 | -0.01599 | 0.37878 | -0.02111 |
| 0.37449 | -0.01598 | 0.37786 | -0.02114 |
| 0.37357 | -0.01597 | 0.37664 | -0.02119 |
| 0.37265 | -0.01595 | 0.37541 | -0.02122 |
| 0.37173 | -0.01594 | 0.37449 | -0.02125 |
| 0.37111 | -0.01591 | 0.37357 | -0.02127 |
| 0.36958 | -0.0159 | 0.37234 | -0.02127 |
| 0.36835 | -0.01586 | 0.37173 | -0.02129 |
| 0.36774 | -0.01584 | 0.37019 | -0.02128 |
| 0.36621 | -0.0158 | 0.36958 | -0.02127 |
| 0.36559 | -0.01577 | 0.36866 | -0.02127 |
| 0.36437 | -0.01573 | 0.36774 | -0.02125 |
| 0.36345 | -0.01569 | 0.36621 | -0.02122 |
| 0.36222 | -0.01565 | 0.36559 | -0.02118 |
| 0.3613 | -0.01561 | 0.36467 | -0.02115 |
| 0.36007 | -0.01556 | 0.36345 | -0.0211 |
| 0.35885 | -0.01553 | 0.36222 | -0.02106 |
| 0.35793 | -0.01548 | 0.3613 | -0.02098 |
| 0.35701 | -0.01545 | 0.36038 | -0.02092 |
| 0.35578 | -0.0154 | 0.35885 | -0.02084 |
| 0.35486 | -0.01537 | 0.35793 | -0.02076 |
| 0.35394 | -0.01532 | 0.35731 | -0.02068 |
| 0.35271 | -0.01528 | 0.35578 | -0.02058 |
| 0.35179 | -0.01525 | 0.35486 | -0.02048 |
| 0.35057 | -0.01522 | 0.35363 | -0.02037 |
| 0.34995 | -0.01518 | 0.35271 | -0.02027 |
| 0.34872 | -0.01515 | 0.35149 | -0.02015 |
| 0.3475 | -0.01511 | 0.34995 | -0.02003 |
| 0.34658 | -0.01508 | 0.34903 | -0.0199 |
| 0.34535 | -0.01505 | 0.34811 | -0.0198 |
| 0.34351 | -0.01498 | 0.34688 | -0.01967 |
| 0.34259 | -0.01496 | 0.34535 | -0.01954 |
| 0.34136 | -0.01493 | 0.34443 | -0.01942 |
| 0.34044 | -0.01491 | 0.3432 | -0.0193 |
| 0.33922 | -0.01488 | 0.34198 | -0.01918 |
| 0.3383 | -0.01485 | 0.34106 | -0.01905 |
| 0.33738 | -0.01483 | 0.33983 | -0.01894 |
| 0.33584 | -0.01481 | 0.33891 | -0.01881 |
| 0.33492 | -0.01478 | 0.33768 | -0.0187 |
| 0.33431 | -0.01476 | 0.33676 | -0.0186 |
| 0.33339 | -0.01474 | 0.33554 | -0.01848 |
| 0.33216 | -0.01471 | 0.33431 | -0.0184 |
| 0.33124 | -0.01469 | 0.33339 | -0.01826 |
| 0.33032 | -0.01467 | 0.33216 | -0.01817 |
| 0.32879 | -0.01465 | 0.33094 | -0.01806 |
| 0.32818 | -0.01463 | 0.33002 | -0.01798 |
| 0.32695 | -0.01461 | 0.3291 | -0.01787 |
| 0.32603 | -0.01459 | 0.32726 | -0.01779 |
| 0.32511 | -0.01457 | 0.32634 | -0.0177 |
| 0.32419 | -0.01455 | 0.32511 | -0.01761 |
| 0.32296 | -0.01454 | 0.3245 | -0.01753 |
| 0.32204 | -0.01452 | 0.32388 | -0.01743 |
| 0.32112 | -0.0145 | 0.32204 | -0.01735 |
| 0.31959 | -0.01449 | 0.32081 | -0.01727 |
| 0.31897 | -0.01447 | 0.31959 | -0.01719 |
| 0.31775 | -0.01446 | 0.31897 | -0.01711 |
| 0.31652 | -0.01444 | 0.31805 | -0.01704 |
| 0.31591 | -0.01443 | 0.31683 | -0.01696 |
| 0.31499 | -0.01441 | 0.3156 | -0.01688 |
| 0.31407 | -0.0144 | 0.31499 | -0.01683 |
| 0.31284 | -0.01438 | 0.31376 | -0.01675 |
| 0.31161 | -0.01436 | 0.31253 | -0.01669 |
| 0.31008 | -0.01435 | 0.31131 | -0.01662 |
| 0.30947 | -0.01434 | 0.31039 | -0.01654 |
| 0.30855 | -0.01432 | 0.30947 | -0.01649 |
| 0.30763 | -0.01432 | 0.30824 | -0.01641 |
| 0.30671 | -0.0143 | 0.30701 | -0.01635 |
| 0.30548 | -0.01429 | 0.3064 | -0.0163 |
| 0.30456 | -0.01427 | 0.30487 | -0.01624 |
| 0.30364 | -0.01426 | 0.30364 | -0.01618 |
| 0.30303 | -0.01424 | 0.30272 | -0.01613 |
| 0.30119 | -0.01423 | 0.30149 | -0.01607 |
| 0.30027 | -0.01422 | 0.30027 | -0.016 |
| 0.29935 | -0.01421 | 0.29935 | -0.01595 |
| 0.29904 | -0.0142 | 0.29873 | -0.01591 |
| 0.2972 | -0.01418 | 0.29751 | -0.01585 |
| 0.29658 | -0.01417 | 0.29628 | -0.01581 |
| 0.29566 | -0.01416 | 0.29505 | -0.01575 |
| 0.29474 | -0.01415 | 0.29413 | -0.0157 |
| 0.29382 | -0.01414 | 0.29321 | -0.01565 |
| 0.29229 | -0.01413 | 0.29168 | -0.01561 |
| 0.29137 | -0.01412 | 0.29076 | -0.01556 |
| 0.29045 | -0.01411 | 0.28984 | -0.01553 |
| 0.28922 | -0.0141 | 0.28892 | -0.01548 |
| 0.28861 | -0.01409 | 0.288 | -0.01544 |
| 0.28738 | -0.01408 | 0.28677 | -0.0154 |
| 0.28646 | -0.01407 | 0.28585 | -0.01535 |
| 0.28524 | -0.01406 | 0.28524 | -0.01532 |
| 0.28401 | -0.01405 | 0.28401 | -0.01528 |
| 0.2834 | -0.01404 | 0.28278 | -0.01524 |
| 0.28248 | -0.01403 | 0.28186 | -0.01522 |
| 0.28094 | -0.01403 | 0.28094 | -0.01516 |
| 0.28002 | -0.01402 | 0.27941 | -0.01515 |
| 0.27941 | -0.01402 | 0.27849 | -0.0151 |
| 0.27757 | -0.01401 | 0.27726 | -0.01507 |
| 0.27696 | -0.014 | 0.27634 | -0.01503 |
| 0.27634 | -0.01399 | 0.27542 | -0.015 |
| 0.27481 | -0.01399 | 0.2742 | -0.01499 |
| 0.27389 | -0.01398 | 0.27328 | -0.01495 |
| 0.27328 | -0.01398 | 0.27236 | -0.01491 |
| 0.27236 | -0.01397 | 0.27143 | -0.01489 |
| 0.27113 | -0.01396 | 0.27021 | -0.01485 |
| 0.27051 | -0.01396 | 0.2699 | -0.01483 |
| 0.26898 | -0.01396 | 0.26837 | -0.01482 |
| 0.26867 | -0.01395 | 0.26683 | -0.01478 |
| 0.26683 | -0.01395 | 0.26591 | -0.01475 |
| 0.26622 | -0.01394 | 0.26499 | -0.01473 |
| 0.26499 | -0.01394 | 0.26377 | -0.0147 |
| 0.26469 | -0.01393 | 0.26315 | -0.01468 |
| 0.26315 | -0.01394 | 0.26193 | -0.01465 |
| 0.26254 | -0.01393 | 0.26101 | -0.01463 |
| 0.26131 | -0.01393 | 0.26009 | -0.01461 |
| 0.26009 | -0.01392 | 0.25886 | -0.01458 |
| 0.25917 | -0.01393 | 0.25794 | -0.01456 |
| 0.25825 | -0.01392 | 0.25702 | -0.01455 |
| 0.25702 | -0.01392 | 0.2561 | -0.01453 |
| 0.2561 | -0.01392 | 0.25365 | -0.01448 |
| 0.25487 | -0.01392 | 0.25273 | -0.01448 |
| 0.25426 | -0.01392 | 0.25181 | -0.01445 |
| 0.25334 | -0.01391 | 0.25089 | -0.01442 |
| 0.25211 | -0.01391 | 0.24935 | -0.01441 |
| 0.25119 | -0.01391 | 0.24874 | -0.01439 |
| 0.24997 | -0.01391 | 0.24751 | -0.01437 |
| 0.24905 | -0.01391 | 0.24659 | -0.01436 |
| 0.24782 | -0.01391 | 0.24567 | -0.01435 |
| 0.24721 | -0.01391 | 0.24475 | -0.01433 |
| 0.24598 | -0.01391 | 0.24383 | -0.01432 |
| 0.24506 | -0.01392 | 0.2426 | -0.0143 |
| 0.24414 | -0.01391 | 0.24168 | -0.01429 |
| 0.24291 | -0.01392 | 0.24107 | -0.01427 |
| 0.24199 | -0.01392 | 0.23984 | -0.01426 |
| 0.24138 | -0.01392 | 0.23831 | -0.01426 |
| 0.24015 | -0.01392 | 0.238 | -0.01424 |
| 0.23892 | -0.01392 | 0.23647 | -0.01423 |
| 0.238 | -0.01392 | 0.23555 | -0.01421 |
| 0.23739 | -0.01392 | 0.23432 | -0.01419 |
| 0.23586 | -0.01392 | 0.2334 | -0.01418 |
| 0.23524 | -0.01393 | 0.23248 | -0.01418 |
| 0.23432 | -0.01392 | 0.23126 | -0.01416 |
| 0.2331 | -0.01393 | 0.23034 | -0.01414 |
| 0.23218 | -0.01393 | 0.22942 | -0.01413 |
| 0.23095 | -0.01394 | 0.2288 | -0.01413 |
| 0.23034 | -0.01393 | 0.22727 | -0.01413 |
| 0.22911 | -0.01394 | 0.22635 | -0.01411 |
| 0.22819 | -0.01394 | 0.22543 | -0.0141 |
| 0.22727 | -0.01395 | 0.22451 | -0.0141 |
| 0.22574 | -0.01395 | 0.22328 | -0.01408 |
| 0.22482 | -0.01395 | 0.22267 | -0.01408 |
| 0.22451 | -0.01395 | 0.22114 | -0.01407 |
| 0.22298 | -0.01395 | 0.22022 | -0.01432 |
| 0.22206 | -0.01396 | 0.21899 | -0.01426 |
| 0.22114 | -0.01396 | 0.21807 | -0.01423 |
| 0.21899 | -0.01397 | 0.21653 | -0.01422 |
| 0.21807 | -0.01397 | 0.21561 | -0.0142 |
| 0.21653 | -0.01398 | 0.21469 | -0.01419 |
| 0.21623 | -0.01398 | 0.21347 | -0.01418 |
| 0.215 | -0.01399 | 0.21285 | -0.01417 |
| 0.21377 | -0.01399 | 0.21193 | -0.01415 |
| 0.21316 | -0.01399 | 0.21071 | -0.01415 |
| 0.21224 | -0.014 | 0.20979 | -0.01414 |
| 0.21101 | -0.014 | 0.20887 | -0.01413 |
| 0.21009 | -0.014 | 0.20764 | -0.01413 |
| 0.20887 | -0.01401 | 0.20672 | -0.01412 |
| 0.20825 | -0.01401 | 0.20611 | -0.01411 |
| 0.20703 | -0.01402 | 0.20457 | -0.0141 |
| 0.20611 | -0.01402 | 0.20365 | -0.01409 |
| 0.20519 | -0.01403 | 0.20243 | -0.01409 |
| 0.20365 | -0.01403 | 0.20151 | -0.01408 |
| 0.20273 | -0.01404 | 0.20028 | -0.01407 |
| 0.20212 | -0.01405 | 0.19936 | -0.01407 |
| 0.2012 | -0.01405 | 0.19875 | -0.01406 |
| 0.19997 | -0.01405 | 0.19783 | -0.01406 |
| 0.19905 | -0.01406 | 0.1966 | -0.01405 |
| 0.19844 | -0.01406 | 0.19537 | -0.01404 |
| 0.19721 | -0.01407 | 0.19384 | -0.01404 |
| 0.19599 | -0.01407 | 0.19322 | -0.01403 |
| 0.19476 | -0.01408 | 0.19261 | -0.01403 |
| 0.19414 | -0.01409 | 0.19138 | -0.01402 |
| 0.19292 | -0.01409 | 0.19046 | -0.01402 |
| 0.192 | -0.0141 | 0.18954 | -0.01402 |
| 0.19108 | -0.0141 | 0.18832 | -0.01401 |
| 0.18985 | -0.01411 | 0.1877 | -0.01401 |
| 0.18954 | -0.01411 | 0.18648 | -0.014 |
| 0.18832 | -0.01412 | 0.18525 | -0.014 |
| 0.18709 | -0.01413 | 0.18464 | -0.014 |
| 0.18586 | -0.01413 | 0.18341 | -0.01399 |
| 0.18494 | -0.01414 | 0.18218 | -0.01399 |
| 0.18433 | -0.01415 | 0.18157 | -0.01398 |
| 0.18372 | -0.01415 | 0.18065 | -0.01398 |
| 0.18249 | -0.01415 | 0.17942 | -0.01398 |
| 0.18126 | -0.01417 | 0.1785 | -0.01398 |
| 0.18004 | -0.01417 | 0.17758 | -0.01398 |
| 0.17942 | -0.01418 | 0.17697 | -0.01397 |
| 0.1785 | -0.01419 | 0.17574 | -0.01397 |
| 0.17728 | -0.01419 | 0.17452 | -0.01397 |
| 0.17636 | -0.0142 | 0.17329 | -0.01397 |
| 0.17513 | -0.01421 | 0.17206 | -0.01396 |
| 0.17421 | -0.01421 | 0.17145 | -0.01396 |
| 0.17329 | -0.01422 | 0.17022 | -0.01396 |
| 0.17268 | -0.01423 | 0.16961 | -0.01396 |
| 0.17176 | -0.01423 | 0.16807 | -0.01396 |
| 0.17084 | -0.01424 | 0.16715 | -0.01395 |
| 0.16869 | -0.01425 | 0.16654 | -0.01395 |
| 0.16869 | -0.01425 | 0.16531 | -0.01395 |
| 0.16777 | -0.01426 | 0.1647 | -0.01395 |
| 0.16654 | -0.01427 | 0.16317 | -0.01395 |
| 0.16531 | -0.01428 | 0.16225 | -0.01395 |
| 0.16439 | -0.01428 | 0.16133 | -0.01395 |
| 0.16347 | -0.01429 | 0.16071 | -0.01395 |
| 0.16255 | -0.0143 | 0.15918 | -0.01394 |
| 0.16163 | -0.01431 | 0.15857 | -0.01394 |
| 0.16041 | -0.01431 | 0.15765 | -0.01394 |
| 0.15949 | -0.01432 | 0.15642 | -0.01394 |
| 0.15857 | -0.01433 | 0.15519 | -0.01394 |
| 0.15734 | -0.01434 | 0.15397 | -0.01394 |
| 0.15673 | -0.01434 | 0.15335 | -0.01394 |
| 0.15519 | -0.01435 | 0.15182 | -0.01394 |
| 0.15458 | -0.01436 | 0.15151 | -0.01394 |
| 0.15335 | -0.01437 | 0.14998 | -0.01394 |
| 0.15243 | -0.01437 | 0.14937 | -0.01394 |
| 0.15151 | -0.01438 | 0.14814 | -0.01394 |
| 0.15029 | -0.01439 | 0.14722 | -0.01394 |
| 0.14937 | -0.0144 | 0.1463 | -0.01394 |
| 0.14845 | -0.01441 | 0.14507 | -0.01394 |
| 0.14814 | -0.01441 | 0.14415 | -0.01394 |
| 0.1463 | -0.01442 | 0.14323 | -0.01394 |
| 0.14599 | -0.01443 | 0.14231 | -0.01394 |
| 0.14507 | -0.01444 | 0.14108 | -0.01394 |
| 0.14385 | -0.01445 | 0.14016 | -0.01394 |
| 0.14262 | -0.01446 | 0.13894 | -0.01394 |
| 0.1417 | -0.01446 | 0.13832 | -0.01394 |
| 0.14108 | -0.01447 | 0.1374 | -0.01394 |
| 0.13955 | -0.01449 | 0.13648 | -0.01395 |
| 0.13863 | -0.01449 | 0.13526 | -0.01394 |
| 0.1374 | -0.0145 | 0.13403 | -0.01395 |
| 0.1371 | -0.0145 | 0.13311 | -0.01395 |
| 0.13526 | -0.01452 | 0.13219 | -0.01395 |
| 0.13464 | -0.01453 | 0.13158 | -0.01395 |
| 0.13403 | -0.01453 | 0.13096 | -0.01395 |
| 0.1325 | -0.01454 | 0.12943 | -0.01396 |
| 0.13188 | -0.01455 | 0.12851 | -0.01395 |
| 0.13096 | -0.01456 | 0.12759 | -0.01396 |
| 0.13035 | -0.01457 | 0.12606 | -0.01396 |
| 0.12882 | -0.01458 | 0.12544 | -0.01396 |
| 0.12759 | -0.01459 | 0.12422 | -0.01396 |
| 0.12667 | -0.0146 | 0.1233 | -0.01396 |
| 0.12636 | -0.01461 | 0.12238 | -0.01396 |
| 0.12483 | -0.01462 | 0.12146 | -0.01397 |
| 0.12422 | -0.01463 | 0.12023 | -0.01397 |
| 0.12268 | -0.01464 | 0.11962 | -0.01397 |
| 0.12207 | -0.01466 | 0.11808 | -0.01398 |
| 0.12054 | -0.01466 | 0.11716 | -0.01397 |
| 0.11992 | -0.01468 | 0.11655 | -0.01397 |
| 0.1187 | -0.01468 | 0.11501 | -0.01398 |
| 0.11808 | -0.01469 | 0.11409 | -0.01398 |
| 0.11716 | -0.0147 | 0.11348 | -0.01398 |
| 0.11563 | -0.01472 | 0.11225 | -0.01398 |
| 0.11501 | -0.01473 | 0.11133 | -0.01398 |
| 0.11409 | -0.01474 | 0.11072 | -0.01399 |
| 0.11287 | -0.01476 | 0.10919 | -0.01399 |
| 0.11164 | -0.01476 | 0.10827 | -0.01399 |
| 0.11103 | -0.01478 | 0.10735 | -0.014 |
| 0.1098 | -0.01479 | 0.10643 | -0.014 |
| 0.10919 | -0.0148 | 0.10551 | -0.014 |
| 0.10827 | -0.01481 | 0.10397 | -0.014 |
| 0.10704 | -0.01482 | 0.10336 | -0.014 |
| 0.10643 | -0.01484 | 0.10183 | -0.01401 |
| 0.10489 | -0.01485 | 0.10152 | -0.014 |
| 0.10428 | -0.01486 | 0.10091 | -0.01401 |
| 0.10305 | -0.01487 | 0.09907 | -0.01401 |
| 0.10244 | -0.01489 | 0.09845 | -0.01401 |
| 0.10121 | -0.0149 | 0.09784 | -0.01401 |
| 0.10029 | -0.01491 | 0.09631 | -0.01402 |
| 0.09907 | -0.01492 | 0.09508 | -0.01402 |
| 0.09876 | -0.01494 | 0.09447 | -0.01402 |
| 0.09692 | -0.01495 | 0.09385 | -0.01402 |
| 0.09631 | -0.01496 | 0.09232 | -0.01403 |
| 0.09539 | -0.01498 | 0.09171 | -0.01403 |
| 0.09447 | -0.01499 | 0.09109 | -0.01404 |
| 0.09324 | -0.01501 | 0.08956 | -0.01403 |
| 0.09232 | -0.01502 | 0.08833 | -0.01404 |
| 0.09171 | -0.01504 | 0.08741 | -0.01404 |
| 0.09048 | -0.01505 | 0.08649 | -0.01405 |
| 0.08894 | -0.01506 | 0.08557 | -0.01405 |
| 0.08833 | -0.01508 | 0.08434 | -0.01405 |
| 0.08741 | -0.01509 | 0.08342 | -0.01405 |
| 0.08649 | -0.01511 | 0.0825 | -0.01406 |
| 0.08557 | -0.01512 | 0.0822 | -0.01406 |
| 0.08434 | -0.01513 | 0.08005 | -0.01406 |
| 0.08404 | -0.01515 | 0.07974 | -0.01406 |
| 0.0825 | -0.01517 | 0.07882 | -0.01407 |
| 0.08189 | -0.01518 | 0.0776 | -0.01407 |
| 0.08097 | -0.0152 | 0.07668 | -0.01407 |
| 0.08005 | -0.01522 | 0.07545 | -0.01407 |
| 0.07913 | -0.01523 | 0.07422 | -0.01408 |
| 0.07729 | -0.01525 | 0.073 | -0.01408 |
| 0.07668 | -0.01527 | 0.07269 | -0.01408 |
| 0.07576 | -0.01528 | 0.07177 | -0.01409 |
| 0.07453 | -0.0153 | 0.07054 | -0.01409 |
| 0.07392 | -0.01531 | 0.0687 | -0.01409 |
| 0.073 | -0.01533 | 0.0684 | -0.0141 |
| 0.07177 | -0.01534 | 0.06748 | -0.0141 |
| 0.07116 | -0.01536 | 0.06625 | -0.0141 |
| 0.06993 | -0.01538 | 0.06533 | -0.01411 |
| 0.06901 | -0.0154 | 0.06471 | -0.01411 |
| 0.06778 | -0.01542 | 0.06349 | -0.01411 |
| 0.06717 | -0.01544 | 0.06287 | -0.01412 |
| 0.06594 | -0.01545 | 0.06165 | -0.01412 |
| 0.06471 | -0.01547 | 0.06073 | -0.01413 |
| 0.06379 | -0.01548 | 0.05919 | -0.01413 |
| 0.06349 | -0.01551 | 0.05889 | -0.01413 |
| 0.06195 | -0.01552 | 0.05735 | -0.01413 |
| 0.06103 | -0.01554 | 0.05705 | -0.01414 |
| 0.05981 | -0.01556 | 0.05551 | -0.01414 |
| 0.05919 | -0.01558 | 0.05459 | -0.01415 |
| 0.05827 | -0.0156 | 0.05367 | -0.01415 |
| 0.05674 | -0.01562 | 0.05245 | -0.01416 |
| 0.05551 | -0.01563 | 0.05183 | -0.01416 |
| 0.0549 | -0.01565 | 0.05061 | -0.01416 |
| 0.05459 | -0.01567 | 0.04969 | -0.01417 |
| 0.05337 | -0.01569 | 0.04846 | -0.01417 |
| 0.05245 | -0.01571 | 0.04785 | -0.01417 |
| 0.05122 | -0.01573 | 0.04631 | -0.01418 |
| 0.04999 | -0.01575 | 0.04539 | -0.01418 |
| 0.04938 | -0.01577 | 0.04447 | -0.01419 |
| 0.04846 | -0.01579 | 0.04417 | -0.0142 |
| 0.04723 | -0.01582 | 0.04233 | -0.0142 |
| 0.04631 | -0.01583 | 0.04171 | -0.0142 |
| 0.04539 | -0.01585 | 0.04049 | -0.01421 |
| 0.04447 | -0.01588 | 0.03987 | -0.01422 |
| 0.04355 | -0.0159 | 0.03834 | -0.01422 |
| 0.04263 | -0.01592 | 0.03772 | -0.01423 |
| 0.04141 | -0.01594 | 0.03711 | -0.01424 |
| 0.04049 | -0.01596 | 0.03558 | -0.01424 |
| 0.03987 | -0.01598 | 0.03466 | -0.01425 |
| 0.03864 | -0.016 | 0.03312 | -0.01425 |
| 0.03742 | -0.01602 | 0.03251 | -0.01426 |
| 0.0365 | -0.01604 | 0.03159 | -0.01427 |
| 0.0365 | -0.01607 | 0.03067 | -0.01428 |
| 0.03435 | -0.01609 | 0.02975 | -0.01428 |
| 0.03374 | -0.01611 | 0.02883 | -0.01429 |
| 0.03312 | -0.01613 | 0.0276 | -0.01429 |
| 0.03159 | -0.01615 | 0.02699 | -0.0143 |
| 0.03067 | -0.01618 | 0.02546 | -0.01431 |
| 0.02975 | -0.0162 | 0.02484 | -0.01432 |
| 0.02852 | -0.01623 | 0.02362 | -0.01433 |
| 0.02791 | -0.01625 | 0.023 | -0.01433 |
| 0.02699 | -0.01627 | 0.02208 | -0.01434 |
| 0.02576 | -0.01629 | 0.02055 | -0.01435 |
| 0.02515 | -0.01632 | 0.01994 | -0.01436 |
| 0.02392 | -0.01634 | 0.0184 | -0.01436 |
| 0.02331 | -0.01637 | 0.01748 | -0.01437 |
| 0.02239 | -0.01639 | 0.01687 | -0.01438 |
| 0.02116 | -0.01642 | 0.01595 | -0.01418 |
| 0.02116 | -0.01599 | 0.01656 | -0.01372 |
| 0.02147 | -0.01559 | 0.01748 | -0.0133 |
| 0.02239 | -0.01522 | 0.01779 | -0.01291 |
| 0.02331 | -0.01486 | 0.01871 | -0.01254 |
| 0.02331 | -0.01452 | 0.01932 | -0.01219 |
| 0.02362 | -0.01419 | 0.01963 | -0.01185 |
| 0.02454 | -0.01387 | 0.02055 | -0.01152 |
| 0.02515 | -0.01356 | 0.02116 | -0.01119 |
| 0.02638 | -0.01325 | 0.02178 | -0.01088 |
| 0.02668 | -0.01295 | 0.02239 | -0.01056 |
| 0.02699 | -0.01265 | 0.023 | -0.01026 |
| 0.02791 | -0.01237 | 0.02362 | -0.00996 |
| 0.02822 | -0.01208 | 0.02423 | -0.00967 |
| 0.02914 | -0.0118 | 0.02484 | -0.00939 |
| 0.03036 | -0.01153 | 0.02576 | -0.0091 |
| 0.03006 | -0.01126 | 0.02638 | -0.00882 |
| 0.03098 | -0.01099 | 0.0273 | -0.00856 |
| 0.03159 | -0.01073 | 0.0276 | -0.00829 |
| 0.0322 | -0.01047 | 0.02883 | -0.00802 |
| 0.03282 | -0.01022 | 0.02914 | -0.00777 |
| 0.03404 | -0.00996 | 0.02975 | -0.00751 |
| 0.03466 | -0.00972 | 0.03067 | -0.00726 |
| 0.03558 | -0.00947 | 0.03098 | -0.00701 |
| 0.03588 | -0.00923 | 0.03159 | -0.00677 |
| 0.0365 | -0.00899 | 0.03312 | -0.00653 |
| 0.03742 | -0.00875 | 0.03374 | -0.0063 |
| 0.03834 | -0.00852 | 0.03466 | -0.00606 |
| 0.03864 | -0.00828 | 0.03527 | -0.00584 |
| 0.03956 | -0.00806 | 0.03588 | -0.00561 |
| 0.03987 | -0.00783 | 0.0368 | -0.0054 |
| 0.04079 | -0.00761 | 0.03711 | -0.00518 |
| 0.04202 | -0.00739 | 0.03803 | -0.00496 |
| 0.04202 | -0.00717 | 0.03926 | -0.00475 |
| 0.04294 | -0.00695 | 0.03987 | -0.00454 |
| 0.04325 | -0.00674 | 0.04049 | -0.00433 |
| 0.04417 | -0.00653 | 0.04141 | -0.00413 |
| 0.04478 | -0.00632 | 0.04233 | -0.00393 |
| 0.04601 | -0.00611 | 0.04294 | -0.00373 |
| 0.04631 | -0.00592 | 0.04386 | -0.00354 |
| 0.04723 | -0.00571 | 0.04447 | -0.00334 |
| 0.04815 | -0.00551 | 0.04539 | -0.00316 |
| 0.04877 | -0.0053 | 0.04601 | -0.00297 |
| 0.04938 | -0.00511 | 0.04693 | -0.00279 |
| 0.0503 | -0.00492 | 0.04785 | -0.00261 |
| 0.05122 | -0.00472 | 0.04877 | -0.00243 |
| 0.05183 | -0.00453 | 0.04999 | -0.00208 |
| 0.05306 | -0.00435 | 0.05122 | -0.0019 |
| 0.05337 | -0.00416 | 0.05153 | -0.00174 |
| 0.05398 | -0.00398 | 0.05275 | -0.00157 |
| 0.0549 | -0.00379 | 0.05337 | -0.0014 |
| 0.05551 | -0.00361 | 0.05429 | -0.00124 |
| 0.05674 | -0.00343 | 0.05521 | -0.00107 |
| 0.05766 | -0.00325 | 0.05582 | -9.19E-04 |
| 0.05766 | -0.00307 | 0.05643 | -7.58E-04 |
| 0.05858 | -0.0029 | 0.05766 | -6.03E-04 |
| 0.0595 | -0.00273 | 0.05827 | -4.54E-04 |
| 0.06042 | -0.00256 | 0.05919 | -3.03E-04 |
| 0.06134 | -0.00239 | 0.06011 | -1.58E-04 |
| 0.06226 | -0.00222 | 0.06073 | -1.18E-05 |
| 0.06318 | -0.00205 | 0.06165 | 2.01E-05 |
| 0.06318 | -0.00189 | 0.06441 | 5.63E-04 |
| 0.06441 | -0.00172 | 0.06502 | 6.96E-04 |
| 0.06533 | -0.00156 | 0.06594 | 8.36E-04 |
| 0.06656 | -0.0014 | 0.06686 | 9.67E-04 |
| 0.06686 | -0.00124 | 0.06778 | 0.0011 |
| 0.06778 | -0.00108 | 0.0684 | 0.00123 |
| 0.0684 | -9.30E-04 | 0.06993 | 0.00136 |
| 0.06962 | -7.72E-04 | 0.06993 | 0.00148 |
| 0.07024 | -6.17E-04 | 0.07054 | 0.00161 |
| 0.07054 | -4.65E-04 | 0.07208 | 0.00173 |
| 0.07177 | -3.09E-04 | 0.07238 | 0.00185 |
| 0.07269 | -1.64E-04 | 0.07392 | 0.00198 |
| 0.073 | -1.86E-05 | 0.07453 | 0.0021 |
| 0.07422 | 1.27E-04 | 0.07576 | 0.00222 |
| 0.07514 | 2.01E-04 | 0.07637 | 0.00233 |
| 0.07576 | 4.20E-04 | 0.07698 | 0.00245 |
| 0.07668 | 5.57E-04 | 0.0779 | 0.00257 |
| 0.07698 | 7.06E-04 | 0.07882 | 0.00267 |
| 0.0779 | 8.40E-04 | 0.07944 | 0.00279 |
| 0.07913 | 9.86E-04 | 0.08066 | 0.0029 |
| 0.07974 | 0.00112 | 0.08158 | 0.003 |
| 0.08158 | 0.00139 | 0.08281 | 0.00311 |
| 0.0822 | 0.00153 | 0.08312 | 0.00321 |
| 0.08281 | 0.00166 | 0.08434 | 0.00332 |
| 0.08434 | 0.00179 | 0.08465 | 0.00342 |
| 0.08526 | 0.00192 | 0.08588 | 0.00352 |
| 0.08557 | 0.00205 | 0.0871 | 0.00363 |
| 0.0868 | 0.00218 | 0.08772 | 0.00372 |
| 0.0871 | 0.00232 | 0.08864 | 0.00382 |
| 0.08833 | 0.00244 | 0.08925 | 0.00392 |
| 0.08864 | 0.00256 | 0.09048 | 0.00401 |
| 0.08986 | 0.00269 | 0.0914 | 0.0041 |
| 0.09109 | 0.00281 | 0.09171 | 0.0042 |
| 0.09171 | 0.00293 | 0.09324 | 0.00429 |
| 0.09232 | 0.00306 | 0.09385 | 0.00439 |
| 0.09355 | 0.00317 | 0.09477 | 0.00447 |
| 0.09416 | 0.0033 | 0.09569 | 0.00456 |
| 0.09508 | 0.00342 | 0.09692 | 0.00464 |
| 0.096 | 0.00353 | 0.09784 | 0.00473 |
| 0.09661 | 0.00364 | 0.09845 | 0.00481 |
| 0.09784 | 0.00376 | 0.09937 | 0.00489 |
| 0.09845 | 0.00387 | 0.1006 | 0.00498 |
| 0.09968 | 0.00399 | 0.10091 | 0.00506 |
| 0.09999 | 0.0041 | 0.10183 | 0.00514 |
| 0.10091 | 0.00421 | 0.10336 | 0.00522 |
| 0.10213 | 0.00432 | 0.10367 | 0.0053 |
| 0.10336 | 0.00443 | 0.10489 | 0.00538 |
| 0.10367 | 0.00453 | 0.10581 | 0.00545 |
| 0.10459 | 0.00464 | 0.10673 | 0.00553 |
| 0.1052 | 0.00475 | 0.10765 | 0.0056 |
| 0.10673 | 0.00486 | 0.10857 | 0.00568 |
| 0.10704 | 0.00496 | 0.10949 | 0.00575 |
| 0.10765 | 0.00506 | 0.11072 | 0.00582 |
| 0.10919 | 0.00516 | 0.11133 | 0.00589 |
| 0.10949 | 0.00526 | 0.11287 | 0.00596 |
| 0.11103 | 0.00536 | 0.11317 | 0.00603 |
| 0.11164 | 0.00547 | 0.1144 | 0.0061 |
| 0.11195 | 0.00556 | 0.11532 | 0.00616 |
| 0.11379 | 0.00566 | 0.11563 | 0.00623 |
| 0.11409 | 0.00576 | 0.11685 | 0.0063 |
| 0.11532 | 0.00586 | 0.11747 | 0.00636 |
| 0.11593 | 0.00594 | 0.119 | 0.00642 |
| 0.11685 | 0.00604 | 0.11962 | 0.00648 |
| 0.11747 | 0.00613 | 0.12023 | 0.00655 |
| 0.1187 | 0.00623 | 0.12146 | 0.00661 |
| 0.11962 | 0.00632 | 0.12299 | 0.00667 |
| 0.12054 | 0.00641 | 0.1233 | 0.00673 |
| 0.12084 | 0.00649 | 0.12422 | 0.00678 |
| 0.12207 | 0.00659 | 0.12514 | 0.00684 |
| 0.12299 | 0.00667 | 0.12575 | 0.0069 |
| 0.12422 | 0.00676 | 0.1279 | 0.00696 |
| 0.12483 | 0.00685 | 0.1282 | 0.00701 |
| 0.12575 | 0.00693 | 0.12882 | 0.00707 |
| 0.12667 | 0.00702 | 0.12974 | 0.00713 |
| 0.12759 | 0.00711 | 0.13096 | 0.00717 |
| 0.1282 | 0.00719 | 0.13188 | 0.00722 |
| 0.12974 | 0.00727 | 0.13311 | 0.00728 |
| 0.13004 | 0.00735 | 0.13342 | 0.00733 |
| 0.13096 | 0.00743 | 0.13464 | 0.00738 |
| 0.13188 | 0.00751 | 0.13587 | 0.00743 |
| 0.1328 | 0.00759 | 0.13648 | 0.00748 |
| 0.13403 | 0.00768 | 0.1374 | 0.00753 |
| 0.13434 | 0.00775 | 0.13832 | 0.00758 |
| 0.13556 | 0.00782 | 0.13955 | 0.00762 |
| 0.13648 | 0.00791 | 0.14016 | 0.00767 |
| 0.1374 | 0.00798 | 0.14108 | 0.00772 |
| 0.13832 | 0.00805 | 0.14231 | 0.00776 |
| 0.13924 | 0.00812 | 0.14293 | 0.0078 |
| 0.14047 | 0.0082 | 0.14415 | 0.00785 |
| 0.14078 | 0.00827 | 0.14507 | 0.00789 |
| 0.142 | 0.00834 | 0.14599 | 0.00794 |
| 0.14262 | 0.00841 | 0.14691 | 0.00798 |
| 0.14354 | 0.00849 | 0.14814 | 0.00802 |
| 0.14477 | 0.00855 | 0.14937 | 0.00806 |
| 0.14569 | 0.00862 | 0.14998 | 0.0081 |
| 0.14661 | 0.00869 | 0.1509 | 0.00814 |
| 0.14722 | 0.00876 | 0.15182 | 0.00819 |
| 0.14814 | 0.00883 | 0.15274 | 0.00822 |
| 0.14937 | 0.00889 | 0.15397 | 0.00826 |
| 0.14998 | 0.00896 | 0.15489 | 0.0083 |
| 0.1509 | 0.00902 | 0.1555 | 0.00833 |
| 0.15182 | 0.00909 | 0.15734 | 0.00838 |
| 0.15274 | 0.00916 | 0.15765 | 0.00841 |
| 0.15397 | 0.00921 | 0.15857 | 0.00844 |
| 0.15519 | 0.00928 | 0.15979 | 0.00848 |
| 0.1555 | 0.00934 | 0.16133 | 0.00855 |
| 0.15673 | 0.0094 | 0.16255 | 0.00858 |
| 0.15703 | 0.00946 | 0.16347 | 0.00861 |
| 0.15887 | 0.00952 | 0.16409 | 0.00865 |
| 0.15918 | 0.00958 | 0.16531 | 0.00868 |
| 0.16041 | 0.00963 | 0.16593 | 0.00871 |
| 0.16163 | 0.00969 | 0.16715 | 0.00875 |
| 0.16194 | 0.00976 | 0.16838 | 0.00878 |
| 0.16317 | 0.00981 | 0.16869 | 0.00881 |
| 0.16378 | 0.00987 | 0.17022 | 0.00884 |
| 0.1647 | 0.00992 | 0.17114 | 0.00887 |
| 0.16562 | 0.00997 | 0.17206 | 0.0089 |
| 0.16685 | 0.01003 | 0.17268 | 0.00893 |
| 0.16777 | 0.01009 | 0.1736 | 0.00896 |
| 0.16869 | 0.01014 | 0.17452 | 0.00898 |
| 0.16992 | 0.01019 | 0.17605 | 0.00901 |
| 0.17022 | 0.01024 | 0.17697 | 0.00904 |
| 0.17176 | 0.0103 | 0.17789 | 0.00907 |
| 0.17237 | 0.01034 | 0.17881 | 0.00909 |
| 0.1736 | 0.01039 | 0.18004 | 0.00912 |
| 0.17421 | 0.01044 | 0.18065 | 0.00915 |
| 0.17482 | 0.01049 | 0.18218 | 0.00917 |
| 0.17605 | 0.01054 | 0.18249 | 0.0092 |
| 0.17697 | 0.01059 | 0.18341 | 0.00923 |
| 0.17789 | 0.01064 | 0.18464 | 0.00925 |
| 0.17912 | 0.01068 | 0.18586 | 0.00928 |
| 0.18004 | 0.01073 | 0.18648 | 0.00929 |
| 0.18065 | 0.01078 | 0.18801 | 0.00932 |
| 0.18157 | 0.01082 | 0.18832 | 0.00934 |
| 0.1828 | 0.01087 | 0.18924 | 0.00937 |
| 0.18372 | 0.01091 | 0.19046 | 0.00939 |
| 0.18464 | 0.01096 | 0.19169 | 0.00941 |
| 0.18586 | 0.01099 | 0.19292 | 0.00943 |
| 0.18648 | 0.01104 | 0.19384 | 0.00945 |
| 0.18709 | 0.01108 | 0.19414 | 0.00948 |
| 0.18832 | 0.01112 | 0.19537 | 0.0095 |
| 0.18954 | 0.01116 | 0.19629 | 0.00952 |
| 0.19046 | 0.01121 | 0.19752 | 0.00954 |
| 0.1923 | 0.01128 | 0.19813 | 0.00956 |
| 0.19261 | 0.01132 | 0.19905 | 0.00958 |
| 0.19414 | 0.01136 | 0.20028 | 0.0096 |
| 0.19445 | 0.01139 | 0.20151 | 0.00962 |
| 0.19629 | 0.01144 | 0.20243 | 0.00964 |
| 0.19691 | 0.01147 | 0.20335 | 0.00966 |
| 0.19783 | 0.01151 | 0.20427 | 0.00968 |
| 0.19875 | 0.01155 | 0.20519 | 0.0097 |
| 0.19967 | 0.01158 | 0.20611 | 0.00972 |
| 0.20089 | 0.01162 | 0.20733 | 0.00974 |
| 0.20212 | 0.01166 | 0.20825 | 0.00975 |
| 0.20273 | 0.01169 | 0.20887 | 0.00977 |
| 0.20365 | 0.01172 | 0.2104 | 0.00978 |
| 0.20457 | 0.01176 | 0.21101 | 0.00981 |
| 0.20519 | 0.01179 | 0.21224 | 0.00982 |
| 0.20641 | 0.01182 | 0.21285 | 0.00984 |
| 0.20733 | 0.01186 | 0.21408 | 0.00986 |
| 0.20856 | 0.01189 | 0.21531 | 0.00987 |
| 0.21009 | 0.01192 | 0.21561 | 0.00989 |
| 0.2104 | 0.01195 | 0.21684 | 0.0099 |
| 0.21132 | 0.01198 | 0.21837 | 0.00992 |
| 0.21224 | 0.01201 | 0.21899 | 0.00994 |
| 0.21347 | 0.01205 | 0.21991 | 0.00995 |
| 0.21408 | 0.01207 | 0.22114 | 0.00997 |
| 0.215 | 0.01211 | 0.22175 | 0.00998 |
| 0.21623 | 0.01213 | 0.22298 | 0.01 |
| 0.21715 | 0.01217 | 0.2239 | 0.01001 |
| 0.21776 | 0.01219 | 0.22482 | 0.01003 |
| 0.21899 | 0.01222 | 0.22574 | 0.01004 |
| 0.22022 | 0.01224 | 0.22696 | 0.01006 |
| 0.22083 | 0.01227 | 0.22788 | 0.01007 |
| 0.22206 | 0.0123 | 0.2288 | 0.01008 |
| 0.22267 | 0.01233 | 0.23003 | 0.0101 |
| 0.22359 | 0.01235 | 0.23064 | 0.01011 |
| 0.22482 | 0.01238 | 0.23156 | 0.01012 |
| 0.22604 | 0.0124 | 0.23279 | 0.01014 |
| 0.22696 | 0.01243 | 0.23371 | 0.01015 |
| 0.22788 | 0.01246 | 0.23463 | 0.01017 |
| 0.2285 | 0.01248 | 0.23586 | 0.01018 |
| 0.22972 | 0.0125 | 0.23678 | 0.01019 |
| 0.23064 | 0.01253 | 0.2377 | 0.0102 |
| 0.23187 | 0.01255 | 0.23892 | 0.01022 |
| 0.23279 | 0.01258 | 0.23923 | 0.01023 |
| 0.23371 | 0.0126 | 0.24046 | 0.01024 |
| 0.23494 | 0.01262 | 0.24199 | 0.01025 |
| 0.23555 | 0.01264 | 0.2426 | 0.01027 |
| 0.23708 | 0.01266 | 0.24322 | 0.01027 |
| 0.23708 | 0.01269 | 0.24475 | 0.01029 |
| 0.23831 | 0.01271 | 0.24567 | 0.0103 |
| 0.23923 | 0.01273 | 0.2469 | 0.01031 |
| 0.24046 | 0.01274 | 0.24782 | 0.01033 |
| 0.24138 | 0.01277 | 0.24843 | 0.01034 |
| 0.24291 | 0.01279 | 0.24966 | 0.01034 |
| 0.24352 | 0.01281 | 0.25058 | 0.01036 |
| 0.24444 | 0.01283 | 0.2515 | 0.01037 |
| 0.24536 | 0.01285 | 0.25211 | 0.01038 |
| 0.24629 | 0.01287 | 0.25365 | 0.01039 |
| 0.24751 | 0.01289 | 0.25457 | 0.0104 |
| 0.24843 | 0.01291 | 0.25549 | 0.01041 |
| 0.24905 | 0.01292 | 0.25641 | 0.01043 |
| 0.25089 | 0.01294 | 0.25733 | 0.01043 |
| 0.25089 | 0.01296 | 0.25886 | 0.01044 |
| 0.25242 | 0.01298 | 0.25947 | 0.01045 |
| 0.25303 | 0.013 | 0.26009 | 0.01046 |
| 0.25395 | 0.01301 | 0.26131 | 0.01047 |
| 0.25518 | 0.01304 | 0.26193 | 0.01048 |
| 0.25579 | 0.01305 | 0.26377 | 0.01049 |
| 0.25733 | 0.01306 | 0.26438 | 0.01051 |
| 0.25794 | 0.01309 | 0.26561 | 0.01051 |
| 0.25886 | 0.0131 | 0.26653 | 0.01053 |
| 0.25978 | 0.01312 | 0.26714 | 0.01054 |
| 0.26101 | 0.01314 | 0.26837 | 0.01055 |
| 0.26223 | 0.01315 | 0.26959 | 0.01056 |
| 0.26315 | 0.01316 | 0.27051 | 0.01056 |
| 0.26407 | 0.01318 | 0.27113 | 0.01058 |
| 0.26469 | 0.01319 | 0.27266 | 0.01059 |
| 0.26591 | 0.01321 | 0.27297 | 0.0106 |
| 0.26745 | 0.01322 | 0.2745 | 0.0106 |
| 0.26806 | 0.01324 | 0.27481 | 0.01061 |
| 0.26867 | 0.01326 | 0.27634 | 0.01062 |
| 0.27021 | 0.01327 | 0.27726 | 0.01064 |
| 0.27082 | 0.01328 | 0.27788 | 0.01064 |
| 0.27205 | 0.01329 | 0.27972 | 0.01065 |
| 0.27266 | 0.01331 | 0.28033 | 0.01066 |
| 0.27358 | 0.01333 | 0.28125 | 0.01067 |
| 0.2745 | 0.01333 | 0.28217 | 0.01069 |
| 0.27542 | 0.01335 | 0.28309 | 0.01069 |
| 0.27665 | 0.01337 | 0.28401 | 0.0107 |
| 0.27788 | 0.01338 | 0.28493 | 0.01071 |
| 0.2791 | 0.01339 | 0.28616 | 0.01072 |
| 0.27941 | 0.0134 | 0.28708 | 0.01073 |
| 0.28033 | 0.01341 | 0.2883 | 0.01074 |
| 0.28156 | 0.01343 | 0.28922 | 0.01075 |
| 0.28278 | 0.01343 | 0.28984 | 0.01075 |
| 0.2834 | 0.01345 | 0.29106 | 0.01077 |
| 0.28462 | 0.01346 | 0.29198 | 0.01077 |
| 0.28585 | 0.01348 | 0.2926 | 0.01079 |
| 0.28646 | 0.01349 | 0.29382 | 0.01079 |
| 0.28738 | 0.0135 | 0.29474 | 0.0108 |
| 0.28922 | 0.01351 | 0.29566 | 0.01082 |
| 0.28953 | 0.01352 | 0.29751 | 0.01082 |
| 0.29045 | 0.01353 | 0.29781 | 0.01083 |
| 0.29168 | 0.01354 | 0.29904 | 0.01084 |
| 0.29229 | 0.01355 | 0.29996 | 0.01085 |
| 0.29321 | 0.01357 | 0.30088 | 0.01086 |
| 0.29474 | 0.01358 | 0.3018 | 0.01086 |
| 0.29536 | 0.01359 | 0.30303 | 0.01088 |
| 0.29658 | 0.0136 | 0.30395 | 0.01088 |
| 0.2972 | 0.01361 | 0.30456 | 0.01089 |
| 0.29843 | 0.01361 | 0.30609 | 0.0109 |
| 0.29935 | 0.01363 | 0.30701 | 0.01091 |
| 0.30027 | 0.01364 | 0.30855 | 0.01092 |
| 0.30119 | 0.01365 | 0.30885 | 0.01093 |
| 0.30241 | 0.01366 | 0.30977 | 0.01094 |
| 0.30333 | 0.01367 | 0.31069 | 0.01095 |
| 0.30456 | 0.01368 | 0.31161 | 0.01096 |
| 0.30548 | 0.01369 | 0.31192 | 0.01097 |
| 0.3064 | 0.0137 | 0.31376 | 0.01098 |
| 0.30763 | 0.01371 | 0.31499 | 0.01098 |
| 0.30824 | 0.01372 | 0.3156 | 0.01099 |
| 0.30916 | 0.01373 | 0.31683 | 0.011 |
| 0.31039 | 0.01374 | 0.31805 | 0.01101 |
| 0.31131 | 0.01375 | 0.31928 | 0.01102 |
| 0.31223 | 0.01376 | 0.31989 | 0.01103 |
| 0.31315 | 0.01377 | 0.32051 | 0.01104 |
| 0.31437 | 0.01378 | 0.32173 | 0.01105 |
| 0.3156 | 0.01379 | 0.32235 | 0.01106 |
| 0.31621 | 0.0138 | 0.32388 | 0.01107 |
| 0.31713 | 0.01381 | 0.3248 | 0.01108 |
| 0.31775 | 0.01381 | 0.32542 | 0.01109 |
| 0.31897 | 0.01383 | 0.32695 | 0.01109 |
| 0.32051 | 0.01383 | 0.32787 | 0.0111 |
| 0.32112 | 0.01384 | 0.32879 | 0.01112 |
| 0.32265 | 0.01386 | 0.33002 | 0.01112 |
| 0.32358 | 0.01387 | 0.33063 | 0.01113 |
| 0.32419 | 0.01387 | 0.33186 | 0.01114 |
| 0.32511 | 0.01389 | 0.33278 | 0.01115 |
| 0.32634 | 0.01389 | 0.33339 | 0.01116 |
| 0.32695 | 0.0139 | 0.33523 | 0.01117 |
| 0.32818 | 0.01391 | 0.33523 | 0.01118 |
| 0.3294 | 0.01392 | 0.33707 | 0.01118 |
| 0.33002 | 0.01393 | 0.3383 | 0.0112 |
| 0.33094 | 0.01394 | 0.3386 | 0.01121 |
| 0.33278 | 0.01395 | 0.33983 | 0.01122 |
| 0.33308 | 0.01396 | 0.34075 | 0.01123 |
| 0.334 | 0.01396 | 0.34136 | 0.01123 |
| 0.33462 | 0.01398 | 0.3429 | 0.01124 |
| 0.33584 | 0.01398 | 0.34351 | 0.01126 |
| 0.33646 | 0.01399 | 0.34474 | 0.01126 |
| 0.33799 | 0.01401 | 0.34535 | 0.01127 |
| 0.33922 | 0.01401 | 0.34627 | 0.01128 |
| 0.33983 | 0.01402 | 0.3475 | 0.01129 |
| 0.34075 | 0.01403 | 0.34872 | 0.0113 |
| 0.34167 | 0.01404 | 0.34995 | 0.01132 |
| 0.3429 | 0.01405 | 0.35057 | 0.01132 |
| 0.34382 | 0.01406 | 0.35149 | 0.01133 |
| 0.34474 | 0.01407 | 0.35271 | 0.01134 |
| 0.34596 | 0.01407 | 0.35302 | 0.01135 |
| 0.34719 | 0.01409 | 0.35486 | 0.01136 |
| 0.34811 | 0.0141 | 0.35578 | 0.01137 |
| 0.34872 | 0.01411 | 0.3567 | 0.01138 |
| 0.35026 | 0.01412 | 0.35731 | 0.0114 |
| 0.35026 | 0.01412 | 0.35823 | 0.01141 |
| 0.3521 | 0.01414 | 0.35946 | 0.01141 |
| 0.35271 | 0.01415 | 0.36069 | 0.01142 |
| 0.35363 | 0.01415 | 0.36161 | 0.01143 |
| 0.35455 | 0.01417 | 0.36253 | 0.01145 |
| 0.35547 | 0.01417 | 0.36375 | 0.01145 |
| 0.35701 | 0.01418 | 0.36467 | 0.01146 |
| 0.35762 | 0.01419 | 0.36529 | 0.01148 |
| 0.35854 | 0.0142 | 0.36651 | 0.01148 |
| 0.35977 | 0.01421 | 0.36713 | 0.01149 |
| 0.36099 | 0.01423 | 0.36866 | 0.01151 |
| 0.36161 | 0.01423 | 0.36927 | 0.01151 |
| 0.36253 | 0.01425 | 0.3705 | 0.01152 |
| 0.36345 | 0.01425 | 0.37111 | 0.01154 |
| 0.36498 | 0.01427 | 0.37234 | 0.01155 |
| 0.36529 | 0.01428 | 0.37357 | 0.01156 |
| 0.36651 | 0.01429 | 0.37418 | 0.01157 |
| 0.36743 | 0.0143 | 0.3748 | 0.01158 |
| 0.36866 | 0.01431 | 0.37602 | 0.01159 |
| 0.36989 | 0.01432 | 0.37756 | 0.0116 |
| 0.3705 | 0.01433 | 0.37817 | 0.01161 |
| 0.37142 | 0.01434 | 0.3797 | 0.01162 |
| 0.37265 | 0.01436 | 0.38001 | 0.01163 |
| 0.37357 | 0.01436 | 0.38093 | 0.01165 |
| 0.37449 | 0.01438 | 0.38185 | 0.01165 |
| 0.37572 | 0.01439 | 0.38308 | 0.01167 |
| 0.37572 | 0.0144 | 0.3843 | 0.01168 |
| 0.37725 | 0.01441 | 0.38492 | 0.01169 |
| 0.37848 | 0.01443 | 0.38584 | 0.0117 |
| 0.3797 | 0.01443 | 0.38706 | 0.01172 |
| 0.38062 | 0.01444 | 0.38829 | 0.01173 |
| 0.38185 | 0.01446 | 0.3886 | 0.01174 |
| 0.38216 | 0.01447 | 0.39013 | 0.01174 |
| 0.38308 | 0.01448 | 0.39074 | 0.01176 |
| 0.38461 | 0.0145 | 0.39197 | 0.01177 |
| 0.38553 | 0.01451 | 0.39289 | 0.01179 |
| 0.38645 | 0.01452 | 0.39412 | 0.01179 |
| 0.38737 | 0.01453 | 0.39504 | 0.01181 |
| 0.38798 | 0.01455 | 0.39626 | 0.01182 |
| 0.3889 | 0.01456 | 0.39718 | 0.01183 |
| 0.39013 | 0.01457 | 0.3981 | 0.01185 |
| 0.39166 | 0.01459 | 0.39933 | 0.01186 |
| 0.39228 | 0.01461 | 0.39994 | 0.01188 |
| 0.3932 | 0.01461 | 0.40087 | 0.01188 |
| 0.39412 | 0.01463 | 0.40179 | 0.01189 |
| 0.39534 | 0.01464 | 0.40301 | 0.01191 |
| 0.39626 | 0.01466 | 0.40363 | 0.01192 |
| 0.39718 | 0.01467 | 0.40485 | 0.01194 |
| 0.3981 | 0.01468 | 0.40577 | 0.01195 |
| 0.39933 | 0.0147 | 0.40669 | 0.01196 |
| 0.39994 | 0.01472 | 0.40761 | 0.01197 |
| 0.40087 | 0.01473 | 0.40915 | 0.01199 |
| 0.4024 | 0.01475 | 0.41007 | 0.012 |
| 0.40332 | 0.01476 | 0.41037 | 0.01201 |
| 0.40424 | 0.01478 | 0.4116 | 0.01202 |
| 0.40485 | 0.01479 | 0.41252 | 0.01204 |
| 0.40577 | 0.01481 | 0.41405 | 0.01205 |
| 0.407 | 0.01482 | 0.41497 | 0.01207 |
| 0.40792 | 0.01484 | 0.41559 | 0.01208 |
| 0.40853 | 0.01486 | 0.41589 | 0.0121 |
| 0.41007 | 0.01487 | 0.41773 | 0.01212 |
| 0.41099 | 0.01489 | 0.41865 | 0.01214 |
| 0.41129 | 0.01491 | 0.41957 | 0.01215 |
| 0.41313 | 0.01492 | 0.4208 | 0.01217 |
| 0.41405 | 0.01494 | 0.42172 | 0.01218 |
| 0.41497 | 0.01495 | 0.42233 | 0.0122 |
| 0.41559 | 0.01497 | 0.42356 | 0.01221 |
| 0.41681 | 0.01499 | 0.42448 | 0.01223 |
| 0.41804 | 0.01501 | 0.4254 | 0.01225 |
| 0.41865 | 0.01503 | 0.42632 | 0.01226 |
| 0.41957 | 0.01504 | 0.42755 | 0.01228 |
| 0.42019 | 0.01506 | 0.42847 | 0.01229 |
| 0.42141 | 0.01508 | 0.42939 | 0.01231 |
| 0.42264 | 0.0151 | 0.43031 | 0.01234 |
| 0.42295 | 0.01512 | 0.43154 | 0.01235 |
| 0.42448 | 0.01514 | 0.43246 | 0.01238 |
| 0.4254 | 0.01516 | 0.43368 | 0.0124 |
| 0.42663 | 0.01518 | 0.4343 | 0.01242 |
| 0.42694 | 0.0152 | 0.43552 | 0.01244 |
| 0.42847 | 0.01522 | 0.43583 | 0.01247 |
| 0.42939 | 0.01524 | 0.43736 | 0.01249 |
| 0.43062 | 0.01526 | 0.43859 | 0.01251 |
| 0.43123 | 0.01529 | 0.4389 | 0.01254 |
| 0.43184 | 0.0153 | 0.44166 | 0.0126 |
| 0.43338 | 0.01533 | 0.44227 | 0.01263 |
| 0.43399 | 0.01535 | 0.44288 | 0.01266 |
| 0.43522 | 0.01537 | 0.44411 | 0.0127 |
| 0.43614 | 0.0154 | 0.44503 | 0.01274 |
| 0.43736 | 0.01542 | 0.44595 | 0.01279 |
| 0.43767 | 0.01544 | 0.44748 | 0.01283 |
| 0.4389 | 0.01547 | 0.44779 | 0.01288 |
| 0.44012 | 0.0155 | 0.44871 | 0.01293 |
| 0.44135 | 0.01552 | 0.44932 | 0.013 |
| 0.44227 | 0.01554 | 0.45055 | 0.01306 |
| 0.44319 | 0.01557 | 0.45116 | 0.01313 |
| 0.44411 | 0.0156 | 0.45209 | 0.0132 |
| 0.44503 | 0.01563 | 0.45331 | 0.01329 |
| 0.44595 | 0.01565 | 0.45423 | 0.01337 |
| 0.44718 | 0.01569 | 0.45515 | 0.01346 |
| 0.44779 | 0.01572 | 0.45577 | 0.01356 |
| 0.44902 | 0.01575 | 0.45669 | 0.01367 |
| 0.44932 | 0.01578 | 0.45822 | 0.01377 |
| 0.45055 | 0.01581 | 0.45883 | 0.0139 |
| 0.45178 | 0.01585 | 0.45945 | 0.01401 |
| 0.4527 | 0.01588 | 0.46067 | 0.01415 |
| 0.45301 | 0.01592 | 0.46098 | 0.01428 |
| 0.45423 | 0.01596 | 0.46221 | 0.01442 |
| 0.45485 | 0.01601 | 0.46282 | 0.01457 |
| 0.45638 | 0.01605 | 0.46343 | 0.01472 |
| 0.4573 | 0.0161 | 0.46435 | 0.01487 |
| 0.45822 | 0.01615 | 0.46527 | 0.01504 |
| 0.45914 | 0.01621 | 0.4665 | 0.0152 |
| 0.46098 | 0.01627 | 0.46711 | 0.01536 |
| 0.46098 | 0.01634 | 0.46742 | 0.01554 |
| 0.4619 | 0.01642 | 0.46895 | 0.01571 |
| 0.46313 | 0.01651 | 0.46957 | 0.01588 |
| 0.46374 | 0.01659 | 0.47018 | 0.01607 |
| 0.46466 | 0.01667 | 0.4711 | 0.01625 |
| 0.46558 | 0.01676 | 0.47171 | 0.01643 |
| 0.4665 | 0.01685 | 0.47263 | 0.01661 |
| 0.46773 | 0.01695 | 0.47294 | 0.01681 |
| 0.46957 | 0.01712 | 0.47386 | 0.01699 |
| 0.46957 | 0.01721 | 0.47478 | 0.01719 |
| 0.47079 | 0.01731 | 0.47601 | 0.01739 |
| 0.4711 | 0.01741 | 0.47662 | 0.01758 |
| 0.47263 | 0.01752 | 0.47723 | 0.01777 |
| 0.47325 | 0.01762 | 0.47816 | 0.01798 |
| 0.47447 | 0.01773 | 0.47908 | 0.01817 |
| 0.47478 | 0.01784 | 0.47908 | 0.01792 |
| 0.47631 | 0.01796 | 0.47938 | 0.01814 |
| 0.47693 | 0.01809 | 0.48092 | 0.01835 |
| 0.47816 | 0.01821 | 0.48153 | 0.01855 |
| 0.47816 | 0.01795 | 0.48214 | 0.01875 |
| 0.47877 | 0.01813 | 0.48306 | 0.01895 |
| 0.47938 | 0.01827 | 0.48337 | 0.01915 |
| 0.48061 | 0.0184 | 0.48429 | 0.01933 |
| 0.48153 | 0.01854 | 0.48521 | 0.01954 |
| 0.48214 | 0.01867 | 0.48582 | 0.01973 |
| 0.48306 | 0.01881 | 0.48674 | 0.01992 |
| 0.48398 | 0.01895 | 0.48736 | 0.02013 |
| 0.48398 | 0.01908 | 0.48828 | 0.02031 |
| 0.48552 | 0.01922 | 0.48889 | 0.0205 |
| 0.48613 | 0.01935 | 0.48981 | 0.02068 |
| 0.48674 | 0.01948 | 0.49104 | 0.02086 |
| 0.48797 | 0.01962 | 0.49165 | 0.02105 |
| 0.4892 | 0.01974 | 0.49196 | 0.02124 |
| 0.4895 | 0.01987 | 0.49318 | 0.02141 |
| 0.49042 | 0.02001 | 0.49349 | 0.02158 |
| 0.49134 | 0.02014 | 0.49502 | 0.02175 |
| 0.49226 | 0.02027 | 0.49594 | 0.02192 |
| 0.49288 | 0.02041 | 0.49625 | 0.02209 |
| 0.4941 | 0.02054 | 0.49656 | 0.02225 |
| 0.49472 | 0.02067 | 0.49809 | 0.02241 |
| 0.49502 | 0.02081 | 0.4987 | 0.02257 |
| 0.49625 | 0.02095 | 0.49962 | 0.02273 |
| 0.49686 | 0.0211 | 0.50024 | 0.02289 |
| 0.49778 | 0.02122 | 0.50146 | 0.02302 |
| 0.4984 | 0.02136 | 0.50177 | 0.02316 |
| 0.49932 | 0.02149 | 0.5033 | 0.02332 |
| 0.50024 | 0.02165 | 0.50361 | 0.02344 |
| 0.50146 | 0.02176 | 0.50484 | 0.02356 |
| 0.50146 | 0.0219 | 0.50545 | 0.02369 |
| 0.503 | 0.02205 | 0.50637 | 0.02383 |
| 0.50392 | 0.02218 | 0.50668 | 0.02394 |
| 0.50453 | 0.02232 | 0.50821 | 0.02406 |
| 0.50515 | 0.02248 | 0.50883 | 0.02416 |
| 0.50607 | 0.02259 | 0.50975 | 0.02427 |
| 0.50668 | 0.02273 | 0.51036 | 0.02436 |
| 0.50791 | 0.02286 | 0.51159 | 0.02445 |
| 0.50852 | 0.02301 | 0.51251 | 0.02453 |
| 0.50852 | 0.02313 | 0.51343 | 0.02462 |
| 0.51036 | 0.02328 | 0.51435 | 0.02469 |
| 0.51097 | 0.0234 | 0.51496 | 0.02476 |
| 0.51189 | 0.02354 | 0.51619 | 0.02482 |
| 0.51189 | 0.02366 | 0.51711 | 0.02489 |
| 0.51373 | 0.02379 | 0.51833 | 0.02493 |
| 0.51435 | 0.0239 | 0.51833 | 0.02497 |
| 0.51496 | 0.02404 | 0.51987 | 0.02501 |
| 0.51619 | 0.02416 | 0.52109 | 0.02506 |
| 0.5168 | 0.0243 | 0.52171 | 0.02509 |
| 0.51741 | 0.02441 | 0.52263 | 0.0251 |
| 0.51864 | 0.02454 | 0.52416 | 0.02513 |
| 0.51956 | 0.02464 | 0.52477 | 0.02514 |
| 0.52017 | 0.02476 | 0.52569 | 0.02515 |
| 0.52079 | 0.02486 | 0.52692 | 0.02518 |
| 0.52201 | 0.02497 | 0.52753 | 0.02516 |
| 0.52293 | 0.02507 | 0.52876 | 0.02517 |
| 0.52355 | 0.02518 | 0.52968 | 0.02516 |
| 0.52447 | 0.02526 | 0.53091 | 0.02516 |
| 0.52508 | 0.02536 | 0.53122 | 0.02514 |
| 0.526 | 0.02544 | 0.53275 | 0.02513 |
| 0.52723 | 0.02554 | 0.53367 | 0.02511 |
| 0.52784 | 0.02562 | 0.53459 | 0.02509 |
| 0.52876 | 0.02571 | 0.53582 | 0.02507 |
| 0.52968 | 0.02578 | 0.53674 | 0.02503 |
| 0.5306 | 0.02584 | 0.53827 | 0.025 |
| 0.53152 | 0.02592 | 0.53919 | 0.02498 |
| 0.53275 | 0.02599 | 0.54011 | 0.02494 |
| 0.53336 | 0.02605 | 0.54103 | 0.0249 |
| 0.5349 | 0.02611 | 0.54195 | 0.02486 |
| 0.53551 | 0.02617 | 0.54287 | 0.02482 |
| 0.53674 | 0.02623 | 0.5441 | 0.02476 |
| 0.53704 | 0.02628 | 0.54563 | 0.02471 |
| 0.53766 | 0.02635 | 0.54624 | 0.02466 |
| 0.53858 | 0.02638 | 0.54716 | 0.0246 |
| 0.5398 | 0.02641 | 0.5487 | 0.0245 |
| 0.54134 | 0.02644 | 0.55054 | 0.02443 |
| 0.54195 | 0.02649 | 0.55176 | 0.02437 |
| 0.54256 | 0.02652 | 0.5533 | 0.02431 |
| 0.5441 | 0.02657 | 0.55391 | 0.02424 |
| 0.54471 | 0.02659 | 0.55483 | 0.02419 |
| 0.54624 | 0.02662 | 0.55606 | 0.02413 |
| 0.54655 | 0.02665 | 0.55698 | 0.02407 |
| 0.54716 | 0.02667 | 0.55821 | 0.024 |
| 0.5487 | 0.02668 | 0.55913 | 0.02394 |
| 0.54962 | 0.0267 | 0.56035 | 0.02388 |
| 0.55054 | 0.02672 | 0.56097 | 0.02382 |
| 0.55146 | 0.02673 | 0.5625 | 0.02378 |
| 0.55268 | 0.02675 | 0.56311 | 0.02371 |
| 0.55391 | 0.02676 | 0.56403 | 0.02367 |
| 0.55422 | 0.02678 | 0.56557 | 0.02359 |
| 0.55514 | 0.02679 | 0.56679 | 0.02355 |
| 0.55667 | 0.02679 | 0.56771 | 0.02349 |
| 0.55759 | 0.0268 | 0.56894 | 0.02343 |
| 0.55851 | 0.0268 | 0.57017 | 0.02338 |
| 0.56005 | 0.02682 | 0.57078 | 0.02335 |
| 0.56035 | 0.02682 | 0.57231 | 0.0233 |
| 0.56158 | 0.02682 | 0.57262 | 0.02325 |
| 0.5625 | 0.02682 | 0.57415 | 0.02321 |
| 0.56311 | 0.02684 | 0.57507 | 0.02317 |
| 0.56465 | 0.02684 | 0.57599 | 0.02312 |
| 0.56557 | 0.02685 | 0.57753 | 0.02308 |
| 0.56649 | 0.02686 | 0.57783 | 0.02305 |
| 0.56771 | 0.02685 | 0.57937 | 0.02301 |
| 0.56833 | 0.02687 | 0.58029 | 0.02297 |
| 0.57017 | 0.02688 | 0.5809 | 0.02295 |
| 0.57017 | 0.02686 | 0.58213 | 0.02292 |
| 0.57139 | 0.02688 | 0.58366 | 0.02289 |
| 0.57201 | 0.02689 | 0.58458 | 0.02285 |
| 0.57293 | 0.0269 | 0.5855 | 0.02283 |
| 0.57477 | 0.02692 | 0.58673 | 0.02281 |
| 0.57538 | 0.02693 | 0.58734 | 0.02278 |
| 0.5763 | 0.02692 | 0.58888 | 0.02275 |
| 0.57845 | 0.02695 | 0.58949 | 0.02275 |
| 0.57937 | 0.02696 | 0.59041 | 0.02272 |
| 0.58029 | 0.02696 | 0.59102 | 0.02269 |
| 0.58152 | 0.027 | 0.59256 | 0.02269 |
| 0.58152 | 0.02701 | 0.59378 | 0.02268 |
| 0.58305 | 0.02701 | 0.5947 | 0.02266 |
| 0.58428 | 0.02704 | 0.59562 | 0.02264 |
| 0.5855 | 0.02706 | 0.59685 | 0.02264 |
| 0.58612 | 0.02707 | 0.59777 | 0.02262 |
| 0.58734 | 0.02709 | 0.599 | 0.02263 |
| 0.58796 | 0.02711 | 0.59961 | 0.0226 |
| 0.58918 | 0.02712 | 0.60084 | 0.0226 |
| 0.59041 | 0.02714 | 0.60268 | 0.02259 |
| 0.59102 | 0.02719 | 0.60268 | 0.0226 |
| 0.59194 | 0.02719 | 0.60421 | 0.0226 |
| 0.59286 | 0.02722 | 0.60482 | 0.0226 |
| 0.59409 | 0.02725 | 0.60605 | 0.0226 |
| 0.59501 | 0.02727 | 0.60667 | 0.0226 |
| 0.59532 | 0.02729 | 0.60759 | 0.0226 |
| 0.59685 | 0.02732 | 0.60881 | 0.02263 |
| 0.59746 | 0.02736 | 0.60973 | 0.02263 |
| 0.59869 | 0.02738 | 0.61096 | 0.02263 |
| 0.59992 | 0.02743 | 0.61157 | 0.02265 |
| 0.60022 | 0.02746 | 0.6128 | 0.02265 |
| 0.60145 | 0.02749 | 0.61403 | 0.02267 |
| 0.60206 | 0.02752 | 0.61464 | 0.02269 |
| 0.6036 | 0.02754 | 0.61556 | 0.0227 |
| 0.60482 | 0.02758 | 0.61679 | 0.0227 |
| 0.60544 | 0.02762 | 0.61771 | 0.02273 |
| 0.60636 | 0.02766 | 0.61801 | 0.02275 |
| 0.60759 | 0.02771 | 0.61985 | 0.02278 |
| 0.60789 | 0.02775 | 0.62047 | 0.02279 |
| 0.60943 | 0.02779 | 0.622 | 0.02283 |
| 0.61004 | 0.02783 | 0.62261 | 0.02284 |
| 0.61127 | 0.02787 | 0.62323 | 0.02287 |
| 0.61188 | 0.0279 | 0.62415 | 0.0229 |
| 0.61311 | 0.02796 | 0.62629 | 0.02293 |
| 0.61403 | 0.02801 | 0.6266 | 0.02296 |
| 0.61525 | 0.02806 | 0.62721 | 0.023 |
| 0.61587 | 0.02811 | 0.62844 | 0.02305 |
| 0.61709 | 0.02814 | 0.62936 | 0.02308 |
| 0.61771 | 0.02819 | 0.63028 | 0.02311 |
| 0.61863 | 0.02825 | 0.6312 | 0.02317 |
| 0.61985 | 0.02831 | 0.63181 | 0.0232 |
| 0.62047 | 0.02836 | 0.63366 | 0.02323 |
| 0.62169 | 0.02842 | 0.63396 | 0.02329 |
| 0.62261 | 0.02847 | 0.63519 | 0.02334 |
| 0.62323 | 0.02853 | 0.63611 | 0.0234 |
| 0.62415 | 0.02859 | 0.63672 | 0.02345 |
| 0.62507 | 0.02865 | 0.63734 | 0.02351 |
| 0.62599 | 0.0287 | 0.63887 | 0.02357 |
| 0.6266 | 0.02878 | 0.6401 | 0.02363 |
| 0.62813 | 0.02884 | 0.6404 | 0.02369 |
| 0.62875 | 0.0289 | 0.64132 | 0.02375 |
| 0.62967 | 0.02896 | 0.64224 | 0.02383 |
| 0.63028 | 0.02904 | 0.64286 | 0.02389 |
| 0.63181 | 0.02909 | 0.64408 | 0.02395 |
| 0.63274 | 0.02918 | 0.64531 | 0.02403 |
| 0.63304 | 0.02924 | 0.64623 | 0.02411 |
| 0.63396 | 0.02933 | 0.64684 | 0.0242 |
| 0.63519 | 0.02939 | 0.64807 | 0.02427 |
| 0.6358 | 0.02948 | 0.64838 | 0.02435 |
| 0.63703 | 0.02955 | 0.6496 | 0.02443 |
| 0.63764 | 0.02963 | 0.65052 | 0.02451 |
| 0.63887 | 0.02972 | 0.65175 | 0.02462 |
| 0.63979 | 0.02981 | 0.65267 | 0.0247 |
| 0.6401 | 0.02988 | 0.65298 | 0.02479 |
| 0.64194 | 0.02997 | 0.65451 | 0.02489 |
| 0.64224 | 0.03006 | 0.65451 | 0.025 |
| 0.64286 | 0.03016 | 0.65574 | 0.02509 |
| 0.64378 | 0.03024 | 0.65696 | 0.02521 |
| 0.645 | 0.03033 | 0.65788 | 0.02531 |
| 0.64562 | 0.03044 | 0.6585 | 0.02541 |
| 0.64654 | 0.03054 | 0.65942 | 0.02552 |
| 0.64776 | 0.03064 | 0.66003 | 0.02565 |
| 0.64838 | 0.03074 | 0.66126 | 0.02576 |
| 0.64899 | 0.03084 | 0.66218 | 0.02589 |
| 0.65022 | 0.03093 | 0.66279 | 0.02602 |
| 0.65052 | 0.03106 | 0.66371 | 0.02614 |
| 0.65175 | 0.03115 | 0.66433 | 0.02626 |
| 0.65236 | 0.03127 | 0.66525 | 0.0264 |
| 0.65359 | 0.03138 | 0.66617 | 0.02654 |
| 0.6539 | 0.03151 | 0.66739 | 0.02668 |
| 0.65512 | 0.03163 | 0.6677 | 0.02681 |
| 0.65635 | 0.03174 | 0.66831 | 0.02695 |
| 0.65696 | 0.03186 | 0.66954 | 0.02709 |
| 0.65758 | 0.03198 | 0.67077 | 0.02725 |
| 0.65819 | 0.03211 | 0.67169 | 0.0274 |
| 0.65973 | 0.03224 | 0.67169 | 0.02755 |
| 0.66034 | 0.03237 | 0.67291 | 0.0277 |
| 0.66095 | 0.0325 | 0.67353 | 0.02787 |
| 0.66157 | 0.03264 | 0.67445 | 0.02803 |
| 0.66249 | 0.03278 | 0.67506 | 0.0282 |
| 0.66371 | 0.03292 | 0.67629 | 0.02837 |
| 0.66433 | 0.03306 | 0.67659 | 0.02855 |
| 0.66525 | 0.0332 | 0.67782 | 0.02871 |
| 0.66647 | 0.03335 | 0.67874 | 0.02889 |
| 0.66617 | 0.0335 | 0.67935 | 0.02907 |
| 0.66801 | 0.03366 | 0.67997 | 0.02924 |
| 0.66801 | 0.0338 | 0.68119 | 0.02944 |
| 0.66954 | 0.03398 | 0.6815 | 0.02963 |
| 0.67015 | 0.03413 | 0.68242 | 0.02982 |
| 0.67077 | 0.03428 | 0.68334 | 0.03001 |
| 0.67138 | 0.03444 | 0.68396 | 0.0302 |
| 0.67199 | 0.03461 | 0.68457 | 0.0304 |
| 0.67322 | 0.03479 | 0.68549 | 0.03061 |
| 0.67383 | 0.03496 | 0.68641 | 0.03081 |
| 0.67445 | 0.03513 | 0.68702 | 0.03101 |
| 0.67537 | 0.03529 | 0.68764 | 0.03123 |
| 0.67629 | 0.03547 | 0.68856 | 0.03143 |
| 0.67659 | 0.03565 | 0.68917 | 0.03165 |
| 0.67782 | 0.03582 | 0.68978 | 0.03185 |
| 0.67782 | 0.03601 | 0.6907 | 0.03208 |
| 0.67905 | 0.03621 | 0.69162 | 0.03231 |
| 0.67997 | 0.03639 | 0.69224 | 0.03252 |
| 0.68119 | 0.03658 | 0.69316 | 0.03275 |
| 0.6815 | 0.03678 | 0.69408 | 0.03298 |
| 0.68211 | 0.03698 | 0.69438 | 0.0332 |
| 0.68303 | 0.03717 | 0.695 | 0.03344 |
| 0.68365 | 0.03738 | 0.69592 | 0.03367 |
| 0.68457 | 0.03758 | 0.69622 | 0.03391 |
| 0.68488 | 0.03778 | 0.69745 | 0.03415 |
| 0.6858 | 0.03799 | 0.69806 | 0.0344 |
| 0.68641 | 0.0382 | 0.69898 | 0.03464 |
| 0.68764 | 0.03841 | 0.6999 | 0.03488 |
| 0.68794 | 0.03861 | 0.70052 | 0.03512 |
| 0.68886 | 0.03884 | 0.70113 | 0.03538 |
| 0.68948 | 0.03905 | 0.70174 | 0.03563 |
| 0.6907 | 0.03926 | 0.70205 | 0.03587 |
| 0.69101 | 0.0395 | 0.70297 | 0.03614 |
| 0.69162 | 0.03971 | 0.70358 | 0.03639 |
| 0.69193 | 0.03995 | 0.7045 | 0.03665 |
| 0.69285 | 0.04017 | 0.70512 | 0.03691 |
| 0.69377 | 0.04041 | 0.70604 | 0.03716 |
| 0.69408 | 0.04062 | 0.70665 | 0.03742 |
| 0.695 | 0.04086 | 0.70726 | 0.0377 |
| 0.69592 | 0.0411 | 0.70757 | 0.03798 |
| 0.69653 | 0.04133 | 0.7091 | 0.03823 |
| 0.69714 | 0.04158 | 0.7088 | 0.03852 |
| 0.69806 | 0.04181 | 0.7091 | 0.03877 |
| 0.69868 | 0.04207 | 0.71064 | 0.03906 |
| 0.69898 | 0.04231 | 0.71125 | 0.03933 |
| 0.6999 | 0.04253 | 0.71217 | 0.03961 |
| 0.70052 | 0.0428 | 0.71279 | 0.03989 |
| 0.70113 | 0.04305 | 0.71309 | 0.04017 |
| 0.70205 | 0.04331 | 0.71401 | 0.04045 |
| 0.70236 | 0.04356 | 0.71463 | 0.04074 |
| 0.70328 | 0.04382 | 0.71555 | 0.04102 |
| 0.7042 | 0.04407 | 0.71585 | 0.04131 |
| 0.70512 | 0.04433 | 0.71677 | 0.0416 |
| 0.70542 | 0.04458 | 0.71708 | 0.04187 |
| 0.70604 | 0.04485 | 0.71739 | 0.04217 |
| 0.70665 | 0.04512 | 0.71861 | 0.04248 |
| 0.70665 | 0.04539 | 0.71953 | 0.04277 |
| 0.70757 | 0.04566 | 0.72015 | 0.04307 |
| 0.7088 | 0.04591 | 0.72045 | 0.04336 |
| 0.7091 | 0.0462 | 0.72137 | 0.04365 |
| 0.70972 | 0.04646 | 0.72199 | 0.04395 |
| 0.71064 | 0.04672 | 0.72291 | 0.04424 |
| 0.71156 | 0.04701 | 0.72352 | 0.04454 |
| 0.71217 | 0.04728 | 0.72352 | 0.04485 |
| 0.71248 | 0.04755 | 0.72505 | 0.04516 |
| 0.71309 | 0.04783 | 0.72505 | 0.04547 |
| 0.7134 | 0.04811 | 0.72567 | 0.04575 |
| 0.71463 | 0.0484 | 0.72659 | 0.04607 |
| 0.71493 | 0.04867 | 0.7272 | 0.04638 |
| 0.71555 | 0.04896 | 0.72781 | 0.04668 |
| 0.71616 | 0.04924 | 0.72843 | 0.04699 |
| 0.71677 | 0.04953 | 0.72873 | 0.04731 |
| 0.71708 | 0.04982 | 0.72965 | 0.04761 |
| 0.718 | 0.05012 | 0.73027 | 0.04793 |
| 0.71923 | 0.0504 | 0.73119 | 0.04823 |
| 0.71923 | 0.05069 | 0.73149 | 0.04855 |
| 0.71953 | 0.05099 | 0.73241 | 0.04886 |
| 0.72107 | 0.05128 | 0.73303 | 0.04918 |
| 0.72107 | 0.05158 | 0.73364 | 0.04951 |
| 0.72199 | 0.05186 | 0.73425 | 0.0498 |
| 0.7226 | 0.05215 | 0.73487 | 0.05013 |
| 0.72291 | 0.05246 | 0.73548 | 0.05045 |
| 0.72383 | 0.05277 | 0.73579 | 0.05077 |
| 0.72444 | 0.05305 | 0.73702 | 0.05109 |
| 0.72444 | 0.05336 | 0.73763 | 0.05142 |
| 0.72567 | 0.05366 | 0.73732 | 0.05172 |
| 0.72628 | 0.05397 | 0.73886 | 0.05205 |
| 0.72659 | 0.05426 | 0.73886 | 0.05239 |
| 0.7272 | 0.05458 | 0.73947 | 0.05271 |
| 0.72781 | 0.05488 | 0.74039 | 0.05303 |
| 0.72843 | 0.05518 | 0.741 | 0.05337 |
| 0.72904 | 0.05551 | 0.74131 | 0.05368 |
| 0.72935 | 0.05581 | 0.74192 | 0.054 |
| 0.73027 | 0.0561 | 0.74254 | 0.05434 |
| 0.73057 | 0.05644 | 0.74284 | 0.05467 |
| 0.7318 | 0.05675 | 0.74407 | 0.055 |
| 0.73149 | 0.05705 | 0.74438 | 0.05533 |
| 0.73241 | 0.05737 | 0.74499 | 0.05566 |
| 0.73333 | 0.05769 | 0.7456 | 0.05599 |
| 0.73333 | 0.058 | 0.74622 | 0.05633 |
| 0.73456 | 0.05831 | 0.74714 | 0.05665 |
| 0.73517 | 0.05864 | 0.74775 | 0.05699 |
| 0.73548 | 0.05896 | 0.74836 | 0.05733 |
| 0.7361 | 0.05927 | 0.74928 | 0.05765 |
| 0.7364 | 0.05958 | 0.74928 | 0.05799 |
| 0.73763 | 0.0599 | 0.7499 | 0.05832 |
| 0.73824 | 0.06023 | 0.75082 | 0.05865 |
| 0.73794 | 0.06055 | 0.75112 | 0.059 |
| 0.73886 | 0.06088 | 0.75143 | 0.05934 |
| 0.73947 | 0.06121 | 0.75235 | 0.05968 |
| 0.74039 | 0.06153 | 0.75296 | 0.06001 |
| 0.741 | 0.06186 | 0.75358 | 0.06035 |
| 0.74162 | 0.06217 | 0.75388 | 0.06068 |
| 0.74223 | 0.0625 | 0.7545 | 0.06103 |
| 0.74192 | 0.06282 | 0.75542 | 0.06137 |
| 0.74284 | 0.06315 | 0.75603 | 0.06171 |
| 0.74376 | 0.06348 | 0.75664 | 0.06207 |
| 0.74438 | 0.06381 | 0.75726 | 0.06239 |
| 0.74499 | 0.06414 | 0.75756 | 0.06275 |
| 0.74499 | 0.06447 | 0.75879 | 0.06308 |
| 0.7456 | 0.0648 | 0.75879 | 0.06342 |
| 0.74622 | 0.06514 | 0.75971 | 0.06377 |
| 0.74714 | 0.06548 | 0.76032 | 0.0641 |
| 0.74744 | 0.06579 | 0.76032 | 0.06445 |
| 0.74775 | 0.06614 | 0.76155 | 0.06479 |
| 0.74867 | 0.06645 | 0.76186 | 0.06515 |
| 0.74928 | 0.06679 | 0.76278 | 0.06549 |
| 0.74959 | 0.06713 | 0.76309 | 0.06584 |
| 0.75051 | 0.06748 | 0.7637 | 0.06618 |
| 0.75112 | 0.0678 | 0.76431 | 0.06655 |
| 0.75143 | 0.06815 | 0.76493 | 0.06687 |
| 0.75204 | 0.06849 | 0.76554 | 0.06724 |
| 0.75235 | 0.06882 | 0.76585 | 0.06758 |
| 0.75266 | 0.06916 | 0.76677 | 0.06794 |
| 0.75358 | 0.06951 | 0.76707 | 0.06829 |
| 0.7548 | 0.06984 | 0.76769 | 0.06865 |
| 0.7545 | 0.07017 | 0.7683 | 0.069 |
| 0.75511 | 0.07052 | 0.7683 | 0.06935 |
| 0.75603 | 0.07086 | 0.76953 | 0.06971 |
| 0.75695 | 0.0712 | 0.77014 | 0.07006 |
| 0.75664 | 0.07155 | 0.77075 | 0.0704 |
| 0.75756 | 0.07189 | 0.77106 | 0.07077 |
| 0.75818 | 0.07224 | 0.77167 | 0.07111 |
| 0.75879 | 0.07257 | 0.77259 | 0.07146 |
| 0.7594 | 0.07293 | 0.7729 | 0.07182 |
| 0.75971 | 0.07327 | 0.77351 | 0.07217 |
| 0.76002 | 0.07362 | 0.77413 | 0.07254 |
| 0.76063 | 0.07397 | 0.77474 | 0.0729 |
| 0.76155 | 0.0743 | 0.77597 | 0.0736 |
| 0.76155 | 0.07466 | 0.77658 | 0.07397 |
| 0.76278 | 0.07499 | 0.77719 | 0.07433 |
| 0.76278 | 0.07534 | 0.77781 | 0.07467 |
| 0.76401 | 0.07569 | 0.77811 | 0.07504 |
| 0.76431 | 0.07604 | 0.77842 | 0.07539 |
| 0.76462 | 0.0764 | 0.77934 | 0.07575 |
| 0.76523 | 0.07674 | 0.77965 | 0.07611 |
| 0.76585 | 0.07709 | 0.78026 | 0.07647 |
| 0.76646 | 0.07744 | 0.78087 | 0.07681 |
| 0.76677 | 0.07779 | 0.78179 | 0.07717 |
| 0.76738 | 0.07814 | 0.78241 | 0.07754 |
| 0.7683 | 0.07849 | 0.78241 | 0.07789 |
| 0.76799 | 0.07884 | 0.78333 | 0.07824 |
| 0.76861 | 0.07919 | 0.78425 | 0.0786 |
| 0.76953 | 0.07954 | 0.78425 | 0.07895 |
| 0.77014 | 0.0799 | 0.78486 | 0.07933 |
| 0.77045 | 0.08025 | 0.78517 | 0.07968 |
| 0.77106 | 0.0806 | 0.78578 | 0.08006 |
| 0.77198 | 0.08096 | 0.78639 | 0.08042 |
| 0.77229 | 0.0813 | 0.78701 | 0.08079 |
| 0.77321 | 0.08165 | 0.78732 | 0.08115 |
| 0.77351 | 0.08202 | 0.78854 | 0.08152 |
| 0.77351 | 0.08236 | 0.78916 | 0.08188 |
| 0.77443 | 0.08272 | 0.78885 | 0.08222 |
| 0.77474 | 0.08309 | 0.78977 | 0.0826 |
| 0.77597 | 0.08344 | 0.79069 | 0.08296 |
| 0.77658 | 0.08379 | 0.7913 | 0.08331 |
| 0.77658 | 0.08415 | 0.79192 | 0.08368 |
| 0.77719 | 0.0845 | 0.79253 | 0.08404 |
| 0.7775 | 0.08487 | 0.79284 | 0.0844 |
| 0.77811 | 0.08522 | 0.79345 | 0.08476 |
| 0.77873 | 0.08557 | 0.79376 | 0.08512 |
| 0.77873 | 0.08593 | 0.79437 | 0.0855 |
| 0.77934 | 0.08629 | 0.7956 | 0.08584 |
| 0.78057 | 0.08665 | 0.79529 | 0.08621 |
| 0.78057 | 0.08699 | 0.79621 | 0.08656 |
| 0.78179 | 0.08737 | 0.79652 | 0.08693 |
| 0.7821 | 0.08809 | 0.79713 | 0.08731 |
| 0.78302 | 0.08844 | 0.79774 | 0.08766 |
| 0.78333 | 0.08881 | 0.79866 | 0.08802 |
| 0.78394 | 0.08917 | 0.79897 | 0.08838 |
| 0.78455 | 0.08953 | 0.79989 | 0.08877 |
| 0.78486 | 0.0899 | 0.8002 | 0.08913 |
| 0.78547 | 0.09025 | 0.8005 | 0.0895 |
| 0.78609 | 0.09061 | 0.80142 | 0.08985 |
| 0.7867 | 0.09097 | 0.80173 | 0.09023 |
| 0.7867 | 0.09135 | 0.80234 | 0.09061 |
| 0.78732 | 0.09171 | 0.80265 | 0.09096 |
| 0.78824 | 0.09207 | 0.80357 | 0.09132 |
| 0.78885 | 0.09242 | 0.80388 | 0.09169 |
| 0.78946 | 0.0928 | 0.80418 | 0.09206 |
| 0.78977 | 0.09314 | 0.8051 | 0.09242 |
| 0.79008 | 0.09351 | 0.80541 | 0.09278 |
| 0.79069 | 0.09387 | 0.80602 | 0.09315 |
| 0.79161 | 0.09424 | 0.80725 | 0.09353 |
| 0.79192 | 0.09461 | 0.80756 | 0.09387 |
| 0.79222 | 0.09497 | 0.80756 | 0.09424 |
| 0.79284 | 0.09534 | 0.80817 | 0.09459 |
| 0.79376 | 0.0957 | 0.80909 | 0.09496 |
| 0.79376 | 0.09607 | 0.8094 | 0.09533 |
| 0.79437 | 0.09643 | 0.81001 | 0.0957 |
| 0.79468 | 0.0968 | 0.81062 | 0.09607 |
| 0.7959 | 0.09716 | 0.81093 | 0.09645 |
| 0.79621 | 0.09753 | 0.81154 | 0.0968 |
| 0.79652 | 0.09789 | 0.81185 | 0.09716 |
| 0.79713 | 0.09827 | 0.81246 | 0.09753 |
| 0.79744 | 0.09863 | 0.81369 | 0.0979 |
| 0.79774 | 0.09899 | 0.814 | 0.09826 |
| 0.79866 | 0.09937 | 0.81461 | 0.09864 |
| 0.79897 | 0.09973 | 0.81492 | 0.099 |
| 0.79958 | 0.10009 | 0.81523 | 0.09937 |
| 0.8002 | 0.10046 | 0.81584 | 0.09975 |
| 0.8005 | 0.10083 | 0.81645 | 0.1001 |
| 0.80112 | 0.10119 | 0.81707 | 0.10047 |
| 0.80204 | 0.10157 | 0.81768 | 0.10083 |
| 0.80204 | 0.10194 | 0.81799 | 0.10121 |
| 0.80296 | 0.1023 | 0.81891 | 0.10158 |
| 0.80326 | 0.10267 | 0.81921 | 0.10194 |
| 0.80388 | 0.10303 | 0.82013 | 0.10232 |
| 0.80357 | 0.1034 | 0.82044 | 0.10269 |
| 0.8051 | 0.10376 | 0.82075 | 0.10307 |
| 0.80602 | 0.10413 | 0.82105 | 0.10343 |
| 0.80572 | 0.10451 | 0.82197 | 0.1038 |
| 0.80664 | 0.10487 | 0.82289 | 0.10417 |
| 0.80694 | 0.10524 | 0.82351 | 0.10454 |
| 0.80725 | 0.10562 | 0.82351 | 0.1049 |
| 0.80817 | 0.106 | 0.82412 | 0.10527 |
| 0.80878 | 0.10637 | 0.82443 | 0.10565 |
| 0.80848 | 0.10674 | 0.82535 | 0.10604 |
| 0.8097 | 0.1071 | 0.82596 | 0.10643 |
| 0.81001 | 0.10748 | 0.82627 | 0.10679 |
| 0.81032 | 0.10785 | 0.82688 | 0.10714 |
| 0.81032 | 0.10823 | 0.82749 | 0.10753 |
| 0.81154 | 0.10859 | 0.8278 | 0.1079 |
| 0.81185 | 0.10897 | 0.82872 | 0.10825 |
| 0.81277 | 0.10934 | 0.82872 | 0.10862 |
| 0.81308 | 0.1097 | 0.82995 | 0.109 |
| 0.81277 | 0.11008 | 0.82995 | 0.10937 |
| 0.814 | 0.11046 | 0.83087 | 0.10974 |
| 0.81461 | 0.11082 | 0.83148 | 0.11012 |
| 0.81492 | 0.11119 | 0.83179 | 0.11048 |
| 0.81584 | 0.11157 | 0.83209 | 0.11087 |
| 0.81584 | 0.11194 | 0.83271 | 0.11124 |
| 0.81645 | 0.11232 | 0.83301 | 0.1116 |
| 0.81737 | 0.11268 | 0.83455 | 0.11197 |
| 0.81707 | 0.11306 | 0.83424 | 0.11235 |
| 0.81799 | 0.11343 | 0.83516 | 0.11271 |
| 0.8186 | 0.11381 | 0.83577 | 0.11308 |
| 0.81921 | 0.11418 | 0.83669 | 0.11347 |
| 0.81983 | 0.11455 | 0.83669 | 0.11385 |
| 0.82044 | 0.11493 | 0.83731 | 0.11421 |
| 0.82075 | 0.11529 | 0.83761 | 0.11459 |
| 0.82105 | 0.11567 | 0.83854 | 0.11496 |
| 0.82197 | 0.11603 | 0.83915 | 0.11535 |
| 0.82228 | 0.11641 | 0.83884 | 0.11572 |
| 0.82259 | 0.1168 | 0.84007 | 0.11611 |
| 0.82381 | 0.11717 | 0.84068 | 0.11649 |
| 0.82412 | 0.11754 | 0.8413 | 0.11685 |
| 0.82412 | 0.11792 | 0.8416 | 0.11724 |
| 0.82473 | 0.11829 | 0.84222 | 0.11761 |
| 0.82565 | 0.11866 | 0.84314 | 0.11798 |
| 0.82565 | 0.11904 | 0.84375 | 0.11871 |
| 0.82627 | 0.11941 | 0.84406 | 0.1191 |
| 0.82657 | 0.11979 | 0.84498 | 0.11949 |
| 0.82719 | 0.12017 | 0.84559 | 0.11986 |
| 0.8278 | 0.12054 | 0.8462 | 0.12026 |
| 0.82811 | 0.1209 | 0.84651 | 0.12064 |
| 0.82903 | 0.12127 | 0.84712 | 0.12101 |
| 0.82903 | 0.12165 | 0.84743 | 0.12139 |
| 0.82964 | 0.12203 | 0.84804 | 0.12176 |
| 0.83025 | 0.12241 | 0.84896 | 0.12212 |
| 0.83056 | 0.1228 | 0.84927 | 0.1225 |
| 0.83087 | 0.12317 | 0.84958 | 0.12289 |
| 0.83179 | 0.12356 | 0.85019 | 0.12328 |
| 0.83209 | 0.12395 | 0.8508 | 0.12366 |
| 0.8324 | 0.1243 | 0.85142 | 0.12404 |
| 0.83332 | 0.12469 | 0.85203 | 0.12441 |
| 0.83393 | 0.12507 | 0.85172 | 0.12427 |
| 0.83424 | 0.12544 | 0.85142 | 0.12384 |

Data for Fig. 8:

| FeOOH/GO-1 | | FeOOH/GO-2 | |
| --- | --- | --- | --- |
| Zre / ohm | Zim / ohm | Zre / ohm | Zim / ohm |
| 1.07817 | 0.16604 | 0.90853 | 0.10897 |
| 1.10342 | 0.18479 | 0.92483 | 0.12428 |
| 1.12978 | 0.20305 | 0.93915 | 0.13743 |
| 1.15946 | 0.22302 | 0.95845 | 0.15533 |
| 1.19304 | 0.24309 | 0.97658 | 0.17173 |
| 1.22948 | 0.26253 | 1.00004 | 0.18929 |
| 1.27416 | 0.28416 | 1.02497 | 0.21164 |
| 1.31749 | 0.30107 | 1.05408 | 0.23468 |
| 1.37012 | 0.3191 | 1.08677 | 0.26057 |
| 1.42645 | 0.33221 | 1.12593 | 0.28849 |
| 1.48087 | 0.34004 | 1.16676 | 0.3156 |
| 1.54113 | 0.3452 | 1.21547 | 0.34467 |
| 1.6009 | 0.34473 | 1.27402 | 0.37439 |
| 1.65583 | 0.34077 | 1.33246 | 0.40134 |
| 1.711 | 0.33581 | 1.4004 | 0.42473 |
| 1.76173 | 0.3263 | 1.49303 | 0.45149 |
| 1.80576 | 0.31951 | 1.55364 | 0.46327 |
| 1.85283 | 0.31022 | 1.63235 | 0.46707 |
| 1.89454 | 0.30503 | 1.71565 | 0.47122 |
| 1.93632 | 0.30114 | 1.7884 | 0.46798 |
| 1.97496 | 0.29804 | 1.86575 | 0.45869 |
| 2.01346 | 0.29892 | 1.93596 | 0.45005 |
| 2.05121 | 0.30191 | 1.99637 | 0.43777 |
| 2.09199 | 0.30836 | 2.05808 | 0.42628 |
| 2.13359 | 0.3184 | 2.1228 | 0.42113 |
| 2.17424 | 0.3258 | 2.17541 | 0.4144 |
| 2.21934 | 0.33763 | 2.23399 | 0.41102 |
| 2.27447 | 0.35389 | 2.28629 | 0.40543 |
| 2.3206 | 0.36364 | 2.3262 | 0.41036 |
| 2.37044 | 0.37963 | 2.3829 | 0.41181 |
| 2.43013 | 0.39234 | 2.41933 | 0.42379 |
| 2.48086 | 0.39761 | 2.46276 | 0.44919 |
| 2.53823 | 0.40943 | 2.49521 | 0.48008 |
| 2.58817 | 0.42371 | 2.53417 | 0.52392 |
| 2.63594 | 0.44804 | 2.56804 | 0.57613 |
| 2.68763 | 0.46743 | 2.58798 | 0.66528 |
| 2.72116 | 0.50847 | 2.63814 | 0.76213 |
| 2.76028 | 0.54523 | 2.65849 | 0.92266 |
| 2.80839 | 0.61683 | 2.67709 | 1.06971 |
| 2.83446 | 0.71977 | 2.75478 | 1.27602 |
| 2.84774 | 0.82361 | 2.79244 | 1.6146 |
| 2.90462 | 0.95236 | 2.7731 | 1.94766 |
| 2.95747 | 1.17122 | 2.86222 | 2.32121 |
| 2.94704 | 1.39684 | 2.99387 | 2.90908 |
| 2.98088 | 1.67572 | 3.03514 | 3.68623 |
| 3.05354 | 2.02143 | 3.02379 | 4.5681 |
